# MdGSTF6, activated by MdMYB1, plays an essential role in anthocyanin accumulation in apple

**DOI:** 10.1038/s41438-019-0118-6

**Published:** 2019-03-01

**Authors:** Shenghui Jiang, Min Chen, Naibo He, Xiaoliu Chen, Nan Wang, Qingguo Sun, Tianliang Zhang, Haifeng Xu, Hongcheng Fang, Yicheng Wang, Zongying Zhang, Shujing Wu, Xuesen Chen

**Affiliations:** 1 College of Horticulture Science and Engineering, State Key Laboratory of Crop Biology, Collaborative Innovation Center of Fruit & Vegetable Quality and Efficient Production in Shandong, Shandong Agricultural University, 61 Daizong Road, Tai’an, 271018 China; 2 National Oceanographic Center, 88 Xuzhou Road, Qingdao, 266071 China

**Keywords:** Transcription, Secondary metabolism

## Abstract

Anthocyanins are biosynthesized on the cytosolic surface of the endoplasmic reticulum and then transported into the vacuole for storage. Glutathione *S*-transferases (GSTs) are considered to be responsible for the transport of anthocyanins into the vacuole. However, the regulatory mechanisms of GSTs in plants are still unclear. Here, we performed a genome-wide analysis and identified 69 *GST* genes in apple. The expression of *MdGSTF6* was positively correlated with the anthocyanin content (*r* = 0.949) during ‘Yanfu 8’ fruit development. The overexpression of *MdGSTF6* in the *Arabidopsis thaliana tt19* mutant resulted in seedlings of 35S::*MdGSTF6*-GFP/*tt19* that could accumulate anthocyanin and rescue its phenotype, suggesting that MdGSTF6 was an anthocyanin transporter. The silencing of *MdGSTF6* affected anthocyanin accumulation in apple fruit. Moreover, the knockdown of *MdGSTF6* by RNA interference in cultured ‘Gala’ seedlings inhibited anthocyanin accumulation. The interaction experiments showed that MdMYB1 could bind directly to the *MdGSTF6* promoter to transcriptionally activate its expression. Collectively, our results demonstrate that *MdGSTF6* encodes an important GST transporter of anthocyanins in apple fruit and provide evidence for the associated regulatory mechanisms. Therefore, MdMYB1 can not only regulate anthocyanin synthesis, but also control the transport of anthocyanin in apples. This information may be useful for further clarifying the regulation of anthocyanin transport in apple.

## Intracellular transport: Identifying how apple pigment is sent for storage

The mystery of how pigmenting anthocyanins are transported to intracellular storage is starting to be revealed. Alongside giving fruit their color, anthocyanins confer benefits to plant and human health. Glutathione *S*-transferases (GSTs) are responsible for transporting anthocyanins to storage vacuoles, yet research is lacking on how GSTs are regulated. Xuesen Chen, from China’s Shandong Agricultural University, and his team analyzed the activity of 23 GST genes in apple, finding one, *MdGSTF6*, was most highly active in the fruit-coloration stage of apple development. *MdGSTF6* expression correlated with fruit anthocyanin levels and also restored anthocyanin levels in transport-suppressed plants, confirming its function. Chen’s team then discovered that *MdGSTF6* expression is activated by the protein MdMYB1. The *MdMYB1* gene is the major regulatory gene in anthocyanin synthesis, and is now revealed to also influence their transport.

## Introduction

Apple is an important fruit crop grown in temperate zones worldwide. Fruit color is an important factor in terms of consumer preference. Therefore, fruit coloration is one of the most important agronomic traits for apple fruit quality. The fruit color of apples is determined by the types and concentrations of anthocyanins^1,2^, as in other fruits such as pear^3^, grape^4,5^, and strawberry^6,7^. Anthocyanins play important roles in resistance to pathogens, seed dispersal, and protection against ultraviolet radiation^8–10^. They are also beneficial for human health and can protect against certain diseases^11–13^. Three different anthocyanins have been detected in apple, and all of them are of the cyanidin type, conjugated with diverse sugars^14^. Among them, cyanidin-3-galactoside is the predominant anthocyanin in many apple cultivars^15^.

The enzymes that catalyze anthocyanin biosynthesis function in the flavonoid pathway. The genes related to anthocyanin synthesis include *CHS* (encoding chalcone synthase), *CHI* (encoding chalcone isomerase), *F3H* (encoding flavanone 3- hydroxylase), *DFR* (encoding dihydroflavonol 4-reductase), *ANS* (encoding anthocyanidin synthase), and *UFGT* (encoding UDP-glucose: flavonoid-3-*O*-glucosyltransferase). Among them, *CHS*, *CHI*, and *F3H*, the so-called early biosynthetic genes, are involved in the production of precursors (i.e., dihydroflavonols). The late biosynthetic genes, including *DFR*, *ANS*, and *UFGT*, are involved in the production of colored anthocyanins^16^. Anthocyanin biosynthesis is regulated by the MYB-bHLH-WD40 (MBW) protein complex, which consists of the MYB and bHLH transcription factors and one WD-40 protein^17^. Two MYB transcription factors, MdMYB1 and MdMYBA, have been shown to regulate anthocyanin biosynthesis in apple peel^18,19^. MdMYB1 binds to the *MdDFR* and *MdUFGT* promoters, while MdMYBA binds to the *MdANS* promoter. Another MYB, MdMYB10, is responsible for the red flesh color of red-fleshed apple fruits. It can interact with MdbHLH3/33 to increase the activity of the *MdDFR* promoter^20^. MdbHLH3 is phosphorylated at low temperature, thereby enhancing its transcriptional activation activity, ultimately leading to anthocyanin accumulation^21^. In apple, MdTTG1 does not bind directly to the promoters of anthocyanin biosynthesis genes but regulates anthocyanin biosynthesis through the formation of the MBW complex with the MYB and bHLH transcription factors^22^.

Recent studies have clarified some aspects of anthocyanin transport in plants. Anthocyanins are biosynthesized on the cytosolic surface of the endoplasmic reticulum (ER) and then transported into the vacuole for storage. Several anthocyanin transporters have been isolated, including glutathione *S*-transferase (GST), the adenosine triphosphate (ATP)-binding cassette (ABC), and the multidrug and toxic compound extrusion (MATE) protein^7,23–26^. In this study, we focused on the GST family, which are multifunctional enzymes involved either in the accumulation of secondary metabolites or in the detoxification of diverse exogenous substrates via conjugation of glutathione to diverse electrophilic compounds^27^. The GST superfamily can be divided into the following 14 classes according to sequence relatedness, kinetic properties, genome organization, and immunological properties: phi (F), tau (U), lambda (L), dehydroascorbate reductase (DHAR), theta (T), zeta (Z), elongation factor 1Bγ (EF1Bγ), tetrachloro hydroquinone dehalogenase (TCHQD), glutathionyl hydroquinone reductase (GHR), iota, mPGES-2, Ure2p, hemerythrin, and metaxin^28,29^. Of these classes, GSTF, GSTU, GSTL, and DHAR are plant-specific classes, and the GSTUs and GSTFs are the most abundant^28,30,31^.

Previous studies have shown that the messenger RNA (mRNA) levels of genes encoding GSTs are regulated by various biotic and abiotic stresses, including drought, dehydration, wounding, hydrogen peroxide (H_2_O_2_), pathogen attack, and heavy metals^32^. Meanwhile, hormones such as auxins, ethylene, abscisic acid (ABA), 6-benzylaminopurine (6-BA), salicylic acid (SA), and methyl jasmonate (MeJA) can also affect GST transcription^33,34^.

In plants, GSTs are also involved in many endogenous biological processes. For example, as flavonoid-binding proteins, GSTs play important roles in flavonoid accumulation. Most of these GSTs are anthocyanin transporters, such as Bronze-2 (encoded by *bz2*) in maize, Anthocyanin 9 (encoded by *an9*) in petunia, Flavonoid-3 (encoded by *fl3*) in carnation, Transparent Testa 19 (encoded by *tt19*) in *Arabidopsis thaliana*, and Reduced Anthocyanin in Petioles (encoded by *rap*) in strawberry^7,24,35–38^. *Arabidopsis thaliana* TT19 has been confirmed as a carrier protein that transports anthocyanin from the cytosol to the vacuole^24,38,39^. In fruit crops, GSTs involved in anthocyanin transport include LcGST4 in litchi, MdGST in apple, Riant in peach, and RAP in strawberry^2,7,26,40,41^. The overexpression of *PAP1*, which encodes an MYB transcription factor, increased the transcript level of *TT19* in *Arabidopsis*^42^. Genetic approaches in strawberry demonstrated that *RAP* functions downstream of the transcription factor *FvMYB10*^7^. In apple, MdGST (MDP0000252292) appeared to function downstream of MdMYB10^2^; however, this observation has not been confirmed with additional supporting evidence.

In this study, we identified 23 *GST* genes in apple and found that *MdGSTF6* showed very significant expression in transcript levels compared with those of other *GST*s in ‘Yanfu 8’ apple. Subcellular localization proved that MdGSTF6 was located on the vacuolar membrane. The overexpression of *MdGSTF6* in the *A. thaliana tt19* mutant complemented the defective anthocyanin pigmentation, which suggested that MdGSTF6 was a transporter of anthocyanin. Silencing of *MdGSTF6* affected anthocyanin accumulation in apple fruit. Knockdown of *MdGSTF6* by RNA interference (RNAi) in cultured ‘Gala’ decreased anthocyanin accumulation in the leaves and petiole bases of seedlings. Biochemical experiments showed that MdMYB1 can bind directly to the *MdGSTF6* promoter in vivo and in vitro and then activate the expression of MdGSTF6. These results showed that *MdGSTF6* encodes an important anthocyanin transporter that affects anthocyanin accumulation in apples, and these findings will improve our understanding of the regulation of anthocyanin transport in apples.

## Results

### Identification and analysis of *GST*s in apple

**Fig. 1:**
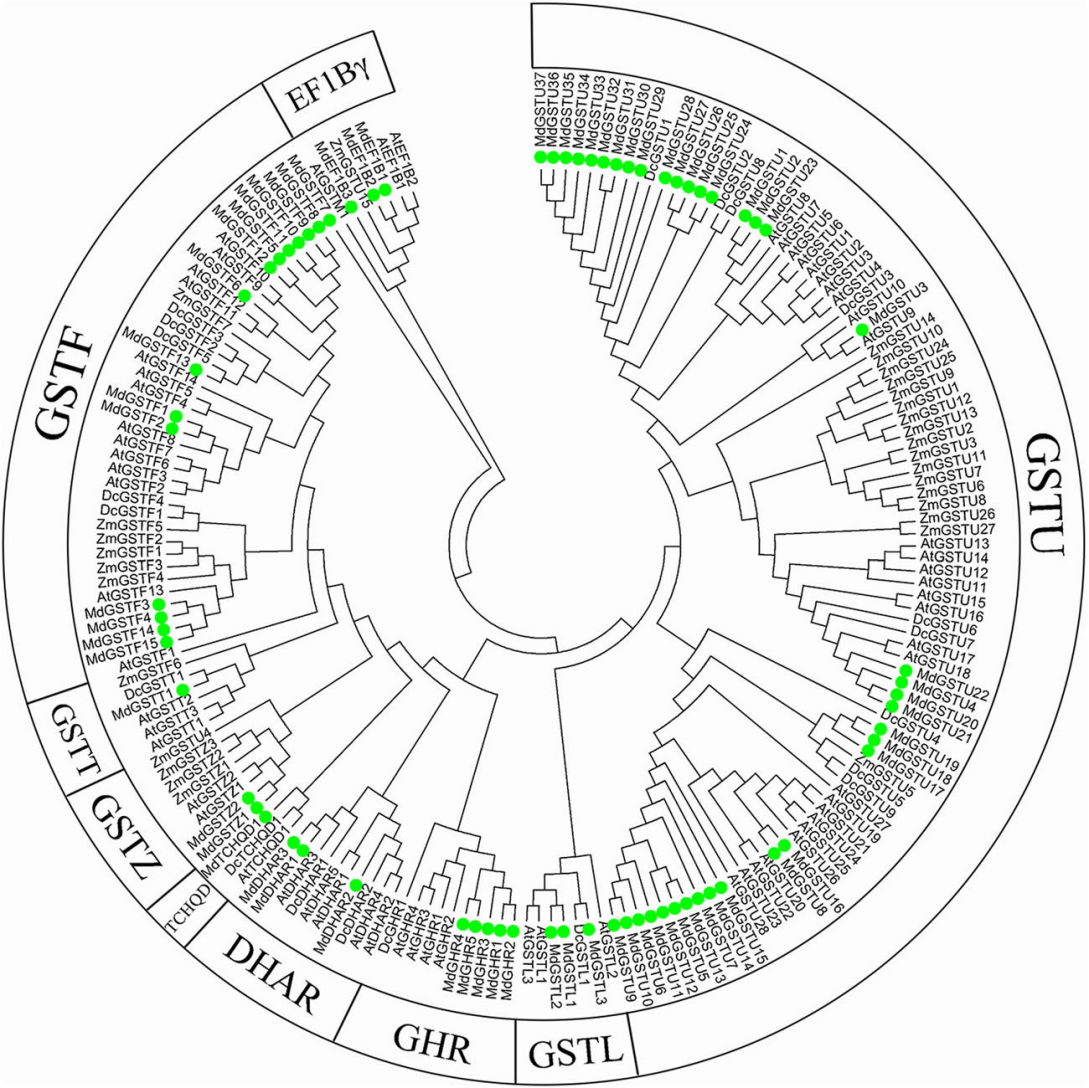
Phylogenetic tree of glutathione *S*-transferase (GST) proteins in apple, *Arabidopsis thaliana*, maize, and dragon’s blood tree (*Dracaena draco*). The 69 apple, 63 *A. thaliana*, 29 maize, and 20 *D. draco* GST protein sequences were aligned using Clustal W. The phylogenetic tree was constructed based on the neighbor-joining algorithm. GST proteins were clustered into nine classes. The protein sequences are listed in Table S3

We screened for GST genes in the new apple genome to identify and analyze apple *GST* genes^43^. A total of 69 GST-encoding genes were identified in the apple genome. To study the molecular evolution of *MdGST* genes and predict their functions, we conducted a phylogenetic analysis comparing GST proteins from apple, *A. thaliana*, maize, and dragon’s blood tree (*Dracaena draco*). The apple *GST* genes were divided into nine classes (F, U, L, Z, T, GHR, EF1Bγ, TCHQD, and DHAR) based on previous reports. Among these, classes F and U were the largest, with 15 and 37 *GST* genes, respectively (Fig. 1). These results were similar to those reported elsewhere^28,30,31^.

### *MdGST* transcript levels during fruit coloration

**Fig. 2:**
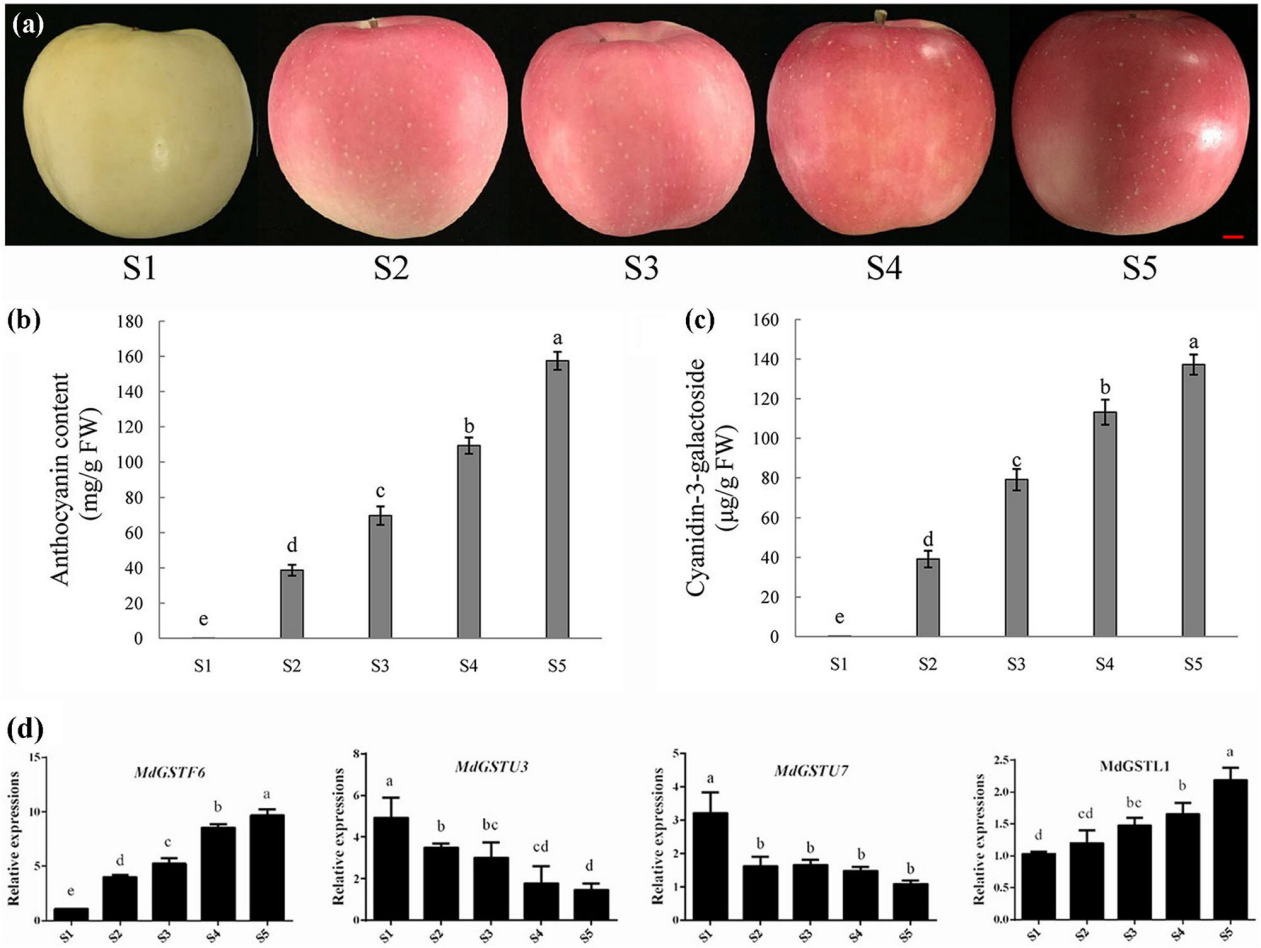
Fruit development and anthocyanin accumulation in ‘Yanfu 8’ apple cultivars. **a** Fruit development of 'Yanfu 8’ at the indicated time points. **b** Anthocyanin contents in fruit peels of ‘Yanfu 8’. **c** Cyanidin-3-galactoside contents in fruit peels of ‘Yanfu 8’. Scale bar: 1 cm. **d** Expression of 4 glutathione *S*-transferase (GST) genes during anthocyanin accumulation in apple. Expression levels were calculated relative to apple actin. Three biological replicates of each sample were analyzed. Data are expressed as the means ± SD, *n* = 3. The different letters denote significant differences according to one-way analysis of variance (ANOVA) (*P* < 0.05)

In plants, GSTs are not only involved in various biotic and abiotic stress responses but also in many other endogenous biological processes. To study the relationship between GSTs and the anthocyanin pathway, we monitored *GST* transcript levels (23 of 69 *GST* genes) in the ‘Yanfu 8’ apple fruit peel at five stages (S1 to S5; Fig. 2a). The total anthocyanin content of ‘Yanfu 8’ apple fruit peels increased from S1 to S5 (Fig. 2b). The main anthocyanin in apple is cyanidin-3-galactoside^15^. Therefore, we quantified cyanidin-3-galactoside in five stages of ‘Yanfu 8’. The cyanidin-3-galactoside content in ‘Yanfu 8’ fruit skins increased from 0.26 to 137.2 μg/g during S1 to S5 (Fig. 2c).

To investigate the MdGSTs related to anthocyanin, we monitored the expression of 23 of 69 *MdGST* genes during the fruit-coloring stage of ‘Yanfu 8’ fruits with a quantitative real-time polymerase chain reaction (qRT-PCR) (Fig. 2c; Fig. S1). Of the 23 selected *GST* genes, 8 and 2 exhibited upregulated and downregulated expression, respectively, during the fruit-coloring stage. Meanwhile, five *GST* genes were highly expressed during S2 and S3 but subsequently exhibited downregulated expression. *MdDHAR3* showed no significant difference during coloration, and other GSTs showed different expression patterns. For example, the *MdGSTF6* transcript level increased from S1 to S5, and the *MdGSTL1* transcript level gradually increased from S1 to S5. Moreover, *MdGSTF6* transcription increased continuously during the apple fruit-coloring stage, with the transcript level in S5 being more than 9 times higher than that in S1. MdGSTF6 was most significantly expressed among the apple *GST* genes. A correlation analysis revealed a positive correlation between anthocyanin content and *MdGSTF6* expression (*r* = 0.949). These results suggested that MdGSTF6 probably plays important roles in the coloration of the apple fruit peel.

### Characterization of MdGSTF6 in apple

**Fig. 3:**
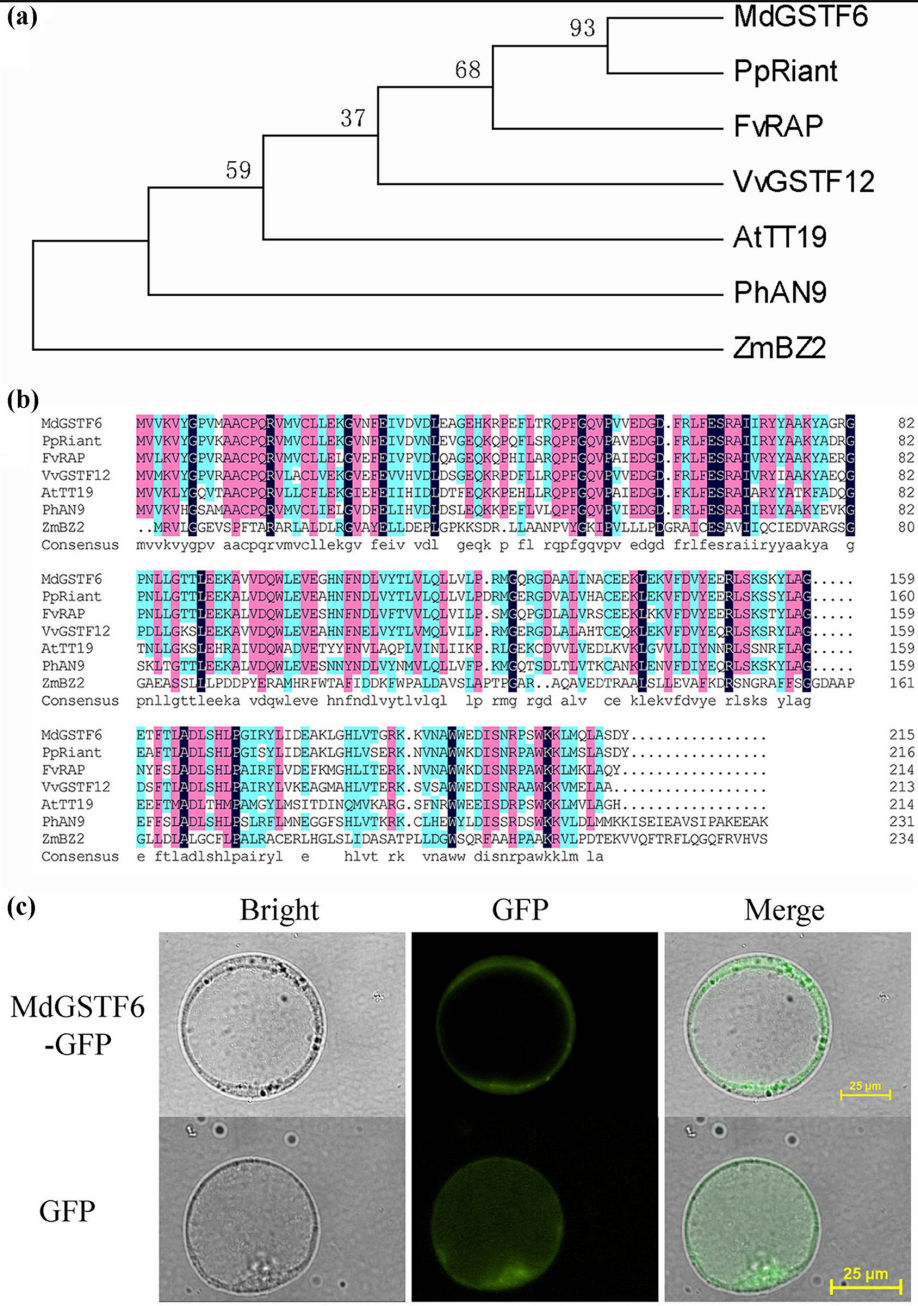
Phylogenetic analysis and subcellular localization of MdGSTF6. **a** Phylogenetic tree analysis of glutathione *S*-transferases (GSTs). The protein sequences are listed in Table S3. **b** Sequence alignment of seven GSTs. TT19 in *Arabidopsis thaliana* (NC_003076.8), Riant in peach (ALE31200.1), RAP in strawberry, GSTF12 in grape (NP_001267869.1), AN9 in petunia (CAA68993.1), and BZ2 in maize (NP_001183661.1) were used to build the phylogenetic tree and for sequence alignment analysis. **c** Subcellular localization of the 35S::MdGSTF6-GFP fusion construct in apple callus protoplasts. Protoplasts expressing 35S::GFP were used as a control

Protein expression has also been determined in ‘Yanfu 8’ apple skin. The protein expression of MdGSTF6 increased from S1 to S5 (Fig. S2). This result was similar to its gene expression. In the phylogenetic tree, *MdGSTF6* was homologous with *PpRiant, FvRAP*, and *VvGSTF12*, as well as *AtGST12* (also known as *TT19*) (Figs. 1 and 3a). The sequence alignment analyses of GSTs showed that the amino acid sequence of MdGSTF6 was similar to those of PpRiant, FvRAP, and VvGSTF12. The phylogenetic and sequence alignment analyses showed that *MdGSTF6* was closely related to *PpRiant*. Since *PpRiant* is known to encode an anthocyanin transporter in peach, *MdGSTF6* is a strong candidate for being an anthocyanin transporter involved in apple coloration.

### Subcellular localization of MdGSTF6

The MdGSTF6 in apple was a candidate anthocyanin transporter, suggesting that it might function in the vacuolar membrane. To validate this possibility, a 35S::*MdGSTF6*-GFP vector was constructed and introduced into apple calli, with 35S::GFP as the negative control. Protoplasts were isolated from the two transgenic calli, and the subcellular localization of the 35S::MdGSTF6-GFP fusion protein and the 35S::GFP protein was detected by fluorescence microscopy. The 35S::MdGSTF6-GFP was located on the vacuolar membrane, whereas 35S::GFP was distributed throughout the whole protoplast (Fig. 3c), indicating that MdGSTF6 is located on the vacuolar membrane.

To further confirm that MdGSTF6-GFP is located on the vacuolar membrane, we separated the membrane and cytoplasmic proteins from the 35S::*MdGSTF6*-GFP and 35S::GFP transgenic calli. A western blot indicated that the GFP signal was detected in the membrane protein of 35S::*MdGSTF6*-GFP callus instead of cytoplasmic protein (Fig. S3). Meanwhile, the green fluorescent protein (GFP) signal was detected in both the membrane protein and cytoplasmic protein in 35S::GFP callus (Fig. S3). These results confirmed that MdGSTF6 is located on the vacuolar membrane.

### Heterologous expression of *MdGSTF6* in the *tt19* mutant

**Fig. 4:**
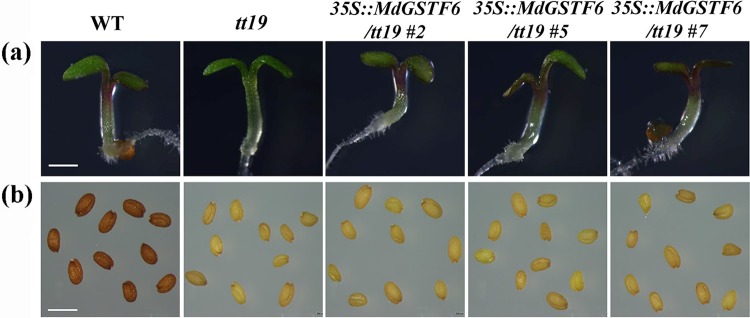
Phenotypes of 35S::MdGSTF6-GFP transgenic lines in *Arabidopsis thaliana tt19*. **a** Phenotypes of 7-day-old seedlings grown on Murashige and Skoog (MS) medium with 6% sucrose. Scale bar: 4 mm. **b** Images of *A. thaliana* seeds. Images of wild-type (WT), *tt19*, and three transgenic lines of 35S::MdGSTF6-GFP (lines 2, 5, and 7) in a *tt19* background are presented. Scale bar: 1 mm

*MdGSTF6* is a homolog of *A. thaliana* TT19, which encodes an anthocyanin transporter^24^. To test the function of MdGSTF6 in anthocyanin transport, we transformed 35S::*MdGSTF6*-GFP into the *Arabidopsis tt19* mutant. Approximately 20 transgenic lines were obtained with similar phenotypes, and 3 of them (Line 2, Line 5 and Line 7) were confirmed to have high transcript levels of *MdGSTF6* by qRT-PCR (Fig. S4). Thus, these three lines were used for further analyses. The seeds of the wild-type (WT), *tt19*, and the three 35S::*MdGSTF6*-GFP/*tt19* lines were germinated on Murashige and Skoog (MS) medium containing 6% sucrose. At 7 days after germination, the hypocotyls of the *tt19* seedlings remained green, while those of the WT and three 35S::*MdGSTF6*-GFP/tt19 seedlings were red (Fig. 4a). The transgenic lines accumulated more anthocyanin than the *tt19* line on medium containing 6% sucrose (Fig. S5). The brown color of seed coats was not rescued in the 35S::*MdGSTF6*-GFP transgenic lines (Fig. 4b). This suggested that MdGSTF6, such as petunia AN9 and peach Riant, complements anthocyanin pigmentation in vegetative tissues, but not in the seed coat^38,41^. The ability of MdGSTF6 to complement the *tt19* mutant demonstrated that MdGSTF6 is involved in anthocyanin transport.

### Virus-induced gene silencing of MdGSTF6 influences apple fruit coloration

**Fig. 5:**
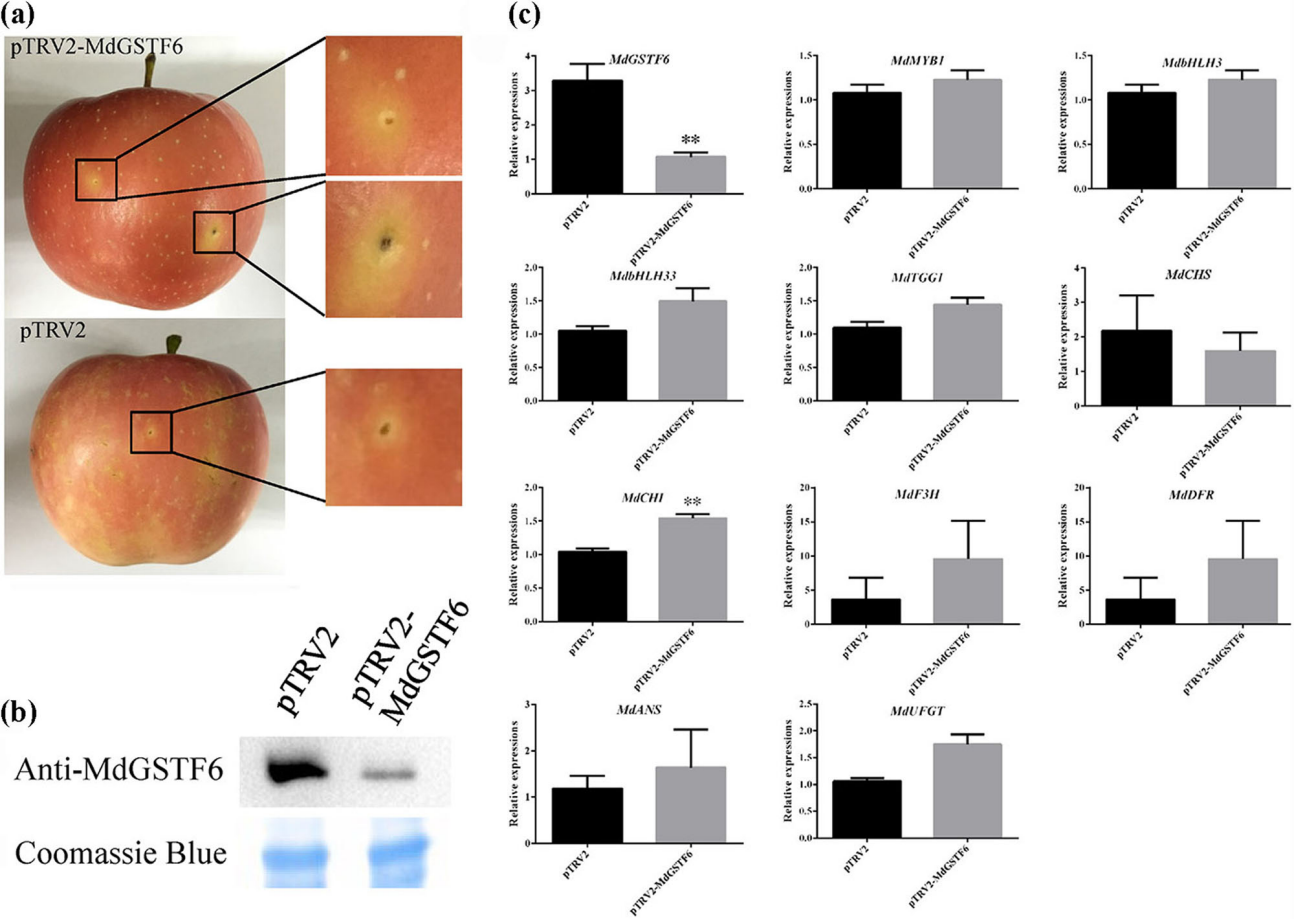
*MdGSTF6* silencing inhibits anthocyanin accumulation in apple peels. **a** Phenotypes of ‘Fuji’ fruits after silencing *MdGSTF6*. Empty pTRV2 was used as a control. **b** Abundance of MdGSTF6 protein in *MdGSTF6-*silenced apple and control **c** Transcript levels of genes in anthocyanin pathway in MdGSTF6-silenced apple and control. CHS chalcone synthase, CHI chalcone isomerase, F3H flavanone 3-hydroxylase, DFR dihydroflavonol 4-reductase, ANS anthocyanidin synthase, UFGT UDP-glucose: flavonoid-3-*O*-glucosyltransferase, GST glutathione *S*-transferase MdGSTF6. Data are expressed as the means ± SD, *n* = 3. The asterisks denote significant differences according to one-way analysis of variance (ANOVA) (**P < 0.01)

To demonstrate whether MdGSTF6 is essential for apple fruit coloration, we conducted transient expression analyses using the virus-induced gene silencing (VIGS) system. A specific complementary DNA (cDNA) fragment of *MdGSTF6* was introduced into the pTRV2 vector to produce pTRV2-MdGSTF6. pTRV2 was used as the control. Both vectors were infiltrated into apple fruit along with pTRV1. The injection sites on the apple peel remained pale after transformation with pTRV1 and pTRV2-*MdGSTF6*, but no clear phenotype appeared after the transformation with the control vector (Fig. 5a). Additionally, the anthocyanin (Fig. S6A) and cyanidin-3-galactoside (Fig. S6B) contents as well as the abundance of MdGSTF6 protein (Fig. 5b) were lower in pTRV2-*MdGSTF6* apple peels than in pTRV2 apple peels. Furthermore, knockdown of *MdGSTF6* strongly downregulated the expression of *MdGSTF6* but did not affect the transcription of the other genes in the anthocyanin biosynthesis pathway (Fig. 5c). Together, these results showed that knockdown of *MdGSTF6* affects anthocyanin accumulation, which indicates that *MdGSTF6* plays an essential role in anthocyanin accumulation in apple fruit.

### MdGSTF6-RNAi decreases anthocyanin accumulation in transgenic ‘Gala’ seedlings

**Fig. 6:**
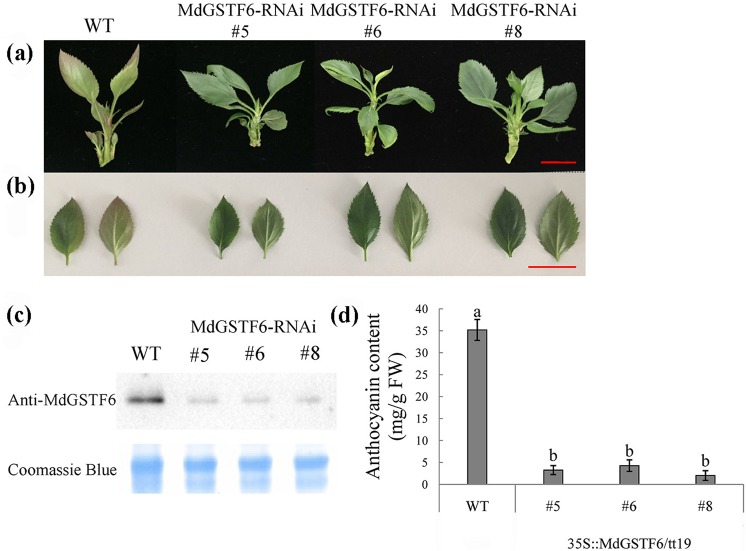
MdGSTF6 silencing inhibits anthocyanin accumulation in ‘Royal Gala’ transgenic lines. **a** Phenotype of wild-type (WT) and three MdGSTF6-RNAi lines (#5, #6, and #8) in response to sucrose treatment (6%). **b** Leaves of WT and three transgenic ‘Royal Gala’ lines under sucrose treatment. **c** Abundance of MdGSTF6 protein in WT and three transgenic ‘Royal Gala’ lines. Plant total proteins were visualized with Coomassie brilliant blue staining. **d** Anthocyanin contents in WT and three transgenic ‘Royal Gala’ lines. Data are expressed as the means ± SD, *n* = 3. The different letters denote significant differences according to one-way analysis of variance (ANOVA) (*P* < 0.05)

To further confirm that MdGSTF6 is essential for apple fruit coloration, RNAi was performed to knock down *MdGSTF6* in ‘Gala’ seedlings. In apple, anthocyanin levels increase in response to sucrose^44^. Therefore, we treated WT ‘Gala’ and *MdGSTF6*-RNAi ‘Gala’ lines with 6% sucrose. The WT ‘Gala’ seedlings accumulated anthocyanin in leaves and petiole bases, whereas the three MdGSTF6-RNAi ‘Gala’ lines did not (Fig. 6a, b). The abundance of the MdGSTF6 protein (Fig. 6c) as well as the anthocyanin (Fig. 6d) and cyanidin-3-galactoside (Fig. S7) contents were lower in the MdGSTF6-RNAi ‘Gala’ lines than in the WT ‘Gala’ control. The transcript levels of genes involved in anthocyanin biosynthesis were detected by qRT-PCR. The transcript levels of the structural genes *MdDFR*, *MdANS*, and *MdUFGT* were almost the same between WT and MdGSTF6-RNAi ‘Gala’ lines, while those of *MdCHS*, Md*CHI*, and *MdF3H* were slightly higher in WT than in MdGSTF6-RNAi ‘Gala’. The transcript level of *MdGSTF6* was much lower in the MdGSTF6-RNAi ‘Gala’ lines than in the WT ‘Gala’ control (Fig. S8A). The mRNA levels of the regulatory genes *MdMYB1* and *MdbHLH3/33* did not differ significantly between the WT ‘Gala’ and MdGSTF6-RNAi ‘Gala’ lines (Fig. S8B). These results suggest that knockdown of *MdGSTF6* does not influence the expression of most of the anthocyanin structural and regulatory genes. Similar results have been reported for the *A. thaliana tt19* mutant and the strawberry *rap* mutant^7,24^. These results further demonstrated that MdGSTF6 plays an important role in anthocyanin accumulation in apples.

### Analysis of the *MdGSTF6* promoter and the associated transcriptional regulation

To explore the region upstream of *MdGSTF6*, the *MdGSTF6* promoter was cloned, and its sequence was analyzed using the PlantCARE online tools. We detected several hormone response elements in the promoter, including MeJA, auxin, and ABA response elements, as well as heat- and low-temperature response elements (Table S1). These results suggested that the expression of *MdGSTF6* might be affected by hormones and temperature. In addition, a MYB-binding site was located at −1151 bp from the transcriptional start site (Table S1).

**Fig. 7:**
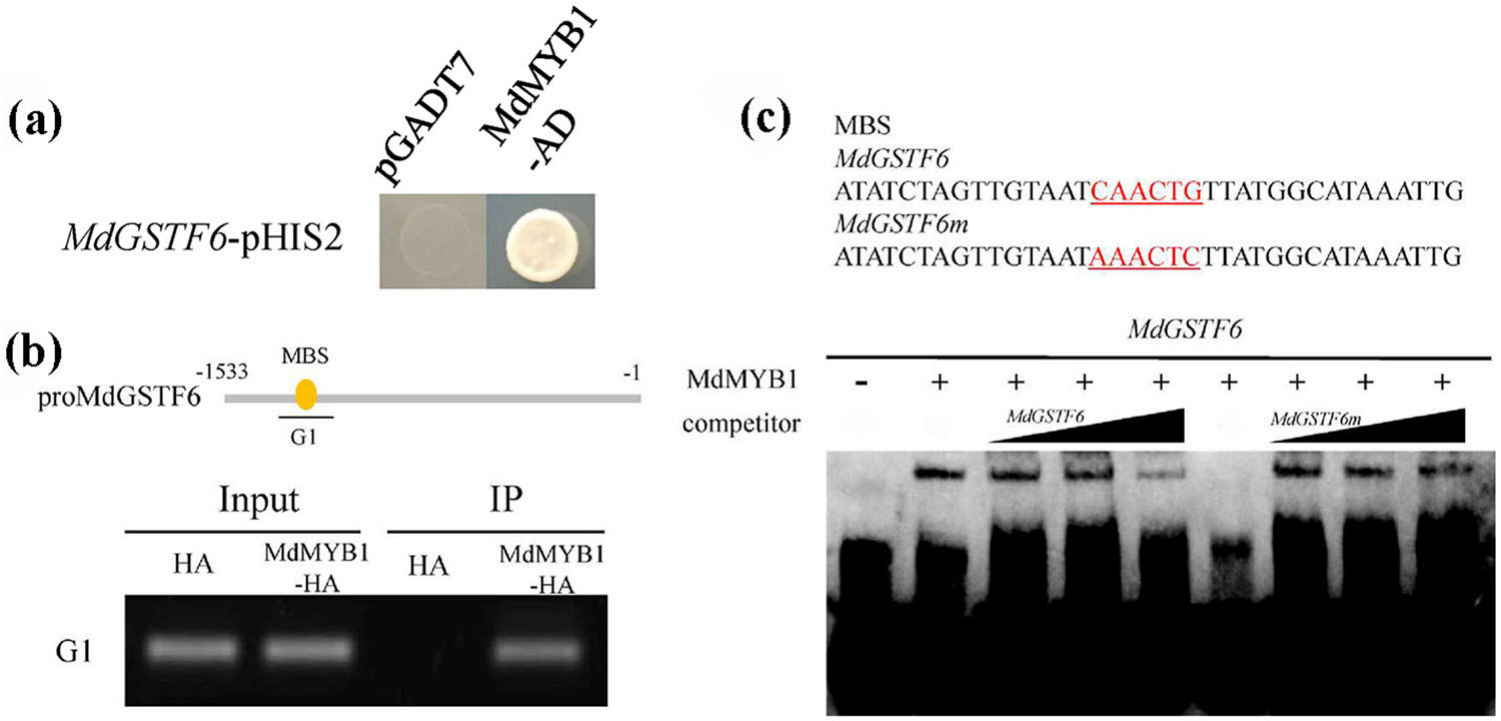
MdMYB1 bound to the *MdGSTF6* promoter. **a** Y1H assay showing the interaction between MdMYB1 and the MdGSTF6 promoter. The empty pGADT7 vector was used as a control. **b** Chromatin immunoprecipitation (ChIP) assay showing binding of MdMYB1 to the G1 region of the MdGSTF6 promoter in vivo. Apple ‘Orin’ calli overexpressing HA protein was used as a control. **c** Electrophoretic mobility shift assay (EMSA) result showing the interaction between MdMYB1 and labeled probes in the *MdGSTF6* promoter. Red letters indicate MYB-binding site (MBS) and mutated MBS (MdGSTF6m). Lane 2 contains labeled DNA probes and MdMYB1 protein without a competitor. Increasing amounts (10×, 25×, and 50×) of unlabeled normal DNA probes (MdGSTF6 in lanes 3, 4, and 5 of each blot) or unlabeled mutated DNA probes (MdGSTF6m in lanes 7, 8, and 9 of each blot) were added as cold competitors. Lane 6 contains labeled mutated DNA probes and the MdMYB1 protein

To explore upstream of MdGSTF6, a Y1H screening was performed from the apple fruit cDNA library. *MdGSTF6*-p-pHIS2 vectors were first grown on −Trp/−His medium containing 3-amino-1,2,4-triazole (3-AT) for screening. The concentration of 3-AT used was 100 mM. Four putative MdGSTF6-interacting candidates were obtained. After sequencing, three putative proteins were identified, and all three originated from the same gene, *MdMYB1* (DQ886414.1), which is the master regulatory gene in the anthocyanin pathway in apple. To confirm this result, we conducted a Y1H assay using *MdMYB1*-pGADT7 and *MdGSTF6*-p-pHIS2 vectors. Y1H results showed that MdMYB1 can bind to the *MdGSTF6* promoter (Fig. 7a).

To validate this interaction in vivo, we conducted a chromatin immunoprecipitation (ChIP) assay using the 35S::*MdMYB1*-HA and 35S::HA transgenic apple calli generated in this study. We tried to amplify the G1 region of the *MdGSTF6* promoter from the two transgenic calli by semi-PCR, but we obtained a product only from 35S::*MdMYB1*-HA calli (Fig. 7b). This result suggested that MdMYB1 can bind to the *MdGSTF6* promoter in vivo. An electrophoretic mobility shift assay (EMSA) was also performed, and the results also showed that MdMYB1 can specifically bind to the *MdGSTF6* promoter (Fig. 7c).

**Fig. 8:**
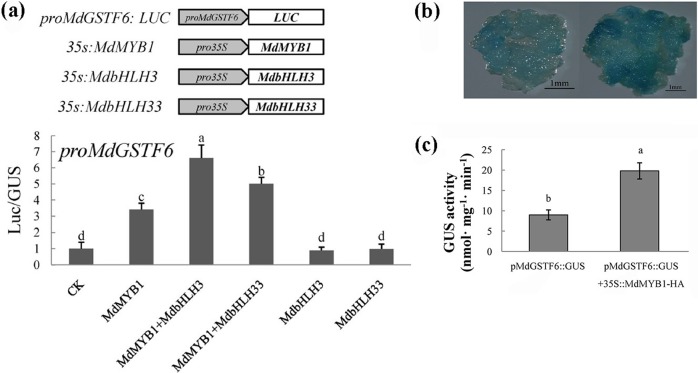
MdMYB1 activates transcription of *MdGSTF6* to enhance its expression. **a** Effects of MdMYB1, MdbHLH3, and MdbHLH33 individually and in combination on the promoter activity of *MdGSTF6* in a luciferase reporter assay. **b** β-Glucuronidase (GUS) staining of pMdGSTF6::GUS and pMdGSTF6::GUS plus 35S::MdMYB1-HA. **c** GUS activity in apple calli as labeled. GUS activity was measured three times with three replicates

To explore whether MdMYB1 affects the transcriptional activity of the *MdGSTF6* promoter, we conducted luciferase (LUC) and β-glucuronidase (GUS) assays. For the LUC assay, we constructed the *pMdGSTF6*-LUC reporter and three effectors: 35S:*MYB1*, 35S:*MdbHLH3*, and 35:*MdbHLH33* (Fig. 8a). Relative to the control expression level, MdGSTF6 expression was upregulated threefold by transient expression of MdMYB1. Compared with MdGSTF6 expression with the effector MdMYB1, MdGSTF6 expression was further upregulated by MdMYB1+MdbHLH3 and MdMYB1+MdbHLH33. However, MdbHLH3 and MdbHLH33 alone did not affect *MdGSTF6* expression (Fig. 8a). For the GUS assay, *pMdGSTF6*::GUS was constructed and transformed into 35S::*MdMYB1*-HA transgenic calli. After GUS staining, the cotransformed transgenic calli were darker than those harboring *pMdGSTF6*::GUS alone (Fig. 8b). Consistent with the GUS staining results, the GUS activity was much higher in the cotransgenic calli than in the calli harboring only *pMdGSTF6*::GUS (Fig. 8c). These results confirmed that MdMYB1 is able to activate *MdGSTF6* expression.

## Discussion

Fruit color is one of the most important agronomic traits of fruit quality and is an important attribute in terms of consumer preference. Anthocyanins in fruit not only confer different bright colors but are also beneficial for human health. Therefore, there is considerable interest in regulating the anthocyanin biosynthesis pathway.

Previous studies have shown that GSTs play an important role in anthocyanin accumulation in maize, petunia, and *A. thaliana*, as their loss-of-function mutants show no anthocyanin accumulation^35,37,45^. The abundance of GSTs is reportedly correlated with fruit pigmentation in several horticultural crops, including grape, lychee, peach, and strawberry^7,26,41,46^. In strawberry fruit, genetic approaches have been used to explore the relationship between RAP and FvMYB10, which is a master regulatory transcription factor in anthocyanin biosynthesis. For example, the results showed that RAP is transcriptionally regulated by and functions downstream of FvMYB10^7^. However, the transcriptional regulatory mechanism of GSTs in fruit trees remains unclear. In this study, we isolated and identified a *GST* gene, *MdGSTF6*, which is highly expressed in colored apple fruits and is positively correlated with anthocyanin content. Our analyses of its transcriptional regulatory mechanisms provide new insights into anthocyanin transport in horticultural crops.

*GSTs* are a large and ancient gene family, and many GSTs have been identified in several plants. For example, 53 GSTs have been detected in *A. thaliana*^46^, as well as 79 in rice^47^ and 90 in tomato^48^. The GSTs can be clustered into 14 classes, 9 of which were found in apple: F, U, L, Z, T, GHR, EF1Bγ, TCHQD, and DHAR (Fig. 1). In *Malus*×*domestica*, MdGSTF6 belongs to the F subfamily and showed a positive correlation with anthocyanin content, suggesting that it may be the predominant type of GST in anthocyanin transport.

Subcellular localization analysis is an important method for understanding protein function. In *A. thaliana*, TT19 was first detected in the cytosol of immature seed coats and suspension-cultured cells^38^ and then on the tonoplast in cotyledons and hypocotyls^24^. In contrast, Bz2 in maize was found to be loosely associated with membranes^49^. In the current study, subcellular localization analysis showed that the MdGSTF6-GFP fusion protein was located on the vacuolar membrane. This location within the cell provided further evidence that MdGSTF6 is involved in transporting anthocyanin from the cytoplasm to the vacuole.

Anthocyanins are synthesized on the cytosolic surface of the ER and then transported into the vacuole, where they accumulate. There is increasing evidence that GSTs are essential for transporting anthocyanins from the ER into the vacuole^24,35,36,50–52^. In this study, the transgenic *tt19* lines with *MdGSTF6* rescued the anthocyanin phenotype, showing that MdGSTF6 was an anthocyanin transporter. When we knocked down *MdGSTF6* in apple peels via VIGS, the fruit did not accumulate anthocyanins (Fig. 5a). We also knocked down *MdGSTF6* in the shoots of cultured ‘Gala’ seedlings and found that anthocyanins did not accumulate in the leaves or the petiole base (Fig. 6). Not surprisingly, the RNAi of *MdGSTF6* in seedlings did not influence the expression of genes involved in anthocyanin biosynthesis. Similar results were reported for the *A. thaliana tt19* mutant and the strawberry *rap* mutant^7,24^. Together, our results demonstrated that *MdGSTF6* encoding GST is essential for anthocyanin accumulation in apples.

In apple, MdMYB1 and MdMYBA regulate anthocyanin biosynthesis in fruit peel. Additionally, MdMYB1 activates the transcription of *MdDFR* and *MdUFGT*, while MdMYBA binds to the *MdANS* promoter^18,19^. MdMYB10 was shown to be responsible for apple flesh color in red-fleshed apples; its mechanism of action is to interact with MdbHLH3/33 and increase the transcription of *MdDFR*^20^. These studies indicated that anthocyanin biosynthesis is controlled by the master regulatory transcription factor MdMYB1/A/10. Overexpression of the gene encoding the MYB transcription factor PAP1 in *A. thaliana* led to an increase in TT19 expression level^53^. *AtPAP1* is homologous to *MdMYB1*, which encodes a key regulator of the anthocyanin pathway^18^. In strawberry, RAP encodes a GST and acts downstream of the fruit-specific transcription factor FvMYB10^7^. The results of these studies suggested that MYB transcription factors may function upstream of GSTs in plants. In this study, through Y1H screening, we found that MdMYB1 can bind to the *MdGSTF6* promoter. Further study indicated that MdMYB1 can directly bind to the *MdGSTF6* promoter in vivo and in vitro (Fig. 7) and activate its transcription (Fig. 8). These findings provide a new understanding that demonstrated that MdMYB1 regulates anthocyanin biosynthesis as well as transport.

**Fig. 9:**
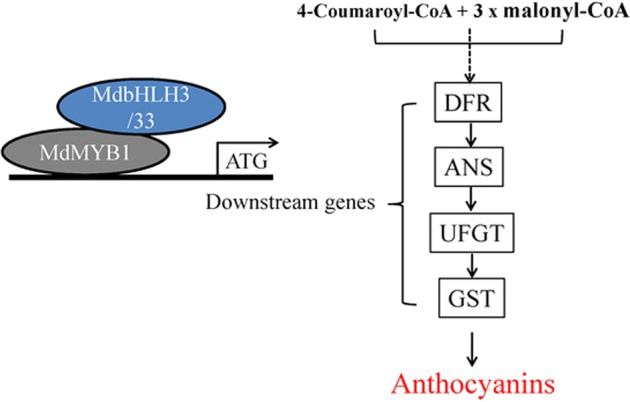
Model for the transcriptional regulation of anthocyanin biosynthesis and transport in apple. DFR dihydroflavonol 4-reductase, ANS leucoanthocyanidin dioxygenase, UFGT UDP-glucose: flavonoid-3-*O*-glucosyltransferase, GST glutathione *S*-transferase

Anthocyanin accumulation in fruit is a key quality trait in apple, and a high anthocyanin content is an important target in apple breeding. The regulation of the anthocyanin biosynthesis pathway has been well studied, while there have been fewer studies on anthocyanin transport. Therefore, a more thorough understanding of anthocyanin transport, including how it is regulated, is important for clarifying the mechanism underlying apple fruit coloration. In this study, we found that a *GST* gene, *MdGSTF6*, is directly regulated by MdMYB1, which activates its expression; its transgenic experiment in *A. thaliana tt19* lines provided further evidence for its involvement in transporting anthocyanin. Knocking down *MdGSTF6* reduced anthocyanin accumulation in apple fruits and seedlings. Collectively, our results demonstrate that *MdGSTF6* encodes an important GST transporter of anthocyanins in apple fruit and provide evidence for its regulatory mechanisms. Furthermore, MdMYB1 not only regulates anthocyanin synthesis but also controls the transport of anthocyanin in apples (Fig. 9). This information may be useful for improving our understanding of the regulation of anthocyanin transport in apples.

## Materials and methods

### Plant materials and growth conditions

The apple cultivar ‘Yanfu 8’ was grown in Yantai, Shandong Province, China. Fruits were bagged on 15 May 2016 (at 30 days after full bloom (DAFB)), and the bags were removed at 164 DAFB. Bags were removed at the time that rapid anthocyanin accumulation occurred. Samples were collected during the following five stages: S1 (0 days after bag removal (DABR)), S2 (4 DABR), S3 (8 DABR), S4 (12 DABR), and S5 (16 DABR). We collected 12 fruits during each stage. Peels from these fruits were pared with a knife and then immediately frozen in liquid nitrogen and stored at −80 °C until use.

Tissue cultures of *Malus*×*domestica* cv. ‘Gala’ apple were used and regarded as the WT control. Transgenic ‘Gala’ apple plants with MdGSTF6-RNAi were grown on MS medium containing 0.2 mg/L indole-3-acetic acid (IAA) and 0.5 mg/L 6-BA at 25 °C under a 16 h light/8-h dark photoperiod. The tissue-cultured ‘Gala’ seedlings were subcultured at 25-day intervals. For the sucrose treatment, apple shoots were cultivated on MS medium supplemented with 0.2 mg/L IAA, 0.5 mg/L 6-BA, and 6% (w/v) sucrose.

Callus cells of ‘Orin’ were grown on MS medium containing 0.4 mg/L 6-BA and 1.5 mg/L 2,4-dichlorophenoxyacetic acid (2,4-D) at 25 °C in the dark. These calli were subcultured at 14-day intervals.

### Measurement of total anthocyanins

To measure the anthocyanin content, 0.5 g fruit peel was ground into a powder in liquid nitrogen. The powder was then mixed with 5 mL cold methanol with 0.1% HCl and kept at 4 °C for 24 h in the dark. The two-buffer system, utilizing KCl buffer, pH 1.0 (0.025 M) and NaAc buffer, pH 4.5 (0.4 M), was used to determine the anthocyanin content as described elsewhere^54^. A 1 mL aliquot (three replicates) of the fruit peel extract was transferred to a 10 mL centrifuge tube, after which 4 mL KCl buffer was added. Another 1 mL aliquot (three replicates) of the fruit peel extract was placed in a 10 mL centrifuge tube, after which 4 mL NaAc buffer was added. Both solutions were mixed and extracted for 15 min at 4 °C in darkness. The absorbance of the solution was measured with a UV-1600 spectrophotometer (Shimadzu, Kyoto, Japan) at 510 and 700 nm. The anthocyanin content was calculated with the following formula: OD = (A530 − A620) − 0.1 × (OD650 − OD620).

### UPLC analysis of anthocyanin in apple fruit peels

Approximately 0.3 g of tissue was ground into a powder in liquid nitrogen, added to 2 mL of methanol, and then kept at 4 °C for 6 h in the dark. The mixture was centrifuged at 6000 × *g* for 30 min at 4 °C. The supernatant was filtered through a 0.45 μm membrane (Millipore, Billerica, MA, USA) and then subjected to ultra performance liquid chromatography (UPLC) analysis with an Acquity UPLC (Waters, Manchester, UK). The mobile phase was acetonitrile (solvent A) and 1% methane acid (solvent B) at a flow rate of 0.3 ml/min. Separation was performed using an Acquity BEH C18 column, 5 μm, 2.1 × 100 mm column. The linear gradient of phase B was as follows: 0–0.1 min, 95%; 0.1–8 min, 95–85%; 8–12 min, 85–79%; 12–15 min, 79–40%; 15–17 min, 40–10%; 17–17.1 min, 10–95%; and 17.1–20 min, 95%. The ultraviolet–visible light detector wavelength was set at 510 nm to detect anthocyanins. Cyanidin-3-galactoside (Sigma, St Louis, MO, USA) was used as the authentic standard.

### Identification and phylogenetic analysis of GST genes in apple

To identify all apple GST genes, the GST-C domain from the Pfam database (Pfam number PF00043; http://pfam.xfam.org)^55^ was used as the probe for hidden Markov models to search genome files downloaded from the apple genome database (https://iris.angers.inra.fr/gddh13/index.html)^43^. The protein sequences of GSTs from *A. thaliana*, *D. draco*, and maize (*Zea mays L*.) were used to construct a phylogenetic tree using MEGA 5.1 software with a bootstrap value of 1000. The protein sequences are listed in Table S3.

### Isolation of RNA and qRT-PCR analysis

Total RNA was extracted from ‘Yanfu 8’ apple peels or ‘Gala’ seedlings using an RNAprep Pure Plant Kit (Tiangen, Beijing, China). First-strand cDNA was synthesized using the TransScript II One-Step gDNA Removal and cDNA Synthesis SuperMix Kit (TransGen). The primers used for RT-PCR were designed with Beacon Designer 7 and were synthesized by QingKe Biological Technology (Qingdao, China). The primers are listed in Supplementary Table 2. The RT-PCR reactions were performed using qPCR SuperMix (TransGen). Three biological and three technical replicates for each reaction were analyzed on a CFX96 instrument (Bio-Rad, Hercules, CA, USA). The reaction conditions were as follows: 94 °C for 30 s followed by 40 cycles of 94 °C for 5 s, 58 °C for 15 s, and 72 °C for 10 s. A melting curve was produced at the end of each run for each sample. Transcript levels were calculated using the cycle threshold (Ct) 2^-ΔΔCt^ method^56^.

### Protein extraction and western blotting

Total proteins were extracted from apple peels and seedlings using the Plant Protein Extraction Kit (CWBiotech, Beijing, China) according to the manufacturer’s instructions. Then, the protein concentration was measured using a Nanodrop 2000 instrument (Thermo Scientific, Waltham, MA, USA).

The anti-MdGSTF6 antibody was prepared by Abmart Co. Ltd. (Shanghai, China). The unique peptide sequence ‘VDLEAGEHKRPE’ was designed as an antigen and then injected into rabbits to obtain the antibody. Additionally, MdGSTF6 protein was quantified by protein gel blotting using the anti-MdGSTF6 antibody. Proteins were separated by 12% sodium dodecyl sulfate–polyacrylamide gel electrophoresis and then transferred to polyvinylidene difluoride membranes (Millipore). The membranes were incubated with MdGSTF6 primary antibodies and then with secondary antibodies (Abmart) before visualizing immunoreactive proteins using Immobilon Western Chemiluminescent horseradish peroxidase substrate (Millipore). A Coomassie blue-stained gel served as the loading control.

### Subcellular localization analyses

MdGSTF6 was cloned into the pRI101-AN vector (Takara, Dalian, China) to construct the 35S::MdGSTF6-GFP recombinant vector. The vector was then transformed into *Agrobacterium tumefaciens* LBA4404 competent cells. Transgenic calli of 35S::MdGSTF6-GFP were obtained as described by Wang et al.^57^, while 35S::GFP was used as a negative control. Protoplasts were isolated from the two transgenic calli as described previously^58^. Fluorescence was observed under a fluorescence microscope (Nikon Ni-E, Tokyo, Japan).

The membrane protein and cytoplasmic protein were separated using the Membrane Protein and Cytoplasmic Protein Extraction Kit (Beyotime, Shanghai, China) according to the manufacturer’s instructions. The extracted proteins were tested by protein gel blotting using the anti-GFP antibody. A Coomassie blue-stained gel served as the loading control.

### Overexpression of MdGSTF6 in *Arabidopsis* tt19 mutant

The *A. tumefaciens* LBA4404 line containing 35S::MdGSTF6-GFP was introduced into the *A. thaliana* mutant tt19 line. Seeds from the T1 generation transgenic plants were grown and selected on MS medium containing kanamycin. Seeds of the T3 generation were collected for later use.

### Virus-induced gene silencing of MdGSTF6 in apple

A 399-bp fragment of MdGSTF6 (244–642 bp) was amplified and cloned into the pTRV2 vector. pTRV2-MdGSTF6, pTRV2, and pTRV1 were respectively transformed into the GV3101 *A. tumefaciens* strain for the VIGS experiments. The primers are shown in Table S2. The *A. tumefaciens* GV3101 lines of pTRV2-MdGSTF6, pTRV2, and pTRV1 were incubated and resuspended to an OD600 of 0.8 in infiltration buffer containing 10 mM MgCl_2_, 10 mM MES, and 150 μM acetosyringone (AS). Suspensions were kept at room temperature for 2 h without shaking. The bagged fruits of ‘Yanfu 8’ were selected to inject the VIGS constructs. A total of 150 fruits were selected and divided into two groups. The pTRV2-MdGSTF6 mixture (pTRV1:pTRV2- MdGSTF6 = 1:1, v/v) and a control mixture (pTRV1:pTRV2 = 1:1, v/v) were prepared for injection, and 300 μL of suspension was injected vertically into each point. The infiltrated apples were kept in the dark overnight and then stored in a growth chamber under light. After 10 days, the apple peels were collected for protein and RNA extraction.

### Knockdown of MdGSTF6 in ‘Royal Gala’ seedlings

The same sequence of MdGSTF6 used in VIGS was cloned into the pFGC1008 vector (http://www.chromdb.org) using the restriction sites *Asc*I/*Swa*I and *Bam*HI/*Spe*I for the first and second cloning^59^. The recombinant vector was then transformed into *A. tumefaciens* LBA4404-competent cells. Cells of the *A. tumefaciens* LBA4404 line were incubated and resuspended to an OD600 of 0.6 in MS liquid medium with 10 μM AS. Suspensions were kept at 28 °C for 0.5 h without shaking. The leaves of 20-day-old ‘Gala’ seedlings were prepared for infection. The infected leaves were transferred to MS solid medium containing hygromycin and carbenicillin and then cultured until resistant roots grew. The resistant roots were identified and subcultured for later use.

### Yeast one-hybrid assay

The *MdGSTF6* promoter sequence (1515 bp) was inserted into the pHIS2 vector (Clontech, Palo Alto, CA, USA), and the coding sequence (CDS) of *MdMYB1* was cloned into the pGADT7 vector (Clontech). The primers used to amplify the CDS of MdMYB1 and promoter of MdGSTF6 are listed in Table S2. To determine the suitable concentration of 3-AT to suppress background histidine leakiness of the pHIS2 vector, the recombinant pHIS2 vectors transformed into the yeast strain Y187 were grown on −Trp/−His (−T/−H) medium containing different concentrations of 3-AT. The empty pGADT7 plasmid was used as the control.

### ChIP PCR analysis

The ChIP assay was performed using the anti-HA antibody (Abmart) and the EZ ChIP^TM^ Chromatin Immunoprecipitation Kit (Millipore) according to the manufacturer’s instructions. The resulting samples were analyzed by semiquantitative PCR using the primers listed in Table S2. Amplification products were visualized by agarose gel (1%) electrophoresis and used to evaluate protein binding.

The CDS of *MdMYB1* was cloned into the pCB302-HA vector to construct the 35S::*MdMYB1*-HA recombinant vector. The recombinant and empty vectors were then transformed into *A. tumefaciens* LBA4404 competent cells. Transgenic calli of 35S::MYB1-HA and 35S::HA were obtained as previously described^57^.

### Electrophoretic mobility shift assays

The EMSAs were performed using the LightShift Chemiluminescent EMSA Kit (Thermo Scientific). The CDS of *MdMYB1* was cloned into the expression vector pET-32a (EMD Biosciences, Novagen, San Diego, CA, USA). The MdMYB1 recombinant protein was expressed in *Escherichia coli* strain BL21 (Tiangen) and purified using a Ni-agarose His-Tagged Protein Purification Kit (CWbiotech). All probes were synthesized and labeled by Sangon Biotech Co. Ltd. (Shanghai, China). Double-stranded probes were synthesized using annealing buffer (Beyotime).

### Luciferase reporter assay

Protoplast transient expression was carried out as reported previously^58,60^. Briefly, UBQ10-GUS was cotransfected with FRK1-LUC as an internal control, and the promoter activity was determined by calculating the LUC/GUS ratio. Protoplasts were collected 6 h after the transfection for the promoter activity assays at room temperature. Protoplasts transfected with plasmid without effectors served as the control.

### GUS assay

The promoter of *MdGSTF6* (1515 bp) was cloned into the pBI121-GUS vector to construct pMdGSTF6::GUS, which was then transformed into 35S::MdMYB1-HA calli via an *A. tumefaciens*-mediated method to obtain cotransgenic calli. For histochemical staining, tissues were incubated in GUS staining buffer (1 mM 5-bromo-4-chloro-3-indolyl-β-D-GlcA (x-Gluc) in *N*,*N*-dimethylformamide, 0.1 mM EDTA, 0.5 M ferricyanide, 0.5 M ferrocyanide, and 0.1% Triton X-100) at 37 °C overnight. The GUS activity was measured as previously described^61^.

### Statistical analyses

Statistical analyses were performed using the SPSS 19.0 program (SPSS, Chicago, IL, USA). Variance and significant difference tests were carried out to identify differences among means by one-way analysis of variance (ANOVA) with Tukey’s HSD (honestly significant difference) method.

## Supplementary information

Supplementary figures and tables
