# Supplementary material for: MdGSTF6, activated by MdMYB1, plays an essential role in anthocyanin accumulation in apple
Source: Hortic Res. 2019 Mar 1;6:40. doi: 10.1038/s41438-019-0118-6 (PMC6395711; doi:10.1038/s41438-019-0118-6)
Supplement: Supplementary file 1 — Supplementary figures and tables [file 41438_2019_118_MOESM1_ESM.docx]

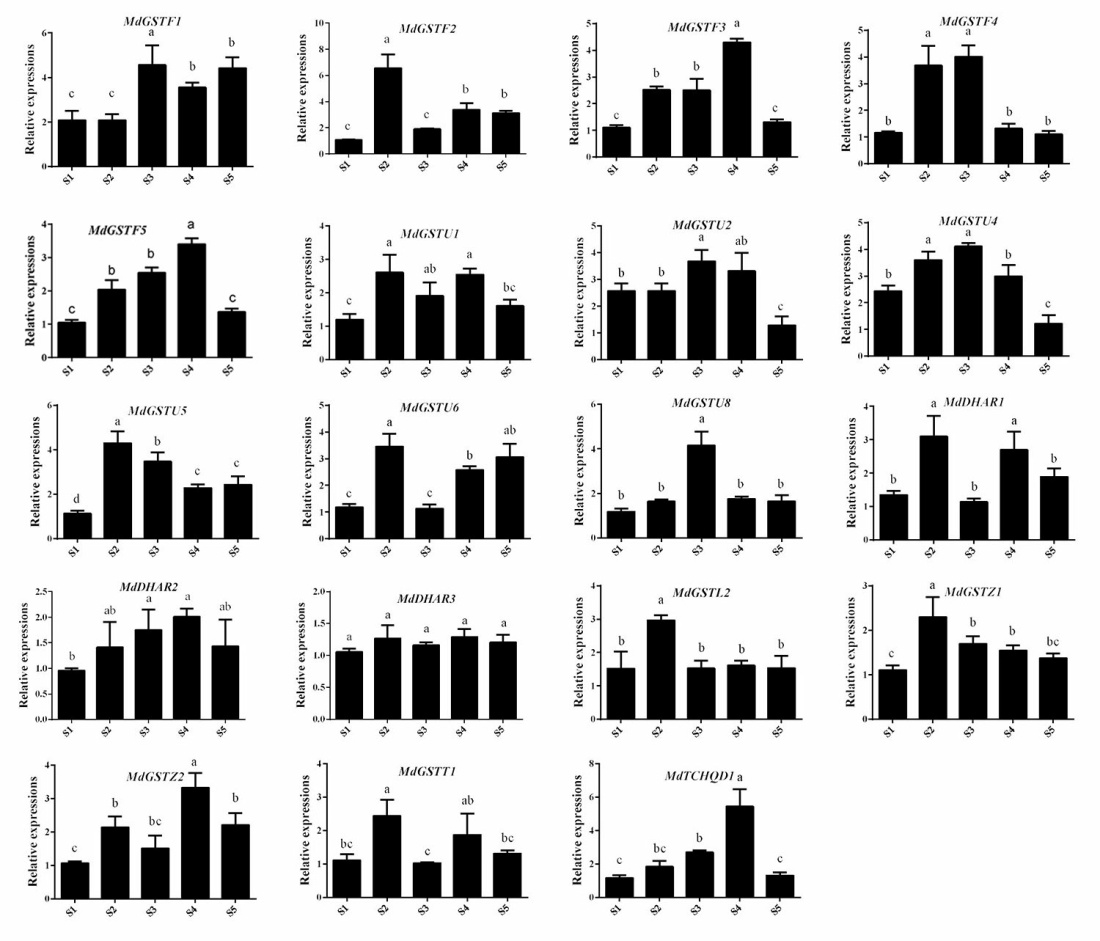


Fig. S1. Expression of 19 GST genes during anthocyanin accumulation in apple. Data are expressed as the means±SD, n = 3. The different letters denote significant differences according to one-way ANOVA test (P < 0.05).


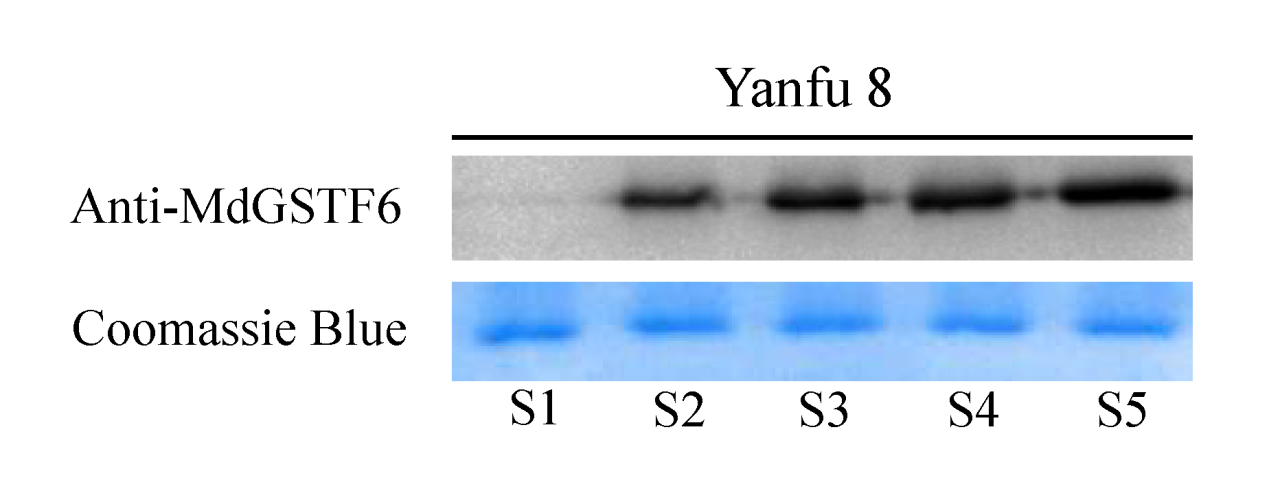
Fig. S2. The protein expression of MdGSTF6 in ‘Yanfu 8’. The total proteins were isolated from apple peels of ‘Yanfu 8’. The MdGSTF6 antibody was used for western blotting. Total proteins were visualized with coomassie blue staining. S1 to S5 indicates that samples were collected at five stages, they were S1 (0 DABR), S2 (4 DABR), S3 (8 DABR), S4 (12 DABR), and S5 (16 DABR).


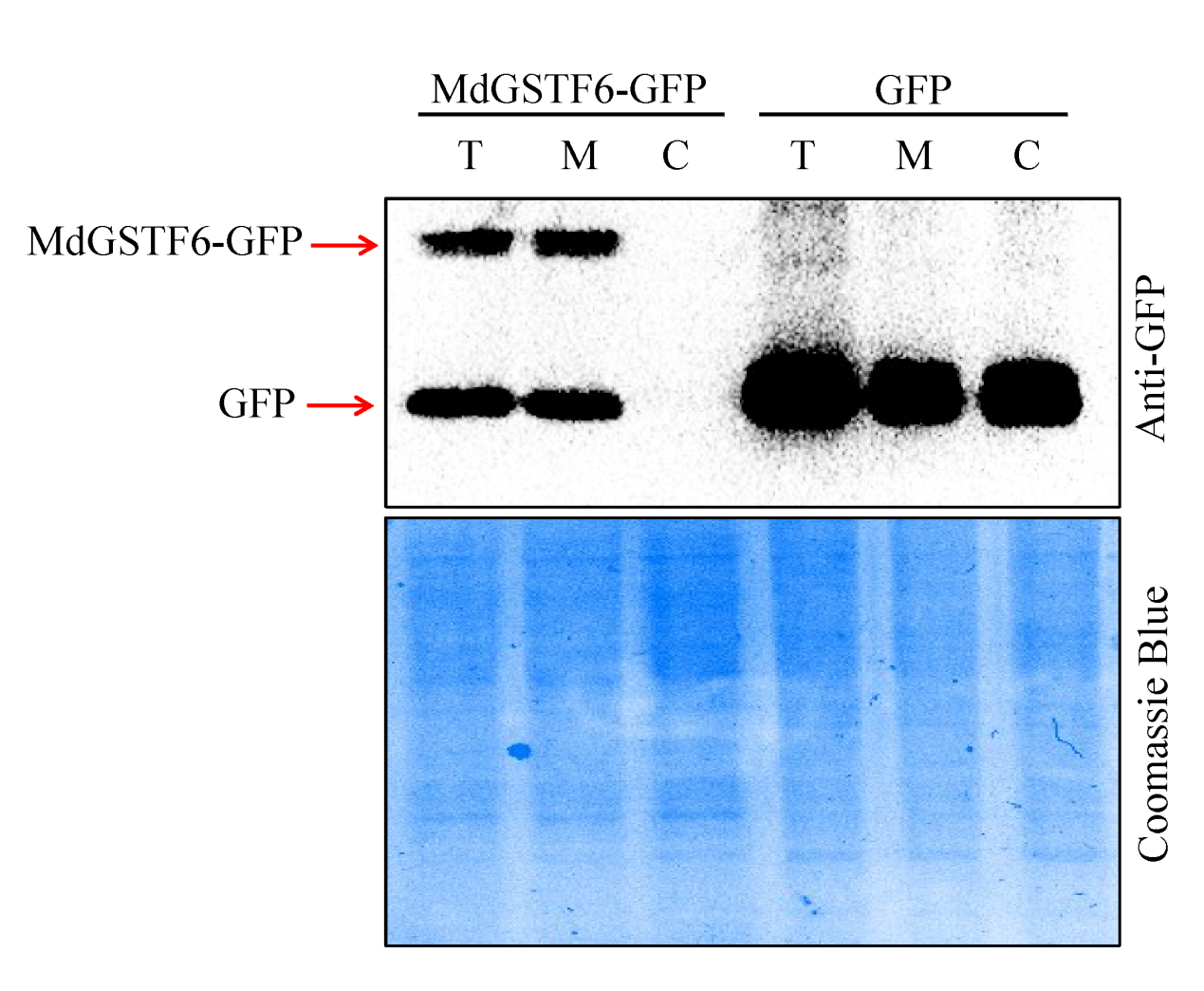


Fig. S3. Subcellular localization analyse of MdGSTF6 by Western blotting. The membrane and cytoplasmic proteins were separated from the 35S::MdGSTF6-GFP and 35S::GFP calli. The GFP antibody was used for western blotting and the coomassie blue was using as a loading control. T, M, C indicate total protein, membrane protein, cytoplasmic protein, respectively.


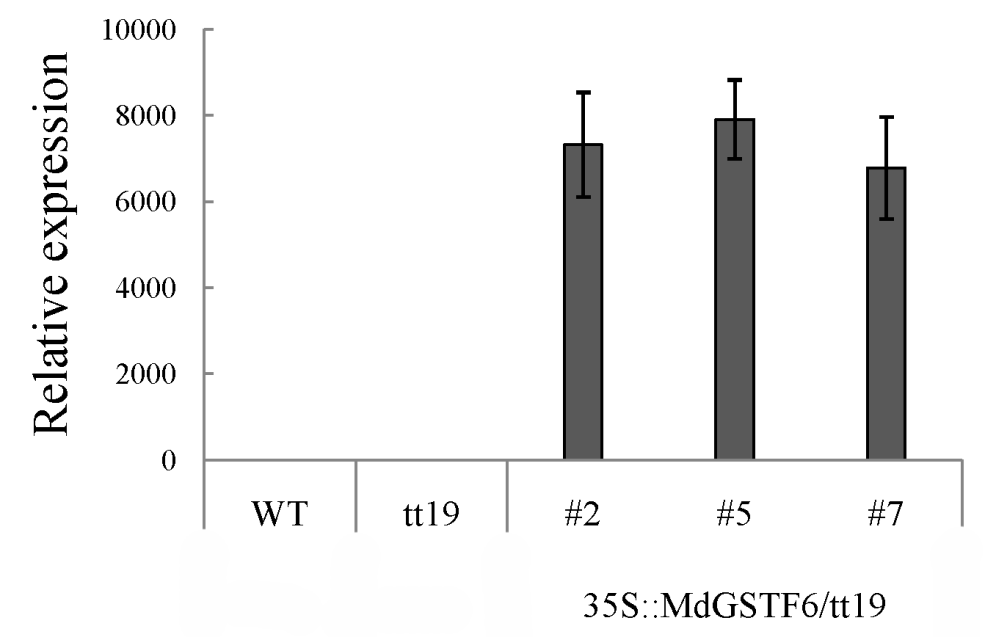


Fig. S4. Genotyping of the 35S::*MdGSTF6*-GFP transgenic lines in *Arabidopsis tt19*. Relative expression level of MdGSTF6 in seedlings of WT, *tt19*, and three transgenic lines of 35S::*MdGSTF6*-GFP (Line 2, Line 5, and Line 7) in the *tt19* background was examined by qRT-PCR. *Arabidopsis* actin (At3G18780) was used as the control. Data are means ± SD obtained from three technical repeats.


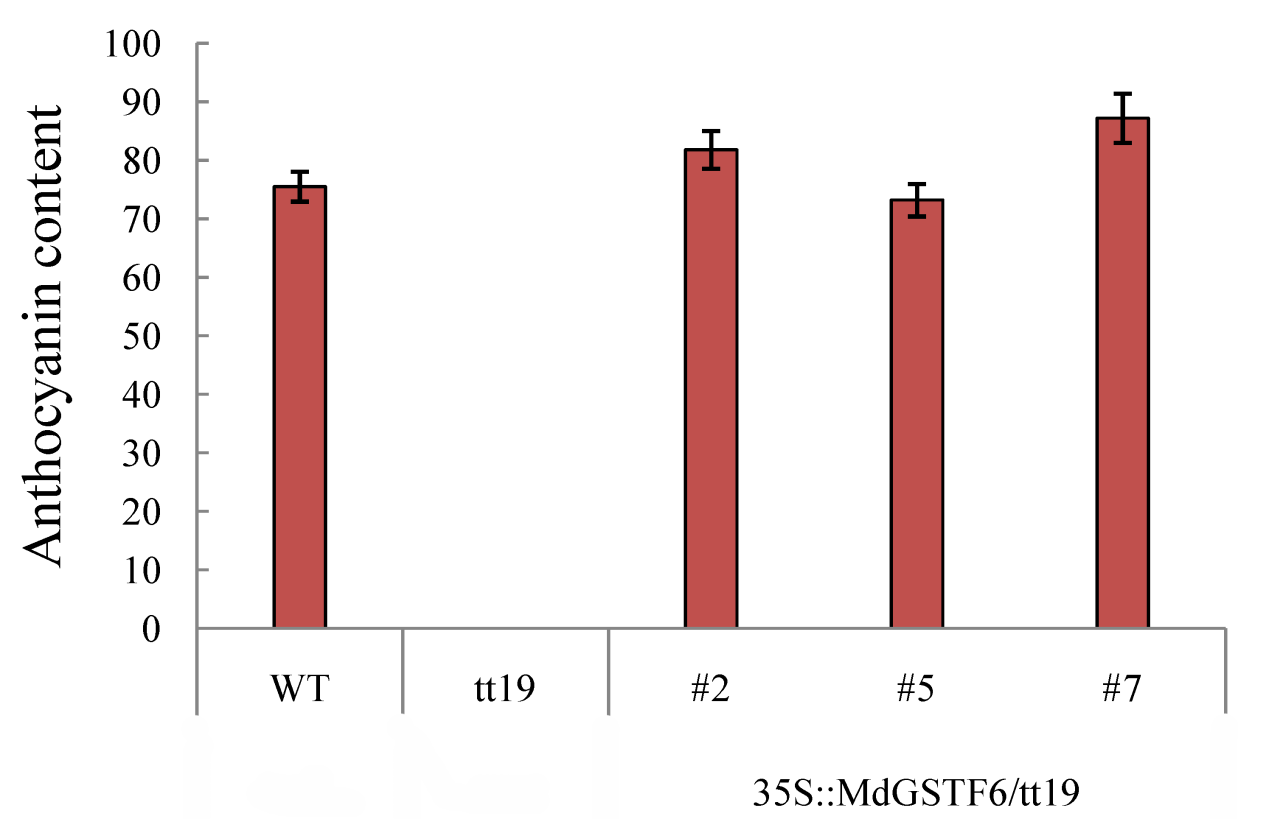


Fig. S5. The content of anthocyanin in WT, *tt19*, and three 35S::*MdGSTF6*-GFP transgenic lines in *Arabidopsis* tt19. The 10-day-old Arabidopsis seedlings were using for anthocyanin extraction. Data are means ± SD obtained from three technical repeats.


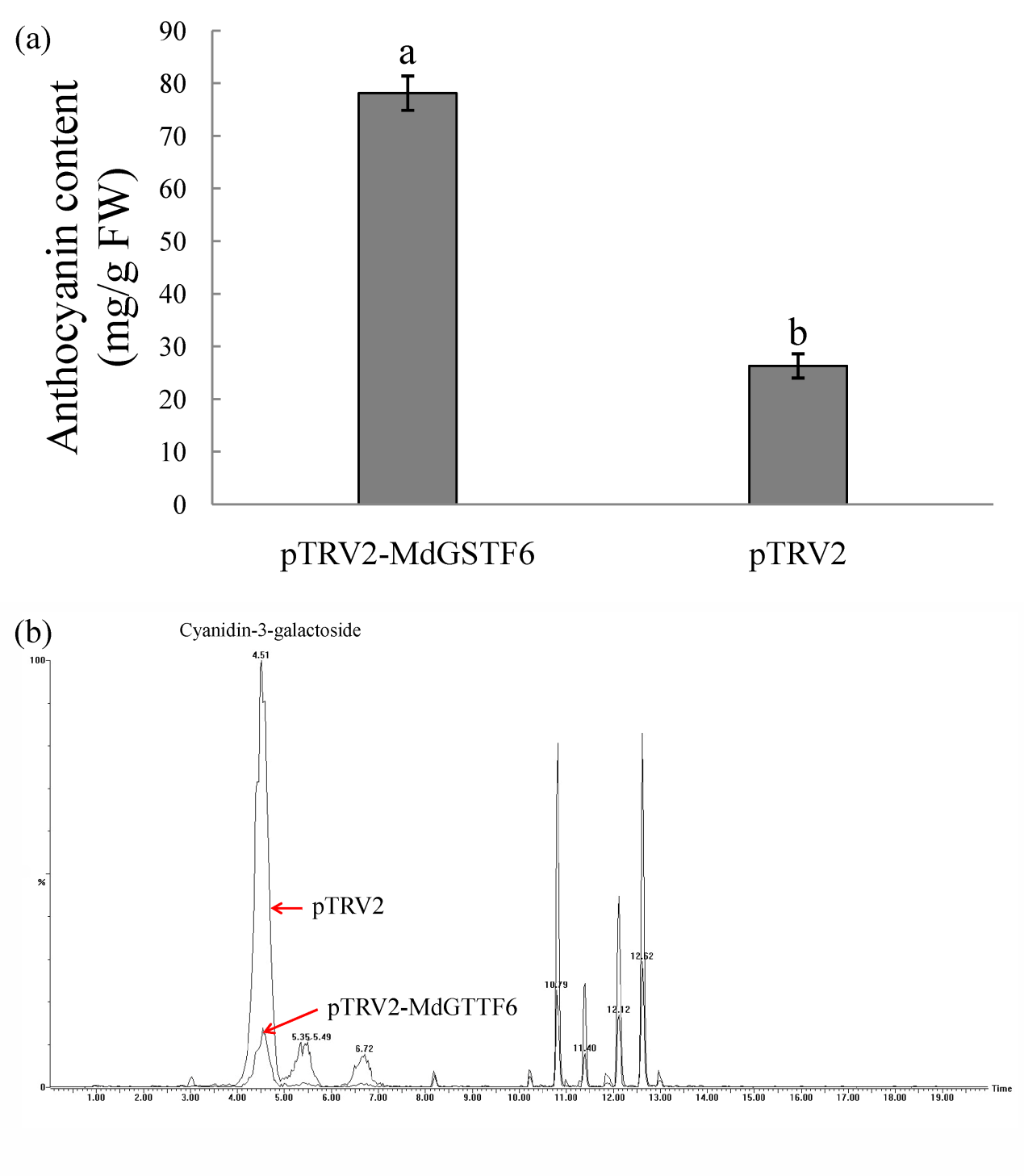


Fig. S6. MdGSTF6 silencing inhibits anthocyanin accumulation in apple skin. a The anthocyanin content of fruit skin treated by pTRV2-MdGSTF6 and pTRV2. b UPLC chromatograms of cyanidin-3-galactoside in fruit skins treated by pTRV2-MdGSTF6 and pTRV2. The y-axis showed the absorbance at 510 nm. Data are expressed as the means±SD, n = 3. The different letters denote significant differences according to one-way ANOVA test (P < 0.05).


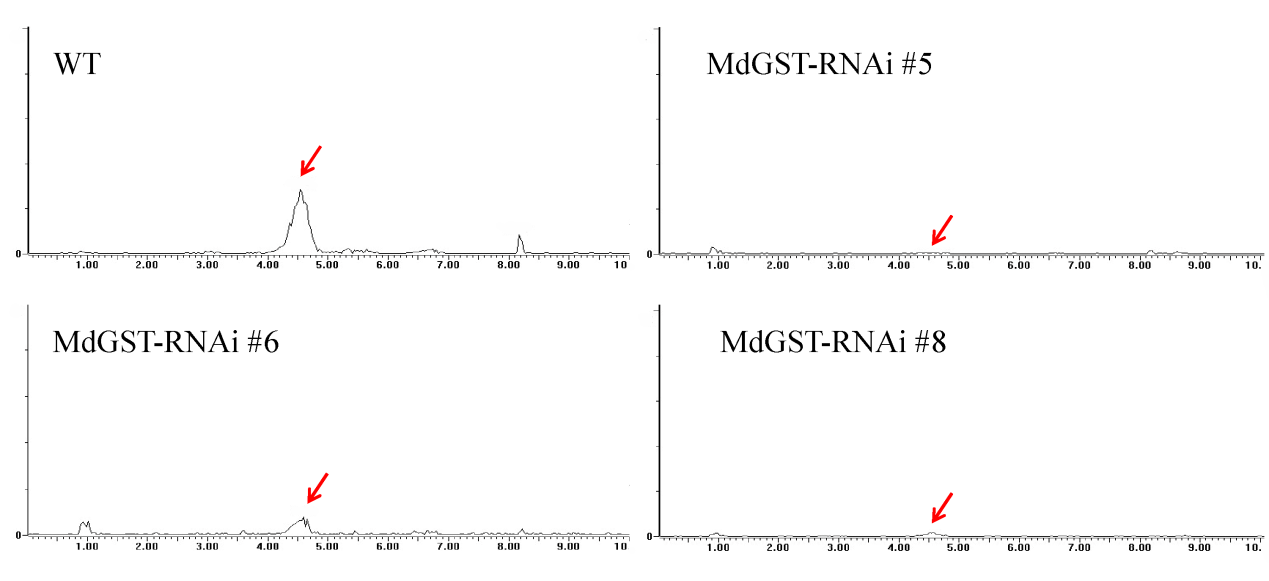


Fig. S7. UPLC chromatograms of cyanidin-3-galactoside in WT and MdGSTF6-RNAi ‘Gala’s. The red arrows indicated cyanidin-3-galactoside peak.


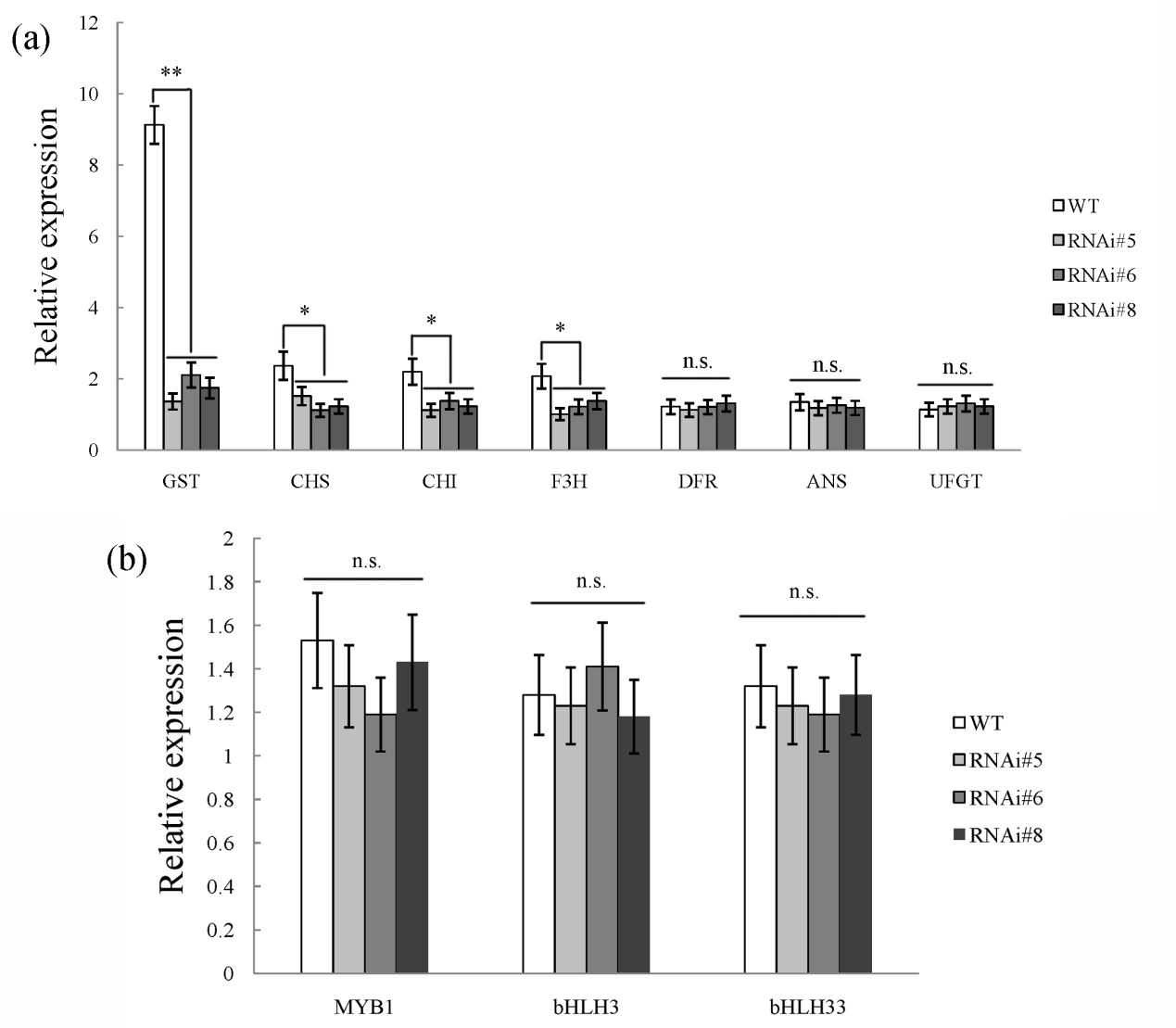


Fig. S8. MdGSTF6 silencing inhibits expression of genes involved in anthocyanin pathway in cultured ‘Gala’. a The expression of structural genes of anthocyanin biosynthesis in WT and MdGSTF6-RNAi ‘Gala’s. *CHS*, chalcone synthase; *CHI*, chalcone isomerase; *F3H*, flavanone 3-hydroxylase; *DFR*, dihydroflavonol 4-reductase; *ANS*, anthocyanidin synthase; *UFGT*, flavonoid-3-O-glucosyltransferase; *GST*, glutathione S-transferase MdGSTF6. b The expression of regulatory genes of anthocyanin biosynthesis in WT and MdGSTF6-RNAi ‘Gala’s. Error bars show standard derivation of three replicates, ‘*’ and ‘**’ indicate significance difference at P<0.05 and P<0.01, respectively.

Table S1. Primers used in this study.

| **Primers for quantative RT-PCR in apple** | | | | | | | | |
| --- | --- | --- | --- | --- | --- | --- | --- | --- |
| Gene name | | | | Forward | Reverse | | | Accession |
| MdGSTF1 | | | | GATGGCAATGATAGCAGTAG | GCTTCATATTCCTCCACAAC | | |  |
| MdGSTF2 | | | | GCCCTTCATATCCCTCAA | TCCCTTGTCAGCATACTC | | |  |
| MdGSTF3 | | | | AGTGCTTGAAGATGGTGAT | GGAAGACAATGGGAGAGAT | | |  |
| MdGSTF4 | | | | TCTCGTGTCTTAACCATTCT | TCAAGCACTGGAATCTGT | | |  |
| MdGSTF5 | | | | CAGTGGCTAGAGGTTGAA | TCGGGTCTGAAGGAAATC | | |  |
| MdGSTF6 | | | | AGTTGTAGAAGATGGTGACT | CAGGTCGTTGAAGTTGTG | | |  |
| MdGSTU1 | | | | GCTTTGCTTCTGAAATCCA | TTCCAGGTCTCATCAATGTA | | |  |
| MdGSTU2 | | | | TCAAGTCGGTGGAGTATG | CAGAAGGGAGGATAGATGG | | |  |
| MdGSTU3 | | | | TAGGAATCCGTTGCTGTT | TGACTCGTCTGCTTGATAA | | |  |
| MdGSTU4 | | | | GAGAAGGCTGTGGAAGAA | CCAGAAGGCTATGAAGTTG | | |  |
| MdGSTU5 | | | | GATGAGGTGGTGTTGTTG | ATCGGTTTCCCATTGTGA | | |  |
| MdGSTU6 | | | | GATGAGGACTTGAGGAACA | AGTGGAGCCTTGTCATTC | | |  |
| MdGSTU7 | | | | CTTGCCCTCTGACTGTTA | CGTCCTGTTCTTCTCCTT | | |  |
| MdGSTU8 | | | | GTTGTTCTTCTGGACTTCTG | GGCTTACCGTTGTGAATG | | |  |
| MdGSTT1 | | | | GTTCCTGCTATTGCTGATG | CGGCGTAAGTTAGAGTGA | | |  |
| MdGSTZ1 | | | | TGAAGAACTGCTGAACAAC | TGGAACCTAATGGCTGTAT | | |  |
| MdGSTZ2 | | | | TCCATTGTTACCACCAGAT | TGTAGAGGTTGTATGCTTGA | | |  |
| MdGSTL1 | | | | TGGTGGACATAGCCTATATC | ACCTTGTATGCCTCAATCT | | |  |
| MdGSTL2 | | | | CAACAAGATTGAGGCATACA | AAGAACGACTGAGCACAA | | |  |
| MdDHAR1 | | | | CATCTCCCTTATGACCTGAA | TCTTCCAGTGCTTGTGTAA | | |  |
| MdDHAR2 | | | | CGAAGTGGTGGAGGTTAA | CCTGCCTGTGAGAAGATTA | | |  |
| MdDHAR3 | | | | CAAGGATGGAACAGAAGAAG | AAATCAGCAGCAGAAACC | | |  |
| MdTCHQD1 | | | | TCCTCCTTCTGGTTCCTA | TCTCACTCTTTGGCTATCC | | |  |
| CHS | | | | GGAGACAACTGGAGAAGGACTGGAA | CGACATTGATACTGGTGTCTTCA | | | CN944824 |
| CHI | | | | GGGATAACCTCGCGGCCAAA | GCATCCATGCCGGAAGCTACAA | | | CN946541 |
| F3H | | | | TGGAAGCTTGTGAGGACTGGGGT | CTCCTCCGATGGCAAATCAAAGA | | | G104117 |
| DFR | | | | GATAGGGTTTGAGTTCAAGTA | TCTCCTCAGCAGCCTCAGTTTTCT | | | AF117268 |
| ANS | | | | CCAAGTGAAGCGGGTTGTGCT | CAAAGCAGGCGGACAGGAGTAGC | | | AF117269 |
| UFGT | | | | CCACCGCCCTTCCAAACACTCT | CACCCTTATGTTACGCGGCATGT | | | AF117267 |
| MYB1 | | | | TGCCTGGACTCGAGAGGAAGACA | CCTGTTTCCCAAAAGCCTGTGAA | | | AB744000 |
| bHLH3 | | | | ACCACCTCAGCCAGAACCT | CCTTCACCTTGGCTCTTAGTT | | | KX822759 |
| bHLH33 | | | | TCCGACAGAAGACTCCATGATG | CGTGTTTAGCAAAAGAGTGAGCC | | | DQ266451 |
| Actin | | | | TGACCGAATGAGCAAGGAAATTACT | TACTCAGCTTTGGCAATCCACATC | | | CN938023 |
| Atactin2 | | | | TCCCTCAGCACATTCCAGCA | GATCCCATTCATAAAACCCCAGC | | | NW_005361238.1 |
| **Primers for sequence analysis of MdGSTF6 promter** | | | | | | | | |
| Gene name | Forward | | | | | Reverse | | |
| MdGSTF6 promoter | GAAACTGTCGTTCGTTGAAATT | | | | | CTTCTTGTACAACAAAGTGCTAGC | | |
| **Primers for ChIP PCR** | | | | | | | | |
| Fragment | | Forward | | | | | Reverse | |
| G1 | | ACCCTCTCTCCCCATAACACA | | | | | ACTTGTTCCAATTATGACGTTTATAGC | |
| **Primers for subcellular localization** | | | | | | | | |
| Gene name | | Forward | | | | | Reverse | |
| MdGSTF6-pRI101 | | GTCGACATGGTTGTCAAAGTATATGGTCC | | | | | GGATCCGTAGTCACTAGCAAGCTGCATTAG | |
| **Primers for transgene** | | | | | | | | |
|  | | Forward | | | | | Reverse | |
| MdMYB1-HA | | GGATCCATGGAGGGATATAACGAAAACC | | | | | AGGCCTGGTTTTCGTTATATCCCTCCAT | |
| pMdGSTF6-GUS | | AAGCTTGAAACTGTCGTTCGTTGAAATT | | | | | TCTAGACTTCTTGTACAACAAAGTGCTAGC | |
| MdGSTF6-RNAi1 | | GGCGCGCCGGTCCCAACCTATTGGGA | | | | | ATTTAAATGTCACTAGCAAGCTGCATTAGTT | |
| MdGSTF6-RNAi2 | | ACTAGTGGTCCCAACCTATTGGGA | | | | | GGATCCGTCACTAGCAAGCTGCATTAGTT | |
| **Primers for Y1H** | | | | | | | | |
|  | | | Forward | | | | Reverse | |
| MdMYB1-pGADT7 | | | CATATGATGGCAAGGAGTGGGAATG | | | | GGTACCTTAACAGAAGAACATGGAGTTGATC | |
| MdGSTF6-pHIS2 | | | GAGCTCGAAACTGTCGTTCGTTGAAATT | | | | GAATTCCTTCTTGTACAACAAAGTGCTAGC | |
| **Primers for LUC assay** | | | | | | | | |
| Name | | Forward | | | | | Reverse | |
| 35S:MdMYB1 | | GGATCCATGGAGGGATATAACGAAAACC | | | | | AGGCCTGGTTTTCGTTATATCCCTCCAT | |
| 35S:MdbHLH3 | | GGATCCATGGCTGCACCGCC | | | | | AGGCCTAGAGTCAGATTGGGGTATAATTTGA | |
| 35S:MdbHLH33 | | GGATCCATGGCTCAGAATCATGAGAGG | | | | | AGGCCTGCACTTACCAGCAATTTTCCA | |
| pMdGSTF6:LUC | | GGATCCGAAACTGTCGTTCGTTGAAATT | | | | | CCATGGCTTCTTGTACAACAAAGTGCTAGC | |
| **Primers for VIGS** | | | | | | | | |
| Name | | Forward | | | | | Reverse | |
| MdGSTF6-TRV2 | | GGATCCGTCACTAGCAAGCTGCATTAGTT | | | | | CTCGAGTTGGGCAAGTTCCAGTTG | |
| **Probes for EMSA assay** | | | | | | | | |
| Name | | Forward | | | | | Reverse | |
| MdGSTF6-probe | | ATATCTAGTTGTAATCAACTGTTATGGCATAAATTG | | | | | CAATTTATGCCATAACAGTTGATTACAACTAGATAT | |
| MdGSTF6m-probe | | ATATCTAGTTGTAATCAGGTGTTATGGCATAAATTG | | | | | CAATTTATGCCATAACACCTGATTACAACTAGATAT | |

Table S2. Sequences used for phylogenetic trees and sequence alignment.

> MD17G1133600 MdGSTF5

MVVKVYGPFYAAPKRVLVCLVEKEIEFETSPIDLFKGEHKSPEFLKLQPFGQIPVIQDGDYTLYESRAIIRYYAEKYKSQGTDLLGKTIEERGLVEQWLEVEASNYHPPLDNLVMHILFASALGFPSDPKIIQESEEKLGKVLDIYEERLSKSKYLAGDFFSLADLSHLPFTHYLANSMGKEYMIRDRKHVSAWWDDISSRPSWKKVLQFGAPF

> MD03G1282700 MdGSTF2

MAAIKVHGNVISTAAMRVFATLYEKDIEFELVPIDMRAGEHKKEPFISLNPFGQVPAFEDGDLKLFGKFPFGQVPAFEDGDLKLFESRAITQYIAHEYADKGTPLVIRDSKKMAIISLWSEVEAQKFDPAATKLTYELAIKPMFKMTTDAAVVEENEAKLAVVLDVYETRLAQSKYLAGESFTLADLHHLPTIHYLMGTQSKKLFESRPHVLAWVVDITARPAWNKVVAQQK

> MD10G1196300 MdGSTU7

MADEVVLLDFWPSPFGMRLRIALAEKGVEYEYKDEDVRNKSPLLLQMNPVHKKIPVLIHNGKPVCESLIALQYIDEVWNDKAPLLPSDCYLRAQARFWADYVDKKIYEFGRKLWTTKGEEQDAAKKEFLDCMGVLEGELGDKPFFGGETIGFMDVALIPFYSWFLLYEKFGNFSVEALHPKFIAWVKRCMEKESVSKSLPEQEKVYDFVMLMRKKLGME

> MD10G1197100 MdGSTU8

MADEVVLLDFWASMFGMRARVALAEKGIEYEYREEDLRNKSQLLLQMNPVHKKIPVLIHNGKPVCESLIIVQYIDEVWSDKAPLLPSEPYQRACSRFWADFIDKKLYDAGRKIWSTKGEEQEAAKKDFIEILKQLEGQLGGKPYFEGEKFGFLDIALITFYSWFHAYETCGNFSIEAECPKLIAWAKRCKQRESVSKSLADEKKVYEFVLSIKKMLGVE

> MD15G1133600 MdTCHQD1

MQLYHHPYSLDSQRVRLALEEKGIDYTSFHVNPVTVKNMDASFFRRNPSAKLPVFQNGHHIIYNTIEIIQYVERIALVSSGEENIPSSGGEVTAWMHRIKQWNPKFFTLSHIPKKHRDYVYKFIRQVIIARMAEATDLAAAYHAKLVQVYETEDKLKDPAVLNQSKEQLIRLLDQVEKKLNESAYLAGEEFSMADVMLVPVLARLVLLNLKEEYIDGRPNTAKYWIMVQQRPSYKKVIGKHFSGWRKHKTFIRTWCFVHIRTILRRF

> MD17G1260600 MdDHAR1

MLTTAKIHPAASAVLSSSIKHHLRPPPNAVVFRTNPNSLRRRGTTRTLTVAMVAPLDVCAKASTTVPNKLGDCPFCQRVLLTLEEKHLPYDLKLVDLGNKPEWFLKINSEGKVPVVKLDEKWVADSDIITQALEEKYPEPPLATPPEKASVGSKIFSTFIGFLKSKDPKDGTEQALLNELSSFNDYLKENGPFINGKEVSAADLSLGPKLYHLEIALGHFKDWSIPDSLPYVKSYMKSIFSLDSFVKTSALKEDVIAGWRPKVLG

> MD06G1012200 MdGSTF4

MGVKVHGVAVSTCTSRVLTILHEKGLDFELVPVNLLAGEHKQPPFLAKNPFGQIPVLEDGDLTLFESRAITAYVAEKFKENGVDLIRHNNLNEAALVKVWTEVESQSFHPAISPIVFQHIIVPMRGQTPDQAVIDANLEKLAKVLDVYEDRLSSTKYLAGDFYSLADLHHLSYTYYFMKTPGASLVNERPHVKAWWEDISSRPAFKKVAEAMTLGQK

> MD12G1129400 MdGSTL1

MAYRHEDLPPPLDSTSSPPPLFDGTTRLYVNYSCPFAQRVWITRNYKGLQDKIKLVPINLQNRPDWYKEKVYPGNKVPALEHNGKVIGESLDLIKYIDSNFEGPSLSPKDDPERKKFGEELLTYVDTFTGSLYRSFKGDAVKAADEQFDYLENALKKFDDGPYFLGQFSLVDIAYIPFIERFQVFLSDVFKYDITAGRPKLAAWFEEINKIEAYKVTKTDPKELVAFYKKRFLEQQ

> MD15G1445700 MdGSTT1

MMEAQEVEVEVEAQAATESGDQQIKLYSYWRSSCSYRVRIALNLKGLKYEYKAVNLLKGEQFSPEFRKLNPVGYVPVLVDGDTLVADSFAILMHLEEKYPQHPLLPPDLQKRAINYQAANIVSSSIQPLQNLAVLKYLEEKVSPDEKIEWVKFHIGKGFSALEELLNNHAGKYATGDEVYMADLFLAPQVYTAIRFQLDMTQFPLLARLHEAYKKIPAFLDAIPEKQPDAPSP

> MD09G1264700 MdDHAR3

MSTTARIHPTASAVLSTTIKHHLRPPPNAVVFRTNPNSLRRRGTTRTLTVAMAAPLDVCAKASVTVPNKLGDCPFCQRVLLTLEEKHLPYDLKLVDLGNKPEWFLKVYPEGKVPVVKLDEKWVADSDIITQALEEKYPDPPLATPPEKASVGSKIFSTFIGFLKSKDAKDGTEEALLNELKSFDDYLKENGPFINGKVVSAADFSLGPKLYHLEIALGHFKDWSIPDSLPYVKSYMKSIFSLDSFVKTSALKEDVIAGWRPKVLG

> MD17G1272100 MdGSTF6

MVVKVYGPVMAACPQRVMVCLLEKGVNFEIVDVDLEAGEHKRPEFLTRQPFGQVPVVEDGDFRLFESRAIIRYYAAKYAGRGPNLLGTTLEEKAVVDQWLEVEGHNFNDLVYTLVLQLLVLPRMGQRGDAALINACEEKLEKVFDVYEERLSKSKYLAGETFTLADLSHLPGIRYLIDEAKLGHLVTGRKKVNAWWEDISNRPSWKKLMQLASDY

> MD06G1012200 MdGSTF3

MGVKVHGVAVSTCTSRVLTILHEKGLDFELVPVNLLAGEHKQPPFLAKNPFGQIPVLEDGDLTLFESRAITAYVAEKFKENGVDLIRHNNLNEAALVKVWTEVESQSFHPAISPIVFQHIIVPMRGQTPDQAVIDANLEKLAKVLDVYEDRLSSTKYLAGDFYSLADLHHLSYTYYFMKTPGASLVNERPHVKAWWEDISSRPAFKKVAEAMTLGQK

> MD04G1112000 MdGSTL2

MAYKHEELPPPLDSTSSPPPLFDGTTRLYVNYTSPFAQRVWITRNYKGLQGKIKLVGIDLENRPAWYKEKVYPGNKVPSLEHNGKVIGESLDLIKYLESNFEGPSLVPNDDPGRKKFGEELLTYVVTFVGSLYRSLKGDTVKAADEQFDYLENALKKFDDGPYLLGQFSLVDIAYIPFVEQFQVFLSDVFKYDITAGRPKLAAFLEVEFEINKIVAYKVTKADPKEIAAAYKKKFLEQQ

> MD06G1081000 MdGSTZ1

MTKNEEKIHQKEEMKLKVYVDRLSQPSRAILIFLKVNGIEFEEIKMDISKRQHLSPEFKKINPMGQVPAIADGRFNLFESHAILVYLACAFPGVADHWYPADLFRRAKINSVLDWHHSNLRRGAAPYVLNTVLAPVLGLPLNPQAAAEAEKLLSSSLSKIESIWLQGNGKFLVGGLKPSIADLSMVCEIMQLELVDEKDRNRILGPHKRVLQWIEDTKNATRPHFDEVHEVLFRAKTRFQERRLMGASNKTDSSDRAAVRSKM

> MD01G1120300 MdDHAR2

MALEVAAKAAAGAPDLLGDCPFCQRVTLTLEEKKVPYKLHLINLSDKPKWFTEVNPEGKVPVVKFDDKWVPDSDVIVGIIEEKYPEPSLKTPPEFASVGSKILGSFVTFLKSKDPGDGSEQALLTELKALDEHLKAHGPYIAGEKVTAADLSLAPKLYHLKVALGHFKKWTVPADLAHYHKYTELLFSRESFVKTAPADEKYVIAGWEPKVNP

> MD04G1139500 MdGSTU1

MAEEVKLLGVWGSPYSRRVEIALKLKNVEYEFVEEDLQSKSALLLKSNPVHQKIPVLLHNGKPLAESQVILEYIDETWKEGFPILPKDPYERAQARFWARFIDEKCLPATYKVLWGCEEHEKAVEEACELLKILENELKDKKFFAGETVGLVDIVANFIGYWLRAIQEVVGVELLTKEKLPKLYNWSDEFCSVFQESLPPKDKLVAHIRGRLQSTNTTTTSS

> MD08G1250500 MdGSTZ2

MEAQEVEAQAATGSGQQQLKLYSYWMSSCSFRVRIALNLKGLKYEYKALAKGEQFSPEFRKLNPMGYVPVLVDGDTVVADSFAIILYLEEKYPQHPLLPPDLQKKAINYQAANIVSSSIQPLQNMTVLKYIEEKVRPVEKLEWVQFHIGKGFLALEELLNNHAGKYATGDEVYMADLFLAPQLYAAITRFQLDMTQFPLLARLHEAYNKIPAFLDALPEKQPDAPS

>MDP0000487037 MdGSTU3

MTTASWRKERIALSPLKELIAXAIACSGRDGXRKRSXTARDVGYTYSKRVELALRLKGVTYEYIEEDLSNKSQQLLEYNPVHKKVPVLVHNGKPIVESYVILEYIDETWNNAPKILPEDPYERAKVRFWASYIQQQLFEGMSRVVTSQGEAQEKALEEVFASLGVFEEGMKEYLHGGDPFTNGENLGLLDILMVATFGPYKAHEQVLGFKMLDPDRNPLLFSWVAATNEHPLVKELDPPHDKLVQLLQFIKQTSHARSH

> MD03G1282900 MdGSTF1

MAPVKVHGNVLSVCTRRVIAALYEKDIKFELVPIDLGTGEHKKEPFISLNPFGEVPAFEDGDLKLFESRAITQYIVHEYADKGTPLVFQDSKKMAMIAVGCEVEGQKFDPAASKLTFEQVIKPMLKMPTDAAVVEEYEAKLAVVLDVYEIRLAQSKYLAGERFTLADLHHIPSIHYLMGTQSKKLFVSRPHVSAWVADITARPAWKKVIALQK

> MD05G1210600 MdGSTU5

MADEVVLLDFWPSPFGMRLRIALAEKGIVYEYKDEDIWNKSPLLLQMNPVHKKIPVLIHNGKPVCESLIALQYIDEVWNDKAPLLPSDSYLRAQARFWADFVDKKIYEIGRKLWTTKGEEQDAVKKEFLDCIGVLERELGDKPFFGGNTLGFVDVALIPYYSWFLVYEKFGNFSVEAEHPEFIAWVKRCMEKKSVSKSLPEQEKVYDFVMLVRKKHGI

> MD04G1139400 MdGSTU2

MAEVKLLGSWGSPFSRRVEAALKLKNVEYEFVDEDLQSKSALLLKSNPVHQKIPVLLHNDRPIAESQVILEYIDETWKEGFPILPKDPYERAQARFWARFIDEKCLPATWKALWGSEEPEKAVEEACELLKILENELKDKKFFAGETVGLVDIVANFIAFWLRAIQELVGVELLSKEKLPKLYNWSDEFCSVFQENLPPKDRLVAHFRGRLQSTTTTTTSS

> MD16G1081100 MdGSTU4

MAKSNVKVLGAWPSPYVMRARVALNIKSVEYEFLEETFGSKSRLLLQSNPVHKKIPVLIHGDKPVCESLIIVEYIDEVWSSGPSILPSDPFDRATARFWAAYIDEKWFPAMKGIGAAQGDDARKAAVEQVAEGLAQLEEAFQKTSKGKDFFSGDKIGYLDIAFGCFLGWLRVTEKMNGVKLLDGAKIPGLVTWAEKFSADPAVKDVMPETDKLAEFAKILAAKMRGAGAPK

> MD05G1210000 MdGSTU6

MADEVVLLDFWPSPFGMRLRIALAEKGIEYEYKDEDLRNKSPLLLQMNPVHKKIPVLIHNGKPVCESLIALQYIDEVWNDKAPLLPSDSYLRAQARFWADFVDKKIYDIGRKLWTTKGEEYDAAKKDFLDCIGVLEGELGDKPFFGGDTLGFVDVALIPFYSWFLVYEKFGNFSVEAVHPKFIVWVKRCMEKESVSKSLPDQEKVYDFVALMRKKFGIE

> MD05G1184400 MdGSTU35

MAGVKLLGAWSSPYVYRVIWALKLKGVEYEYVEEYVLFNKSDELLKYNPVHKKVPVLVHDGKPTAESIIILEYIEEAWPHNPLLPKDPHQRAEARFWAKFGEDKNRAFIGLFFATGEEQVKAIKEAQENLKILEEHGLGNKKFFGGNDIGLADLAFGWMALWLEVLEEAASVKVFEGDSFPRLHAWIQSFKESPTIKENLPERSALLNYFKGGRATVVASAQS

> MD17G1134300 MdGSTF11

MLLRHKKTTNGWMYTCEQPHPPSTPILNSSIQLLECYIIRSKEPEAGVCRFDLNPSVLTLRSHNMVVKVYGPLYAAPKRVLVCLVEKEVHFETVPIDLLKGENKHPDFLKLQPFGTVPLIQDGDYTLYESRAIMRYYAEKYKSQGTDLLGKTIEERGLVEQWLEVEAHNFQPPIYNLVVHILFAPVLGFPSDPKILQESEEKLGKVLDIYEEQLSKSKYLAGDFFSLADISHLPFTHFLVANMGKEYMIKDRKHVSAWWDDISNRPSWKRVLQFGDPF

> MD02G1236000 MdGSTU27

MAEVKLLRSWSSPFGLRIVWALQLKGIPYEAIYEDLSNKSPLLLQSNPIHKKIPVLLHNGNAVVESLIILEYVDETWKENPLLPEDPLERATARFWARYCDETVSPSIWESFTKEGKEQEEGIVKAKENLKYLEEELKGKKFFGGEKIGIVDIALGWLAYYESLFEDIVGMKVVTKEEFPLLSAWAATFADVPIIKDNWPSKDKLIPKFQAIRDSSLKK

> MD02G1236100 MdGSTU26

MAEVKLLRTWSSPFGLRIVWALQLKGIPYETIYEDLSNKSPLLLQSNPIHKKIPVLLHNGNAVVESLIILEYVDETWKENPLLPEDPLERATARFWARYGDEKVLPSIWESFTKEGKEQEEGIVKAKENLKYLEEELKGKKFFGGEKIGIVDIALGWLAYYESLFEDIAGMKVVTKEEFPLLSAWAATFADVPIIKDNWPSKDKLIAKFQAIRDSIVKE

> MD10G1172300 MdGSTU34

MAEVKLLGAWPSPFVQRVIWALKLKGVEFENVEEDVLFNKSDELLKYNPVHKKIPVFVHNGKPVAESIVILEYIDEAWPHNPLLPKDPHLRAEARFWAKFGEDKDRAFRGFFFVTGEEQVKAVKESQENLKILEEHGLGNKKFFGGEEIGLVDLAFGWIACWLEVWEEVAGVKVFEADRFPRLQAWIQRFREVPTIKENLPDRTALLTFFKSRRAMIIALAKP

> MD10G1196900 MdGSTU16

MADEVVLLDYWASKFGMRARVALAEKGVKYEYREEDLRNNIKSQMLLQMNPVHKKTPVLIHNGKPVCESLIIVQYIDEVWRDKAPLLPSEPYQKAHSRFWADFIDKKLYDACTKIWMTKGEEQEAAKKDFIEILKQLEGQLGDKPYFEGEKFGFLDIALITFYCWFHAYEICGNFSIEAECPKLIAWAKRCMQRESVSKSLADEKKVYELVLGLQKMFGWE

> MD13G1227600 MdGSTU30

MSEVKLHGAWPSPFSCRVIWALKLKGIPYDNIEEDLPNNKSPQLLKYNPVHKKIPVLVHGEKPICESMVIVEYIEETWPQKPLLPTDPYERATARFWVKFAEDKGPAIWMVFRTTGEVQEKAKKDSLEMLRTIEDHAAGTLGKKKFFGGDNIGIVDIAFGGIAHWFGVIEDVVEVRLFEAKEFPRLYAWTNDFKQVPAIKENLPDRHKLLLLFKQIRENLLASIM

> MD05G1210400 MdGSTU11

MADEVVLLDSWQSPFGMRLRIALAEKGIEYEYKDEDLWNKSPLLLQMNPVHKKIPVLIHNEKPICESLFALQYIDEVWNDRAPLLPSDSYLRAQARFWADFVDKKKIFQIGKKVWTTKGEKQDAAKKKFLDCIGVLEGELGDKPFFGGDTLGFVDVTLIPIYSWFLVCEKFGDFSVEAEHPKFIAWVKRCMEKESVSKSLPEQEKVYDYAMLIRKKLRIE

> MD05G1210700 MdGSTU13

MADEVVLLDFWPSPFGIRLRIALAEKGIVYEYKDEDLWNKSPLLLQMNPVHKKIRVLIHNGKPVSESLIALQYIDEVWNHKAPLLPSDSYLRAQARFWADFVDKKMYEIGRKLWATKGEEQDAAKKEFLDCIGVLERELGDKPFFGGNTLGFVDVTLIPYYSWFLVYEKFGNFSVEAEHPEFIAWVKRCMEKESVSKSLPEQEKVYDYAMLIRKKLGIA

> MD09G1147100 MdGSTF9

MVVKVYGPLYASPKRVLVCLVEKEVDFETVPIDLLKGEHKHPDFLKLQPFGSVPLIQDGDYTLYEKYKSQGTNLLGNTIEERGLVEQWLEVEAQNFHPPINNLVLHILFASVLGFPSDRKIIQESEEKLGKVLDIYEERLSKSKYLAGDFFSLADLSHLPFTHYLVANMGKEYMIRDRKHVSAWWDDISNRPSWKRVLQFGAPF

> MD04G1139600 MdGSTU23

MAEEVKLLGVWGSPFSRRVEIALKLKGVKYEYFEEDLQSKSALLLKSNPVHQKIPVLLHNDRPIAESQVILEYIDQTWKEGFPILPKDPYERAQARFWARFIDEKCLPAAWKALWGSEEPEKAVEEACELLKILENELKDKKFFGGETVGLVDIVANFIAYWLGAIQEVVGVELLTKEKLPDLYDWSDEFCSAFHESLPPRDKLVTFFRRRFQSTTTATSN

> MD17G1133800 MdGSTF10

MVVKVYGPLYAAPKRVLVCLVEKEVDFETVPIDLLKGENKHPDFLKLQPFGTVPLIQDGDYTLYESRAIMRYYAEKYKSQGTDLLGKTIEERGLVEQWLEVEAHNFQPPIYNLVVHILFSPVLGFPSDPKILQESEEKLGKVLDIYEEQLSKCKYLAGDFFSLADLSHLPFTHFLVANMGKEYMIRDRKHVSAWWDDISNRPSWKRVLQFGDPF

> MD05G1210200 MdGSTU10

MADKVVLLDFWPSLFGMRLRIALAEKGIEYEYKEEDLSNKSPLLLQMNPVHKKIPVLIHNGKPVSESLIALQYIDEVWNDKAPLLPSDSYLRAQARFWADFVDKKIFENRKKLRTTKGEEYDAAKEDFLDCIGVLEGELGDKPFFGGETLGFVDVALVPLYSWFLVYEKAGNFSVEAEHPKFIAWAKRCMEKESVSKSLPEQEKVYDFFMLTRKKRGIE

> MD05G1210100 MdGSTU9

MADEVVLLDFWPSPFGMRLRIALAEKGIEYEYKEDDLWNKSPLLLQMNPVHKKIPVLIHNGKPVCESLIALQYIDEVWNDKAPLLPSDSYLRAQARFWADFVDKKIHENGRKLWTTKGEEYDAAKKDFLDCIGVLEGELGNKPFFGGETLGFVDVALVPFYSWFLVYEKFGNFSVEAEHPKFIAWVKRCMEKESVSKSLPEQEKVYDFFMLIRKKRGIE

> MD09G1279700 MdGSTU17

MSTEDVVLLDCWVSPFCMRVKIVLEEKGIQYEERAEDLFGGKSELLLTSNPIYTKVPVLLHKGKPVCESSIIVGYVDETWASPPLLPSCPYARAQAKFQADYIDKKLYDAGSSIWRTKGEAQEVAVKDFIEVLKQLEKFLGDKDFFGGDSFGFVDIITIGITSWFSAYEKFGNFKVEDHTPKLSAWINRCMQRKTVAKVIPDPEKVYEFVINLKKMMGFE

> MD09G1147300 MdGSTF12

MVVKVYGPVYPSPKRVLVCLVEKEIEFETVPIDIFKGEQRNPEFLKLQPFGQVPVIQDGDYTLFESRAIIRYYAEKYKSQGTDLLGKTMEERGLVEQWLEVEASNYHQPLDNIVTHILFASAMGISSDPKIIQESEEKVGKVLDIYEERLSKSKYLAGDFFSLADLSHLPFTHYLANSMGKEYMIRDRKHVSVWWDDISSRPSWKKVLQFGECNECICLPRNIAT

> MD05G1211000 MdGSTU12

MADEVVLLGFWSSPFGIRLRIALAEKGIVYEYKEEDIWNKSPLLLQMNPVHKKIPVLIHNGKPVSESLIALQYIDEVWNDKAPLLPSDSYLRAQARFWADFVDKKIFEFGRKLWATKGEEQNAAIKEFLDFIGVLERELGDKPFFGGDTLGFVDVTLISYYSWFLEYEKFGNFSVEAEHPEFIAWVKRCMEKESVSKSLPEQEKVYDYAMFLRKKFGIE

> MD17G1134200 MdGSTF8

MGLCTAPQSVCWCALLRKKEVDFETVSIDVLKGEHKHPDFLKLQPFGSVPLIQVGDYIYSIWYYAEKYKSQGTNLLGKTIEERGLVEQWLEVEAHNFHPPSYNMVLHILFASVSGFPSDPKIIQESEEKLGKVLDIYEERLSKSKYLAGDFFSLVDLSHLPLTQYLVANMGKEYMIWDRKHVSAWWDDISNRPSWKRVLQFGAPF

> MD08G1006600 MdGSTF13

MATPVKVYGPPLSTAVSRVLACLHEKGVDYQLISVNMAKGEHKKPDYLKIQPFGQVPAFEDEDISLFESRAICRYICNKYADKGNKGLYGMNPLAKASIDQWLEAEGQSFSPPSSTLVFQLAFAPRMKLKQDEGVIRQNEEKLAKVLDVYEKRLGGSRFLAGDEFSLADLSHLPNTQYLVNATDRGELITSRENVGRWWSEISSRESWQKVVEMQKPT

> MD04G1033800 MdGSTU15

MGDGDEVVLVGFWTSPYVMRVKIALAEKGVHYLYLEEEHLLQNNKSPLLLEVNPVHQKVPVLIHNNKPVCESLIILQYIADVWSDHKPPLLSEDPYRRSKERFWVDFFNKEIADCGRRMWASKGADQEAAKNEFVEVLKLLEGELGEKPYFEGDRFGLLDVTLVPFACRFYTYEMFCNFSVEKECPKIMQWVKRCSLRESVSKTLPDKYKVYDFVLEVKKMLGIN

> MD14G1232200 MdGSTU25

MGDVKVIGASLSLFCCRIEWALKHKGIAYEYIEEDLRNKSHLLLRYNPVHKKVPVLVHGEKPVAESLVILEYIDETWRENPLLPEDPFEKAKARFWAKYVDEKCVISAWTASRTKGHEQEKSLEAARESLEVLNKLIEGKKFFGGEIIGFLDLVVGTLPNWLKFLEESEGIKLFDTKELPFLHEWAQRFTEIPIIKGSIPTAEDLINYNRDRQKAE

> MD17G1271700 MdGSTU19

MATEDVVLLDLWASPYCMRVKIALEEKGIKYEERAEDLFGGKSELLLTSNPVYAKVPVLLHKGKPLCESSIIVGYIDETWASPPLLPSCPYARAQAKFWVDYVDKKLFDAGGNIWRTKGEAQEVAVKDFIEILKQLEKVLGDKDFFGGDSFGFVDIITIAITSWFLAFEKFGNFKVEDHAPKLSAWIKRSMQRETVAKVIPDPEKIYEFVINLKKMMGME

> MD13G1081900 MdGSTU22

MGESDVKVLGMAPSPFVMRARIALNLKSVDYEFLQETFGSKSELLLQSNPVHKKVPVLIHGDKPVCESLIIVEYIDEVWASGPSILPSDPYDRATARFWAAYISEKWYPSMKGIGLAQGEEAKKAAIEQVTEGLALLEEAFEKSSKGNVFFGGDEIGYLDIAFGCFLGWLRVNEKLHGIKLLDQTKTPGLVKWADKFCAHAAVKDVMPETDKLVEIAKILAAKARAAAAPPPSN

> MD15G1438400 MdEF1B2

MALVLHAGKTNKNGYKALITAEYTGVKVELAPNFDMGVSNKTPEYLKLNPIGKVPLLVTPDGPIFESNAIARYVARLKADNPLYGCSLIDYAHIEQWIDFGSMEIDANISKWYYPRLGYGVYLPPAEEAAISALKRALGALNTHIASNTYLVGHSVTLADIVVVCNLYVGFANVMTKSFTSEFPHVERYFWTLVNQPNFKKVLGDVEQAVSVPPVASAKKPAQPAKGKIKEEPKKEAKKEPAKPKAEAAEEVEEAPKPKPKNPLDLLPPSKMVLDDWKRLYSNTKSNFREVAIKGFWDMYDPEGYSLWFCEYKYNDENTVSFVTLNKVGGFLQRMDLARKYAFGKMLVIGSEAPFKVKGLWLFRGQEIPKFVMYECYDMELYSWTKVDISDENQKERVNQMIEDQEPFEGEALLDAKCFK

> MD16G1232900 MdGSTU29

MCLYLYNIIFHIISNPAATRGQNFLVGSKAQAELLLQCNPVYKKVPVLFHRGKPISELIAILECIEETWPENHLLPEDAYERALARFWIQFHIPSFNAFFGLTAGEDRQKTIESVLETLKILEEQGLGDKKFFGGDSINLVDITHGWLALYFEAVEEMVGVKLLEPSTLPRLHAWVQNFKQVPVIRDNLPNYQILVAHMKRAREMLVPQI

> MD13G1082100 MdGSTU20

MSFVRFLCCEWLPSLRGVAAAEGDEAKKAAVEQVAEGLAQMEEAFQKTSKGKDFFSGDKIGYLDIAFGCFLGWLRVTEKMNGVKLLDEAKIPGLAKWAEKFSADPAVKDVMPETDKLAEVTKILKARLRAAAVLVESEGVKAEKMQKCLHFSSLGRVGGCKSRDDAKVLAFRKMSKAKGKADTALPWANRKYNRFKGQLKTQETE

> MD01G1115000 MdGSTL3

MAALKVSLRGTAAPTCSPSPSLKSWPHISDSLSKTSAIARFPNAAVLSPPKLHLQASPGKKTRASVSATMATGGQEVLPPALTSTSDPPPIFDGNTRLYISYQCPYAQRAWIARNCKGLEEKIQLVPIDLQDRPAWYKEKVYPANKVPSLEHNNEVKGESLDLIRYIDSHFEGPSLFPDDQAKKEFAEELLSYTDTFNKSVFSSIKEDEIDAAGAAFDYIETALSKFEDGPFFLGKFSLVDIAYAPFLERFQPLLLDVKKYDITAGRPKLAAWIEEMDKNVAYKQTRRDPKELVESYKRRFLAQK

> MD07G1107900 MdGSTF7

MLNSHGRLQSTTPRRIVLIDSHGQQQPKTRSTNHDRVHDSHLDSRASFAAERSPVVSENLTDSVVDMRLGELALRTHSSAAKSASSDEEYLQLSQAFSDFSACSSDISGELQRLASLPSPENGPTSESAEAAPEPEPCQGFLQRENFSTEIIESISPEDLQPTVKICVDGLQSPSLAVKQSAAAKLRLLAKNRADNRALIGESGAVPALIPLLRCSGRRSTPSQQTVIVGGERGRLLYRSENESRNVCGSAKYFTDAVALIFAKDKGKPHGIGDIQFQIALSNGVSTVGPRHKAVSVKVNECDRSTWLTALKRALSALNTHIASNTYLVGHSVTLADIVVVCNLYVGFANVMTKSFTSEFPHVEKYCWTLVNQPNFKKVLGDVEQAVSVPPGAFAKKPAQPAKGKTKEEPKKEAKKEPACHASSAHTPHVGIL

> MD17G1286500 MdGSTU18

MATEDVVLLDLWASPYCMRVKIALEEKGIKYEERAEDLFGGKSELLLTSNPVYAKVPVLLHKGKPLCESSIIVGYIDETWASPPLLPSCPYARAQAKFWVDYVDKKLFDAGGNIWRTKGEAQEVAVKDFIEILKQLEKVLGDKDFFGGDSFGFVDIITIAITSWFLAFEKFWKLQG

> MD13G1082000 MdGSTU21

MAKSDVKVLGAWPSPYVMRARIALNVKSVEYELLEETFAPKSQLLLQSNPVHKKIPVLIHGDKPVCESLIIVEYIDEVWASGPSILPSDPLDRATARFWAAYVDEKWFPSLRGIGAAEGDEARKAAVEQVAEGLAQMEEAFQKTSKGKDFFSGDKIGYLDIAFGCFLGWLRVTEKMNGVKLLDEAKIPGLAKWAEKFSADPAVKDVMPETDKLAEVAKIIMARLRAAGASK

> MD14G1232100 MdGSTU24

MGVVKLFATEGSVFCARIQWALKLKGVEYEFILEDLANKSPLLLKYNPIYKKVPVLVHGENVIVESLVILEYIEDTWKEHPLLPRDPYDRATARFWAKFNDEKLVLSVWTACTGEGEAQEKAKESALESLALVEKQIEGKKFFGGEQIGYLDLVLGWIPHWISAMEEAGGNKVPEAEMFPSLHQWGQNFIHTPLIEECIPAKEALVTYLRFMIGYMRSLSANKP

> MD05G1209700 MdGSTU14

MAEEVVLLDFWPSPFGMRVRIALAEKGVEYESREEDLSNKSPLLLKMNPVHKQIPVLIHKGRPICESLIIVQYIDEVWSDKAPLLPSDPYPRAHARFWADYVDKKIYSIGKLVWVTTGEVQEAAKKELIECFKLLENELGDKTYFGGESFGLVDVALIPFYSWFYALETCGNMCMVKECPRLVGWAKRCMQRESVSESLPDQYKIPRGSKEGTDRECFKLLENELGDKAYFRGESFVLVDVALIPFYSWFYALETCGNLCMVKECPRLVGWAKRCMQRESVSKSLPDQYKMYDFLLELRKKFDVHT

> MD05G1251900 MdGHR5

MAQTTEISQSGAFLRTASVFRNFISRDPNSQFLAEPGRYHLYISYGCPWASRCLAYLKIKGLEKAISFTSVRPTWGRTKETDEHMGWVFPASDTEVVGAEPDPLNGAKSIRELYELASSQYTGKYTVPVLWDKKLKTIVNNESAEIIRMFNTEFNDTAENASLDLYPSHLQSQIDQTNEWIYDKINNGVYKCGFARKQEAYDEAVKELFEALDKCEEILSKQRYLCGNTLSEADIRLFVTLIRFDEAYAVNFKCNKKLLREYPNLFNYTKDIFQVPGVSSSVNMDHIKRGYYTMAAINPSGIIPIGSNIDYSSPHDRDRFST

> MD05G1252400 MdGHR3

MEISQSGAFVRAPSVFRNFISRDPNSQFPAEPGRYHLYISYLCPWACRCLAYLKLKGLEKAISFTSVKPKWERTKESDEHMGWVFPASDTEVAGAEPDPLNGAKTIRELYDLASTHYTGKYTVPVLWDKKLKTIVNNESSEIIRMFNTEFNDIAENASLDLYPPHLQSQIDQTNEWIYNMINNGVYKCGFARKQEPYDEAVKELFEALDKCEEILSKQRYLCGNTMSEADIRLFVTLIRFDEAYAVNFKCNKKLLREYPNLFNYTKDIFQVPGVSSTVNIDHIKRGYYNIAAINPFGIIPVGPHIDYSSPHDRDRFST

> MD10G1233400 MdGHR2

MARSALDETSLSGAFVRTASVFRNFISRDPNSQFPAEPGRYHLYISYACPWASRCLAYLNIKGLDKAISFTSVKPIWERTKESDEHMGWVFPASETELAGAEPDPLNGAKSIRELYELASTQYTGKYTVPVLWDKKLKTIVSNESGEIIRMFNTEFNDIAENASLDLYPPHLQSQIDQTNEWIYDKINNGVYKCGFARKQEPYDEAVKQLYEALDKCEEILSKQRYLCGNTLSEADIRLFVTLIRFDEVYVVHFKCNKKVLREYPNLFNYTKDIFQVPGMSSTVNMDHIKRHYYGSHPSINPFGIIPSGPDIDYSSPHDRNRFST

> MD05G1252100 MdGHR4

MAQTTEISQSGAFLRTASVFQPGRYHLYISYGCPWASRCLAYLKIKGLEKAISFTSVKPMWGRTKESDEHMGLLFPASDAEVAGAEPDPLNGAKSIRELYELASTQYSGKYTVPVLWDQKLKTIVNNESAEIIRMFNTEFNDMAENASMDLYPPHLQSQIDQTNEWIYDMINNGVYKCGFARKQEPYDEAVKELFEALDKCEEILSKQRYLCGNTLSEADIRLFVTLIRFDEAYAVNFKCNKKLLREYPNLFNYTKDIFQVPGVSSSVNMEHIRRGYYGLLAVNPSGIVPIGPNIDYSSPHDRDRFST

> MD03G1283000 MdGSTF15

MVLKLHGLSVSISTARVVACLHEKSVDFELVPVDLFACENKQPEFLTKNPFGKVPVLEDDDITLFESRAITAYVAEKFKETGHDLIRHENFNEAALVKVWTEVESQQYNPAIDPIIFEFFAKPVVGMEPDQTVMDASLEKLKKVLDVYETRLSNNKFLAGDFYSLADLHHFPGTFYFMKTPWSSLVHDRPHVKAWWEEISSRPASKKVAEGMNFGEV

> MD10G1233200 MdGHR1

MTSLSGAFVRTASVFRNFISRDPNSQFPAEPGRYHLYISYACPWASRCLAYLNIKGLDKAISFTSVKPIWERTKESDEHMGWVFPAFETELAGAEPDPLNGAKSIRELYELASTQYTGKYTVPVLWDKKLKTIVSNESGEIIRMFNTEFNDIAENASLDLYPPHLQSQIDQTNEWIYDKINNGVYKCGFARKQEPYDEAVKQLYEALDKCEEILSKQRYLCGNTLSEADIRLFVTLIRFDEVYVVHFKCNKKVLREYLNLFNYTKDIFQVPGMSSTVNMDHIKRHYYGSHPSINPFGIIPSGPDIDYSSPHDRNRFST

> MD11G1303600 MdGSTF14

MVLKLHGLSVSTCTARVVACLHEKSVDFELVPVDLFACENKQPEFLAKNPFGLIPVLEDDGITLFESRAITAYVAEKFKETGHDLVRRENFNEAALVKVWTEVESQQYNPAIEPIIFEFFAKPVVGIEPDQTVIDASLEKLKKVLDVYEARLSSNKFLAGDFYSLADLHHFPGTFYFMKTPWSSLVDDRPLVKAWWEEISARPASKKVAEGMNFGEV

> MD05G1184300 MdGSTU33

MEEVKLLGAWPSHFCYRVIWALKLKGVEYEYVEENLTTKSERLLQHNPVHKKIPVLVHGGKPIPESTIILEYIEEVWPQNPLLSDDPYERAMARFWIKFEEDKSSVFIAYFRSIGEEQEKAKKEAQEALKTIEHGLGDKKFFGGEKIGLADLALGWIAGWLGAMEESAGLKLLEPNMFPHLQTWIRNFKDVPEIKENIPHHDELLAYFKPLREKFIASSRS

> MD05G1184200 MdGSTU32

MEEVKLFGAWHSPYSSRVVWALELKGVKYEYIEEDLANKSDLLLHYNPVHKKIPVLVHNGKPIAESSVILEYIEETWPQNPLLSNDPHERALARFWLKFIDEKSVPFAKFFVADGEEHEKAAKEVRDLLKILEEQALGEKEFFGGSEIGLADLALGFIASSVGVIEQVVGVKVLQASEFPCLCNWINNFRENPAIKKSLQPADQMFVNYKQKREMILASRP

> MD16G1011600 MdGSTU37

MAQVKLLGLWPSPYVYRVIWALKLKGVEYEYVEEDVLFNKSDELLKYNPVHKKVPVLVHDGKATAESIVILEYIEEAWPHNPLLPKDPHQRAEARFWTKFGEDKNRAFLGFFFATGEEQVKAVKEAQENLKILEEHGLGNKKFFGGDKIGLVDLAFGWIALWLEVLEESAGVKVFEADSFPRLHAWIQSFKESPTIKENLPDRDRSAFLNYFKGRRATIVASAQP

> MD10G1172100 MdGSTU31

MEEVKVLGAWPSPYNFRVIWALELKGVKYEYIEENLVNKSDLLLHYNPVHKKIPVLVHNGKPITESAVILEYIEETWPQNPLLSDDPHERALARFWTKFIDEKGLPYLKFMADGEEHEKAAKEIRDVFKILEEQALGEDDFFGGNEIGLADLALGFIASSFGVIEQVVGVKVLHATEFPRLCNWINKFRENPAIKKSLHPDKMFVFYKQRREMILASRP

> MD10G1172200 MdGSTU36

MAQVKLLGLWPSPYVYRVIWALKLKGVEYDEYVEEDVLFNKSDELLKYNPVHKKVPVLVHDGKATAESIVILEYIEEAWPHNPLLPKDPHQRAEARFWTKFGEDKNRAFLGFFFATGEEQVKAVKEAQENLKILEEHGLGNKKFFGGDKIGLVDLAFGWIALWLEVLEESAGVKVFEADSFPRLHAWIQSFKESPTIKENLPDRDRSAFLNYFKGRRATIVASAQP

> MD00G1136300 MdGSTU28

MSGESVKLLGYWASPFALRVKWALKLKEIEYQYVEEDLPNKSPLLLRYNPVHKKVPVLVHDGKPVAESLIILEYIDETWKHNPILPQDPYERAQARFWARFIDEKCVPAIMSAFTNQGEEKEKAAKEARENLKTLESALAGKDFFGGETVGFVDIAAGWIGLWARIVEEIADVNLIDTETMPLLNAWFGRVLEAPVLKECVPPQDKLLEHNRGFHKILTAASS

> MD08G1244100 MdEF1B1

MALVLHAGKTNKNGYKVLIVAEYSGVKVLAPNFEIGVTNKTPEYLKLNPIGKYPLLITPDGPIFESNAIARYVARLKPDNPLCGSSLIDYAHIEQWIDFGSMEIDANVLNWWLPRVGQRVYLPPAEEFAISSLKRALGALNTHLASNTYLVGHSVTLADIVIGCNLYFGFTEILTKSFTSEFPHVERYFWTLVNQPTFKKVIGDVKQAVSVPPVVSAKKPAQPAKGKAKEEPKKEAKKEPEKPKAEAAEEVEEAPKPKPKNPLDLLPPSKMVLDDWKRLYSNTKTNFREVAIKGFWDMYDPEGYSLWFCEYKYNDENTVSFVTLNKVGGFLQRMDLARKYAFGKMLVIGSEAPFKVKGLWLFRGQEIPKFVMDECYDMELYNWTKVDISDENQKERVNQMIEDQEPFEGEALLDAKCFK

> MD02G1271700 MdEF1B3

MAITFSDLYTEAGLKSLDEFLAGKSYISGDKLTLDDIKVYAAVLEKPAGSFANVSKWYDGVSAQLAANFPGKAAGVRVSSGKAESAAPAPSAAAGGDADDDLDLFGDETEEDKKAEAEMAAAKKASLTIAMAITFSDLYTEAGLKSLDEFLAGKSYISGDKLTLDDIKVYAAVLEKPAGSVANVSKWYDGVSAQLAANFPGKAAGVRVSSGKAESAAPAPSAAAGGDADDDLDLFGDETEEDKKAEAEMAAAKKASTKKKESGKSSVLLDVKPWDDETDMKKLEEAVRSVEKEGLFWGASKLVAVGYGIKKLQIMLTIVDDLVSVDDLIEEQLTAEPRNEYIQSCDIVAFNKI

>AtGSTU1 XP_020885836.1

MTEKEESVKLLGFWASPFSRRVEMALKLKGVPYEYLEEDLPNKTPLLLELNPLHKKVPVLVHNDKILLESHLILEYIDQTWKNNPILPQDPYEKAMARFWAKFIDEQILTLGFRSLVKAEKGREVAIEETREMLTFLEKEVTGKDFFGGKTIGFLDMIAGSMIPFCLARLWEGIGIDMIPGEKFPELNRWIKNLEEVEAVRGCIPPRDKQIERMTKIAETIKSA

>AtGSTU2 NP_180509.1

MAKKEESVKLLGFWISPFSRRVEMALKLKGVPYEYLEEDLPKKSTLLLELNPVHKKVPVLVHNDKLLSESHVILEYIDQTWNNNPILPHDPYEKAMVRFWAKFVDEQILPVGFMPLVKAEKGIDVAIEEIREMLMFLEKEVTGKDFFGGKTIGFLDMVAGSMIPFCLARAWECLGIDMTPEDTFPELNRWIKNLNEVEIVRECIPPKEKHIERMKKIIERAKSTF

>AtGSTU3 NP_180508.1

MAEKEEGVKLIGSWASPFSRRVEMALKLKGVPYDYLDEDYLVVKSPLLLQLNPVYKKVPVLVHNGKILPESQLILEYIDQTWTNNPILPQSPYDKAMARFWAKFVDEQVTMIGLRSLVKSEKRIDVAIEEVQELIMLLENQITGKKLFGGETIGFLDMVVGSMIPFCLARAWEGMGIDMIPEEKFPELNRWIKNLKEIEIVRECIPDREKHIEHMMKIVGRIKAV

>AtGSTU4 NP_180507.1

MAEKEEDVKLLGFWASPFTRRVEMAFKLKGVPYEYLEQDIVNKSPLLLQINPVYKKVPVLVYKGKILSESHVILEYIDQIWKNNPILPQDPYEKAMALFWAKFVDEQVGPVAFMSVAKAEKGVEVAIKEAQELFMFLEKEVTGKDFFGGKTIGFLDLVAGSMIPFCLARGWEGMGIDMIPEEKFPELNRWIKNLKEIEIVRECIPPREEQIEHMKKVVERIKSA

>AtGSTU5 NP_180506.1

MAEKEEVKLLGIWASPFSRRVEMALKLKGIPYEYVEEILENKSPLLLALNPIHKKVPVLVHNGKTILESHVILEYIDETWPQNPILPQDPYERSKARFFAKLVDEQIMNVGFISMARADEKGREVLAEQVRELIMYLEKELVGKDYFGGKTVGFLDFVAGSLIPFCLERGWEGIGLEVITEEKFPEFKRWVRNLEKVEIVKDCVPPREEHVEHMNYMAERVRSS

>AtGSTU6 NP_180505.1

MGKNEEVKLLGIWASPFSRRIEMALKLKGVPYEYLEEDLENKSSLLLALSPIHKKIPVLVHNGKTIIESHVILEYIDETWKHNPILPQDPFQRSKARVLAKLVDEKIVNVGFASLAKTEKGREVLIEQTRELIMCLEKELAGKDYFGGKTVGFLDFVAGSMIPFCLERAWEGMGVEMITEKKFPEYNKWVKKLKEVEIVVDCIPLREKHIEHMNNMAEKIRSA

>AtGSTU7 NP_180503.1

MAERSNSEEVKLLGMWASPFSRRIEIALTLKGVSYEFLEQDITNKSSLLLQLNPVHKMIPVLVHNGKPISESLVILEYIDETWRDNPILPQDPYERTMARFWSKFVDEQIYVTAMKVVGKTGKERDAVVEATRDLLMFLEKELVGKDFLGGKSLGFVDIVATLVAFWLMRTEEIVGVKVVPVEKFPEIHRWVKNLLGNDVIKKCIPPEDEHLKYIRARMEKLNIKSA

>AtGSTU8 NP_187538.1

MNQEEHVKLLGLWGSPFSKRVEMVLKLKGIPYEYIEEDVYGNRSPMLLKYNPIHKKVPVLIHNGRSIAESLVIVEYIEDTWKTTHTILPQDPYERAMARFWAKYVDEKVMLAVKKACWGPESEREKEVKEAYEGLKCLEKELGDKLFFGGETIGFVDIAADFIGYWLGIFQEASGVTIMTAEEFPKLQRWSEDFVGNNFIKEVLPPKEKLVAVLKAMFGSVTSN

>AtGSTU9 XP_020869516.1

MDEEIENKVILHGSYASPYSKRIELALKLKSIPYQFVQEDLQSKSQTLLRYNPVHKKIPVLVHNGKPISESLFIIEYIDETWRNGPHLLPEDPYRRSKVRFWANYIQLHLYDVVIKVVKSEGEEQERALTEVKEKLRVIEKEGLKEIFSDTDGEPTVTNETMSLVDIVMCTLLSPYKAHEEVLGLKIIDPEIVPGVYGWINAINETRVVKDLSLPYEQVLEILRVFRQMSLSRS

>AtGSTU10 NP_177598.1

MEEKKSKVILHGTWISTYSKRVEIALKLKGVLYEYLEEDLQNKSESLIQLNPVHKKIPVLVHDGKPVAESLVILEYIDETWTNSPRFFPEDPYERAQVRFWVSYINQQVFEVMGQVMSQEGEAQAKSVEEARKRFKVLDEGLKKHFPNKNIRRNDDVGLLEITIIATLGGYKAHREAIGVDIIGPVNTPTLYNWIERLQDLSVIKEVEVPHDTLVTFIQKYRQKCLQQAANA

>AtGSTU11 NP_177151.1

MGLMNRSKNDEYVKLLGAWPSPFVLRTRIALNLKNVAYEYLEEEDTLSSESVLNYNPVHKQIPILIHGNKPIRESLNIVMYVDETWLSGPPILPSDPFDRAVARFWDVYIDEHCFTSINGVAVAKGEENINAAIAKLEQCMALLEETFQECSKGRGFFGGENIGFIDIGFGSMLGPLTVLEKFTGVKFIHPENTPGLFHWADRFYAHEAVKPVMPDIEKLVQFARLKFNTSIFK

>AtGSTU12 NP_177150.2

MLKNKKSDNSLSRDTLQIKKRKKTTMAQNGSNTTVKLIGTWASPFAIRAQVALHLKSVEHEYVEETDVLKGKSDLLIKSNPIHKKVPVLIHGDVSICESLNIVQYVDESWPSDLSILPTLPSERAFARFWAHFVDGKLFESIDAVAGAKDDAARMTLAGNLMENLAALEEAFQKSSKGGDFFGGGNIGFVDITVGAIVGPISVIEAFSGVKFLRPDTTPGLIQWAEKFRAHEAVKPYMPTVAEFIEFAKKKFSV

>AtGSTU13 XP_020869675.1

MAQNETVKLIGSWSSPYSLRARVALHLKSVKYEYLDEPDVLKEKSELLLKSNPIHKKVPVLLHGDLSICESLNVVQYVDEAWPSVPLILPSDAYDRASARFWAQYIDDKCFAAVDAVVGAKDDEGKMAAAGKLMECLAILEETFQKSSKGLGFFGGETIGYLDIACSALLGPISVIEAFSGVKFLRQETTPGLIQWAERFRAHEAVKPYMPTVEEVVAFAKQKFNVQ

>AtGSTU14 NP_174034.1

MAQNDTVKLIGCSDDPFSIRPRVALHLKSIKYEYLEEPDDDLGEKSQLLLKSNPIHKKTPVLIHGDLAICESLNIVQYLDEAWPSDPSILPSNAYDRASARFWAQYIDDKCFEAANALTGANNDEERIAATGKLTECLAILEETFQKSSKGLGFFGGETIGYLDIACAALLGPISVIEMFSADKFVREETTPGLIQWAVRFRAHEAVRPYMPTVEEVTELVKQRIEEGFKRNFKSNVSTSEYE

>AtGSTU15 NP_176176.1

MGEREEVKLLGTWYSPVVIRAKIALRLKSVDYDYVEEDLFGSKSELLLKSNPIFKKVPVLIHNTKPVCVSLNIVEYIDETWNSSGSSILPSHPYDRALARFWSVFVDDKWLPTLMAAVVAKSEEAKAKGMEEVEEGLLQLEAAFIALSKGKSFFGGETIGFIDICLGSFLVLLKAREKLKNEKILDELKTPSLYRWANQFLSNEMVKNVVPDIDKVAKFIEEFEDRAQYIRCF

>AtGSTU16 NP_176178.1

MGEKEEVKLLGVWYSPYAIRPKIALRLKSVDYDYVEENLFGSKSELLLKSNPVHKKVPVLLHNNKPIVESLNIVEYIDETWNSSAPSILPSHPYDRALARFWSDFVDNKWFPALRMAAITKSEDAKAKAMEEVEEGLLQLEDAFVSISKGKPFFGGEAIGFMDICFGSFVVLLKAREKFKAEKLLDESKTPSLCKWADRFLSDETVKNVAPEIEKVAEFLQELEVRAQSAASRS

>AtGSTU17 NP_172508.4

MASSDVKLIGAWASPFVMRPRIALNLKSVPYEFLQETFGSKSELLLKSNPVHKKIPVLLHADKPVSESNIIVEYIDDTWSSSGPSILPSDPYDRAMARFWAAYIDEKWFVALRGFLKAGGEEEKKAVIAQLEEGNAFLEKAFIDCSKGKPFFNGDNIGYLDIALGCFLAWLRVTELAVSYKILDEAKTPSLSKWAENFCNDPAVKPVMPETAKLAEFAKKIFPKPQA

>AtGSTU18 XP_002892562.1

MATEDVKLIGSWASVFVMRAKIALHLKSISYEFLQETFGSKSELLLKSNPVHKKMPVLIHADKPVCESNIIVQYIDEAWNSSGPSILPSHPYDRAIARFWAAYIDDQWFISLRSILTAQGEEEKKASIAQVEERTELLEKAFNDCSKGKPFFNGDHIGYLDIALGSFLGWWRVVELDANHKFLDETKTPSLAKWAERFCDDPAVKPIMPEITKLAEFARKLFPKPQA

>AtGSTU19 NP_565178.1

MANEVILLDFWPSMFGMRTRIALREKGVEFEYREEDLRNKSPLLLQMNPIHKKIPVLIHNGKPVNESIIQVQYIDEVWSHKNPILPSDPYLRAQARFWADFIDKKLYDAQRKVWATKGEEQEAGKKDFIEILKTLESELGDKPYFSGDDFGYVDIALIGFYTWFPAYEKFANFSIESEVPKLIAWVKKCLQRESVAKSLPDPEKVTEFVSELRKKFVPE

>AtGSTU20 NP_177958.1

MANLPILLDYWPSMFGMRARVALREKGVEFEYREEDFSNKSPLLLQSNPIHKKIPVLVHNGKPVCESLNVVQYVDEAWPEKNPFFPSDPYGRAQARFWADFVDKKFTDAQFKVWGKKGEEQEAGKKEFIEAVKILESELGDKPYFGGDSFGYVDISLITFSSWFQAYEKFGNFSIESESPKLIAWAKRCMEKESVSKSLPDSEKIVAYAAEYRKNNL

>AtGSTU21 NP_001319402.1

MAAEVILLGFWPSMFGMRTMIALEEKGVKYEYREEDVINNKSPLLLEMNPIHKTIPVLIHNGKPVLESLIQIQYIDEVWSDNNSFLPSDPYHRAQALFWADFIDKKEQLYVCGRKTWATKGEELEAANKEFIEILKTLQCELGEKPYFGGDKFGFVDIVLIGFYSWFPAYQKFGNFSIEPECLKLIAWGKRCMQRESVAKALPDSEKVVGYVLQLKKLYGIE

>AtGSTU22 NP_177956.1

MADEVILLDFWPSPFGVRARIALREKGVEFEYREENLRDKSPLLLQMNPVHKKIPVLIHNGKPVCESMNVVQYIDEVWSDKNPILPSDPYQRAQARFWVDFVDTKLFEPADKIWQTKGEEQETAKKEYIEALKILETELGDKPYFGGDTFGFVDIAMTGYYSWFEASEKLANFSIEPECPTLMASAKRCLQRESVVQSLHDSEKILAFAYKIRKIYCV

>AtGSTU23 NP_177955.1

MEEEIILLDYWASMYGMRTRIALEEKKVKYEYREEDLSNKSPLLLQMNPIHKKIPVLIHEGKPICESIIQVQYIDELWPDTNPILPSDPYQRAQARFWADYIDKKTYVPCKALWSESGEKQEAAKIEFIEVLKTLDSELGDKYYFGGNEFGLVDIAFIGFYSWFRTYEEVANLSIVLEFPKLMAWAQRCLKRESVAKALPDSDKVLKSVSDHRKIILGID

>AtGSTU24 NP_173160.1

MADEVILLDFWASMFGMRTRIALAEKRVKYDHREEDLWNKSSLLLEMNPVHKKIPVLIHNGKPVCESLIQIEYIDETWPDNNPLLPSDPYKRAHAKFWADFIDKKVNVTARRIWAVKGEEQEAAKELIEILKTLESELGDKKYFGDETFGYVDIALIGFHSWFAVYEKFGNVSIESECSKLVAWAKRCLERESVAKALPESEKVITFISERRKKLGLE

>AtGSTU25 NP_173161.1

MADEVILLDFWPSMFGMRTRIALEEKNVKFDYREQDLWNKSPILLEMNPVHKKIPVLIHNGNPVCESLIQIEYIDEVWPSKTPLLPSDPYQRAQAKFWGDFIDKKVYASARLIWGAKGEEHEAGKKEFIEILKTLESELGDKTYFGGETFGYVDIALIGFYSWFEAYEKFGSFSIEAECPKLIAWGKRCVERESVAKSLPDSEKIIKFVPELRKKLGIEIE

>AtGSTU26 NP_173162.1

MANDQVILLDYWPSMFGMRTKMALAEKGVKYEYKETDPWVKTPLLIEMNPIHKKIPVLIHNGKPICESLIQLEYIDEVWSDASPILPSDPYQKSRARFWAEFIDKKFYDPSWKVWATMGEEHAAVKKELLEHFKTLETELGDKPYYGGEVFGYLDIALMGYYSWFKAMEKFGEFSIETEFPILTTWTKRCLERESVVKALADSDRIIEYVYVLRKKFGAA

>AtGSTU27 NP_189966.1

MSEEEVVVLNFWPSMFGARVIMALEEKEIKFEYKEEDVFGQKTDLLLQSNPVNKKIPVLIHNGKPVCESNIIVEYIDEVWKDDKTLRLLPSDPYQKSQCRFWADLIDKKVFDAGRRTWTKRGKEQEEAKQEFIEILKVLERELGDKVYFGGNDNVSMVDLVLISYYPWFHTWETIGGFSVEDHTPKLMDWIRKCLTRPAISKSLPDPLKIFDRVTQIIKVHEFFYGY

>AtGSTU28 NP_175772.1

MGKENSKVVVLDFWASPYAMRTKVALREKGVEFEVQEEDLWNKSELLLKSNPVHKKVPVLIHNNTPISESLIQVQYIDETWTDAASFLPSDPQSRATARFWADYADKTISFEGGRKIWGNKKGEEQEKGKKEFLESLKVLEAELGDKSYFGGETFGYVDITLVPFYSWFYALEKCGDFSVEAECPKIVAWGKRCVERNSVAATLPESEKVYQQVLKLRQIFGVE

>AtGSTF1 P42769.1

MVTVKLYGMAYSTCTKRVYTTAKEIGVDVKIVPVDLMKGEHKEPAYLDNYHPFGVIPVLEDEDGTKIYESRAISRYLVAKYGKGSSLLPSPSDPKAYGLFEQAASVEYSSFDPPASSLAYERVFAGMRGLKTNEELAKKYVDTLNAKMDGYERILSKQKYLAGNDFTLADLFHLPYGAMVAQLEPTVLDSKPHVKAWWAASLRVIPGRLLRNSSKEFM

>AtGSTF2 NP_192161.1

MAGIKVFGHPASIATRRVLIALHEKNLDFELVHVELKDGEHKKEPFLSRNPFGQVPAFEDGDLKLFESRAITQYIAHRYENQGTNLLQTDSKNISQYAIMAIGMQVEDHQFDPVASKLAFEQIFKSIYGLTTDEAVVAEEEAKLAKVLDVYEARLKEFKYLAGETFTLTDLHHIPAIQYLLGTPTKKLFTERPRVNEWVAEITKRPASEKVQ

>AtGSTF3 NP_178394.1

MAGIKVFGHPASTSTRRVLIALHEKNLDFELVHVELKDGEHKKEPFLSRNPFGQVPAFEDGDLKLFESRAITQYIAHRYENQGTNLLPADSKNIAQYAIMSIGIQVEAHQFDPVASKLAWEQVFKFNYGLNTDQAVVAEEEAKLAKVLDVYEARLKEFKYLAGETFTLTDLHHIPVIQYLLGTPTKKLFTERPRVNEWVAEITKRPASEKVL

>AtGSTF4 NP_849581.1

MDCLQMVFKLFPNWKREAEVKKLVAGYKVHGDPFSTNTRRVLAVLHEKRLSYEPITVKLQTGEHKTEPFLSLNPFGQVPVFEDGSVKLYESRAITQYIAYVHSSRGTQLLNLRSHETMATLTMWMEIEAHQFDPPASKLTWEQVIKPIYGLETDQTIVKENEAILEKVLNIYEKRLEESRFLACNSFTLVDLHHLPNIQYLLGTPTKKLFEKRSKVRKWVDEITSREAWKMACDQEKSWFNKPRN

>AtGSTF5 NP_001322019.1

MGINASHVPETCYHHCNQTFESSRQCFKWCQELARKDEYKIYGYPYSTNTRRVLAVLHEKGLSYDPITVNLIAGDQKKPSFLAINPFGQVPVFLDGGLKLTESRAISEYIATVHKSRGTQLLNYKSYKTMGTQRMWMAIESFEFDPLTSTLTWEQSIKPMYGLKTDYKVVNETEAKLEKVLDIYEERLKNSSFLASNSFTMADLYHLPNIQYLMDTHTKRMFVNRPSVRRWVAEITARPAWKRACDVKAWYHKKKN

>AtGSTF6 NP_171792.1

MAGIKVFGHPASTATRRVLIALHEKNVDFEFVHVELKDGEHKKEPFILRNPFGKVPAFEDGDFKIFESRAITQYIAHEFSDKGNNLLSTGKDMAIIAMGIEIESHEFDPVGSKLVWEQVLKPLYGMTTDKTVVEEEEAKLAKVLDVYEHRLGESKYLASDHFTLVDLHTIPVIQYLLGTPTKKLFDERPHVSAWVADITSRPSAQKVL

>AtGSTF7 NP_171791.1

MAGIKVFGHPASTATRRVLIALHEKNLDFEFVHIELKDGEHKKEPFIFRNPFGKVPAFEDGDFKLFESRAITQYIAHFYSDKGNQLVSLGSKDIAGIAMGIEIESHEFDPVGSKLVWEQVLKPLYGMTTDKTVVEEEEAKLAKVLDVYEHRLGESKYLASDKFTLVDLHTIPVIQYLLGTPTKKLFDERPHVSAWVADITSRPSAKKVL

>AtGSTF8 NP_850479.1

MGAIQARLPLFLSPPSIKHHTFLHSSSSNSNFKIRSNKSSSSSSSSIIMASIKVHGVPMSTATMRVLATLYEKDLQFELIPVDMRAGAHKQEAHLALNPFGQIPALEDGDLTLFESRAITQYLAEEYSEKGEKLISQDCKKVKATTNVWLQVEGQQFDPNASKLAFERVFKGMFGMTTDPAAVQELEGKLQKVLDVYEARLAKSEFLAGDSFTLADLHHLPAIHYLLGTDSKVLFDSRPKVSEWIKKISARPAWAKVIDLQKQ

>AtGSTF9 NP_180643.1

MVLKVYGPHFASPKRALVTLIEKGVAFETIPVDLMKGEHKQPAYLALQPFGTVPAVVDGDYKIFESRAVMRYVAEKYRSQGPDLLGKTVEDRGQVEQWLDVEATTYHPPLLNLTLHIMFASVMGFPSDEKLIKESEEKLAGVLDVYEAHLSKSKYLAGDFVSLADLAHLPFTDYLVGPIGKAYMIKDRKHVSAWWDDISSRPAWKETVAKYSFPA

>AtGSTF10 NP_180644.1

MVLTIYAPLFASSKRAVVTLVEKGVSFETVNVDLMKGEQRQPEYLAIQPFGKIPVLVDGDYKIFESRAIMRYIAEKYRSQGPDLLGKTIEERGQVEQWLDVEATSYHPPLLALTLNIVFAPLMGFPADEKVIKESEEKLAEVLDVYEAQLSKNEYLAGDFVSLADLAHLPFTEYLVGPIGKAHLIKDRKHVSAWWDKISSRAAWKEVSAKYSLPV

>AtGSTF11 NP_186969.1

MVVKVYGQIKAANPQRVLLCFLEKDIEFEVIHVDLDKLEQKKPQHLLRQPFGQVPAIEDGYLKLFESRAIARYYATKYADQGTDLLGKTLEGRAIVDQWVEVENNYFYAVALPLVMNVVFKPKSGKPCDVALVEELKVKFDKVLDVYENRLATNRYLGGDEFTLADLSHMPGMRYIMNETSLSGLVTSRENLNRWWNEISARPAWKKLMELAAY

>AtGSTF12 NP_197224.1

MVVKLYGQVTAACPQRVLLCFLEKGIEFEIIHIDLDTFEQKKPEHLLRQPFGQVPAIEDGDFKLFESRAIARYYATKFADQGTNLLGKSLEHRAIVDQWADVETYYFNVLAQPLVINLIIKPRLGEKCDVVLVEDLKVKLGVVLDIYNNRLSSNRFLAGEEFTMADLTHMPAMGYLMSITDINQMVKARGSFNRWWEEISDRPSWKKLMVLAGH

>AtGSTF13 NP_191835.1

MAMKLYGDEMSACVARVLLCLHEKNTEFELVPVNLFACHHKLPSFLSMNPFGKVPALQDDDLTLFESRAITAYIAEKHRDKGTDLTRHEDPKEAAIVKLWSEVEAHHFNPAISAVIHQLIVVPLQGESPNAAIVEENLENLGKILDVYEERLGKTKYLAGDTYTLADLHHVPYTYYFMKTIHAGLINDRPNVKAWWEDLCSRPAFLKVSPGLTVAPTTN

>AtGSTF14 NP_175408.1

MADSKMKLHCGFIWGNSAALFCINEKGLDFELVFVDWLAGEAKTKTFLSTLNPFGEVPVLEDGDLKLFEPKAITRYLAEQYKDVGTNLLPDDPKKRAIMSMWMEVDSNQFLPIASTLIKELIINPYQGLATDDTAVQENKEKLSEVLNIYETRLGESPYLAGESFSLADLHHLAPIDYLLNTDEEELKNLIYSRPNVAAWVEKMKMRPAWLKTVVMKNHIVDLMKQRRLPIKLDSSCHESTVVAQKNAIAIENK

>AtGSTZ1 NP_178344.1

MANSGEEKLKLYSYWRSSCAHRVRIALALKGLDYEYIPVNLLKGDQFDSDFKKINPMGTVPALVDGDVVINDSFAIIMYLDEKYPEPPLLPRDLHKRAVNYQAMSIVLSGIQPHQNLAVIRYIEEKINVEEKTAWVNNAITKGFTALEKLLVNCAGKHATGDEIYLADLFLAPQIHGAINRFQINMEPYPTLAKCYESYNELPAFQNALPEKQPDAPSSTI

>AtGSTZ2 NP_178343.1

MSYVTDFYQAKLKLYSYWRSSCAHRVRIALTLKGLDYEYIPVNLLKGDQSDSDFKKINPMGTVPALVDGDVVINDSFAIIMYLDDKYPEPPLLPSDYHKRAVNYQATSIVMSGIQPHQNMALFRYLEDKINAEEKTAWITNAITKGFTALEKLLVSCAGKYATGDEVYLADLFLAPQIHAAFNRFHINMEPFPTLARFYESYNELPAFQNAVPEKQPDTPSTI

>AtGSTT1 NP_198937.1

MMKLKVYADRMSQPSRAVIIFCKVNGIQFDEVLISLAKRQQLSPEFKDINPLGKVPAIVDGRLKLFESHAILIYLSSAFPSVADHWYPNDLSKRAKIHSVLDWHHTNLRRGAAGYVLNSVLGPALGLPLNPKAAAEAEQLLTKSLSTLETFWLKGNAKFLLGSNQPSIADLSLVCELMQLQVLDDKDRLRLLSTHKKVEQWIENTKKATMPHFDETHEILFKVKEGFQKRREMGTLSKPGLQSKI

>AtGSTT2 NP_198940.3

MKLKVYADRMSQPSRAVLIFCKVNEIQFDEILISLGKRQQLSPEFKEINPMGKVPAIVDGRLKLFESHAILIYLSSAYASVVDHWYPNDLSKRAKIHSVLDWHHTNLRPGASGYVLNSVLAPALGLPLNPKAAAEAENILTNSLSTLETFWLKGSAKFLLGGKQPSIADLSLVCELMQLQVLDDKDRLRLLSPHKKVEQWIESTRKATMPHSDEVHEVLFRAKDRFQKQREMATASKPGPQSKIIQFSSIGGTSDGPNLVQDTTDRKARRRKWSPPDDVILISAWLNTSKDRKVVVYDEQQAHTFWKRIGAHVSNSASLANLPKREWNHCRQRWRKINDYVCKFVGCYDQALNQRASGQSEDDVFQVAYQLYYNNYMSNFKLEHAWRELRHNKKWCSTYTSENSKGGGSSKRTKLNGGGVYSSSCNPESVPIALDGEEQVMDRPLGVKSSKQKEKKVATKTMLEEREADSRSRLENLWVLDEEEQVMDLPLGVKSSKQKERKVATKTMIEEREAANFRSRLGNLWLLKEKEEREADSRSRLENLWALKEKDIEEQKKLTRMEVLKSLLGRRTGETSEKEETLKNKLIDEML

>AtGSTT3 NP_198938.1

MKLKVYADRMSQPSRAVLIFCKVNEIQFDEILIYLANRQQLSPEFKDINPMGKVPAIVDGKLKLSESHAILIYLSSAYPSVVDHWYPTDLSKRARIHSVLDWHHTNLRPGAAGYVLNSVLGPALGLPLNPKAAAEAEQLLTKSLTTLDTFWLKGNAMFLLGSNQPSIADLSLVCELTQLQVLDDKDRLRLLSPHKNVEQWIENTRKATMPHFDEVHEVLFRAKDRCQKQREMATASKPGPQSKIIQFSTIGEKSDDPNLVQNTTDRRKHRRKWSRAEDAILISAWLNTSKDPIVDNEHKACAFWKRIGAYFNNSASLANLPKREPSHCKQRWSKLNDKVCKFVGCYDQALNQRSSGQSEDDVFQVAYQVYTNNYKSNFTLEHAWRELRHSKKWCSLYPFENSKGGGSSKRTKLNNGDRVYSSSSNPESVPIALDEEEQVMDLPLGVKSSKQKEKKVATIITIEEREADSGSRLENLWVLDEEEQVMDRPLGVKSLEQKENKVAPKPTIEEREAADSRSRLENLWALKEKEEREADSRSRLENLWALKEKDIEEQKKLTRMEVLKSLLGRTTDQLSEKEDILKNKLIDEML

>AtGSTL1 CAB86032.1

MALSPPKIFVEDRQVPLDATSDPPALFDGTTRLYISYTCPFAQRVWITRNLKGLQDEIKLVPIDLPNRPAWLKEKVNPANKVPALEHNGKITGESLDLIKYVDSNFDGPSLYPEDSAKREFGEELLKYVDETFVKTVFGSFKGDPVKETASAFDHVENALKKFDDGPFFLGELSLVDIAYIPFIERFQVFLDEVFKYEIIIGRPNLAAWIEQMNKMVAYTQTKTDSEYVVNYFKRFM

>AtGSTL2 NP_191064.1

MSVGLKVSAFLHPTLALSSRDVSLSSSSSSLYLDRKILRPGSGRRWCKSRRTEPILAVVESSRVPELDSSSEPVQVFDGSTRLYISYTCPFAQRAWIARNYKGLQNKIELVPIDLKNRPAWYKEKVYSANKVPALEHNNRVLGESLDLIKYIDTNFEGPSLTPDGLEKQVVADELLSYTDSFSKAVRSTLNGTDTNAADVAFDYIEQALSKFNEGPFFLGQFSLVDVAYAPFIERFRLILSDVMNVDITSGRPNLALWIQEMNKIEAYTETRQDPQELVERYKRRVQAEARL

>AtGSTL3 NP_195899.1

MAPSFIFVEDRPAPLDATSDPPSLFDGTTRLYTSYVCPFAQRVWITRNFKGLQEKIKLVPLDLGNRPAWYKEKVYPENKVPALEHNGKIIGESLDLIKYLDNTFEGPSLYPEDHAKREFGDELLKYTDTFVKTMYVSLKGDPSKETAPVLDYLENALYKFDDGPFFLGQLSLVDIAYIPFIERFQTVLNELFKCDITAERPKLSAWIEEINKSDGYAQTKMDPKEIVEVFKKKFM

>AtDHAR1 NP_173387.1

MALEICVKAAVGAPDHLGDCPFSQRALLTLEEKSLTYKIHLINLSDKPQWFLDISPQGKVPVLKIDDKWVTDSDVIVGILEEKYPDPPLKTPAEFASVGSNIFGTFGTFLKSKDSNDGSEHALLVELEALENHLKSHDGPFIAGERVSAVDLSLAPKLYHLQVALGHFKSWSVPESFPHVHNYMKTLFSLDSFEKTKTEEKYVISGWAPKVNP

>AtDHAR2 NP_177662.1

MALDICVKVAVGAPDVLGDCPFSQRVLLTLEEKKLPYKTHLINVSDKPQWFLDISPEGKVPVVKLDGKWVADSDVIVGLLEEKYPEPSLKTPPEFASVGSKIFGAFVTFLKSKDANDGSEKALVDELEALENHLKTHSGPFVAGEKITAVDLSLAPKLYHLEVALGHYKNWSVPESLTSVRNYAKALFSRESFENTKAKKEIVVAGWESKVNA

>AtDHAR3 NP_568336.1

MISLRFQPSTTAGVLSASVSRAGFIKRCGSTKPGRVGRFVTMATAASPLEICVKASITTPNKLGDCPFCQKVLLTMEEKNVPYDMKMVDLSNKPEWFLKISPEGKVPVVKFDEKWVPDSDVITQALEEKYPEPPLATPPEKASVGSKIFSTFVGFLKSKDSGDGTEQVLLDELTTFNDYIKDNGPFINGEKISAADLSLAPKLYHMKIALGHYKNWSVPDSLPFVKSYMENVFSRESFTNTRAETEDVIAGWRPKVMG

>AtDHAR4 Q9FG59.1

MGIEVCVKAASGAPDVLGDCPFGQRILLTLEDKKLPYKTHLIDVSLKPDWFLAISPKGKLPLVKFDEDENWVADSDLIVGIIEEKYPEPSLVTFPPEFASVGSKIIGAFVMFLTSKDHANDGSDMALLDELEALDHHLKTHVGPFVAGDKVTVVDLSLAPKLYHLETTLGHFMDWCVPESLTNVRDYMKVLFSLESFEKTKAAKEYLIASWAPKLDV

>AtDHAR5 NP_173386.1

MGKSFLIGRRIKIYNLKKKSCYYYWKQAFDKYTNLMSCEDCKIILDIKCSVFLDWPTFCDISPQGKVPVLKIDDKWVTDSDATVGILEEKYPDPPLKTPAEFASVGSNIFEALENHLKSHDGPFIAGERVSAVDLSLAPKLYHLQVALGHFKSWSVPESFPHVHNYMKTLFSLDSFEKTKTEEKCVISGWAPKVNP

>AtTCHQD1 NP_177853.1

MQLYHHPYSIDSQRVRLALEEKGIDYTSYHVNPITGKHMDPSFFRMNPNAKLPVFRNGSHIILDTIEIIEYLERIAEVSSGIEDATFNREVVEWMRKIREWESKLFTLAHIPDNRRLYVSKFLRMVVIARMAESPDLASAYHRKLREAYDTEDKLKDPGALRRSKDHLLRLLDEVETKLEGTTYLAGNEFSMADVMLIPVLARLSLLDLEEEYISSRKNLAEYWALVRRRPSYKKVIGRYFNGWRKYATLVKTWMFVRVRSLLRKY

>AtEF1Bγ1 NP_563848.1

MALVLHTYKGNKSAEKALIAAEYVGVQIDVPSDFQMGVTNKTPAFLKMNPIGKVPVLETPEGSVFESNAIARYVSRLNGDNSLNGSSLIEYAQIEQWIDFSSLEIYASILRWFGPRMGFMPYSAPAEEGAISTLKRALDALNTHLTSNTYLVGHSITLADIITVCNLNLGFATVMTKKFTSEFPHVERYFWTVVNQPNFTKVLGDVKQTEAVPPIASKKAAQPAKPKEEPKKKEAPVAEAPKLAEEEEAPKPKAKNPLDLLPPSPMVLDDWKRLYSNTKSNFREVAIKGFWDMYDPEGYSLWFCDYKYNDENMVSFVTLNKVGGFLQRMDLARKYSFGKMLICGSEGPFKVKGLWLFRGPEIPKFIMDEVYDMELYEWTKVDISDEAQKERVSQMIEDAEPFEGEALLDAKCFK

>AtEF1Bγ2 NP_176084.1

MALVMHTYKGNKGANKALIAAEYAGVKIEESADFQMGVTNKSPEFLKMNPIGKVPVLETPEGPIFESNAIARYVSRKNGDNSLNGSSLIEYAHIEQWIDFSSLEIDANMLKWFAPRMGYAPFSAPAEEAAISALKRGLEALNTHLASNTFLVGHSVTLADIVTICNLNLGFATVMTKKFTSAFPHVERYFWTMVNQPEFKKVLGDAKQTEAVPPVPTKKAPQPAKPKEEPKKAAPVAEAPKPAEEEEAPKPKAKNPLDLLPPSPMVLDDWKRLYSNTKSNFREVAIKGFWDMYDPEGYSLWFCDYKYNDENMVSFVTLNKVGGFLQRMDLARKYSFGKMLICGSEGPFKVKGLWLFRGPEIPKFIMDEVYDMELYEWTKVDISDEAQKERVSQMIEDAEPFEGEALLDAKCFK

>AtGHR1 NP_199315.1

MARSGVDETSESGAFVRTASTFRNFVSQDPDSQFPAESGRYHLYISYACPWACRCLSYLKIKGLDEAITFSSVHAIWGRTKETDDHRGWVFPDSDTELPGAEPDYLNGAKSVRELYEIASPNYEGKYTVPVLWDKKLKTVVNNESSEIIRMFNTEFNGIAKTPSLDLYPSHLRDVINETNGWVFNGINNGVYKCGFARKQEPYNEAVNQLYEAVDRCEEVLGKQRYICGNTFTEADIRLFVTLIRFDEVYAVHFKCNKRLLREYPNIFNYIKDIYQIHGMSSTVNMEHIKQHYYGSHPTINPFGIIPHGPNIDYSSPHDRDRFSS

>AtGHR2 NP_199312.1

MATPMENENPNFARTATSFRNFVSKDPDSQFPAESGRYHLYISYACPWASRCLAILKLKGLDKAISFSSVQPLWRNTKENDEHMGWVFPDSDTEVLGAERDHINGAKSVRELYDIASSNYTGKYTVPVLWDKKLKTIVNNESSEILRMFNTEFNHVAENPSLDLYPPNLRAIIDETNEWIHDGINNGVYKCGFATNQETYDVEVKRLYEALDRCEDILRKQRFLCGNTLTESDIRLFVTVIRFDEAYAVIFKCDKRLVREYYHLFNYTKDIYQIAGMSSTVKMDHIKQNYYGSFPSINPLEIIAHGPNIDYSLPHDRHRFSLESDYTRLELFESASFVCELKLIEIFDSL

>AtGHR3 NP_193723.3

MSYSTIISNTSFLSLASKFTTRGSRLQCTVSMARSAVDETSDSGAFQRTASTFRNFVSKDSNSQFPAESGRYHLYISYACPWASRCLSYLKIKGLDDAISFSSVKPIWGRTKETDEHMGWVFPGSDTEVPGADPDHLNGAKSVRELYEIASPNYTGKYTVPVLWDKKLKTVVNNESAEIIRMFNTEFNHIAGNPDLDLYPSHLQAKIDETNEWIYNGINNGVYRCGFAKKQGPYEEAVEQVYEALDRCEEILGKHRYICGNTLTETDIRLFVTLIRFDEVYAVHFKCNKKLIREYPNLFNYTKDIFQIPGMSSTVNMNHIKQHYYGSHPSINPFGIIPHGPNIDYTSPHDRHRFSK

>AtGHR4 NP_001031671.1

MSYSTIISNTSFLSLASKFTTRGSRLQCTVSMARSAVDETSDSGAFQRTASTFRNFVSKDSNSQFPAESGRYHLYISYACPWASRCLSYLKIKGLDDAISFSSVKPIWGRTKETDEHMGWVFPGSDTEVPGADPDHLNGAKSVRELYEIASPNYTGKYTVPVLWDKKLKTVVNNESAEIIRMFNTEFNHIAGNPDLDLYPSHLQAKIDETNEWIYNGINNGVYRCGFAKKQGPYEEAVEQVYEALDRCEEILGKHRYICGNTLTETDIRLFVTLIRFDEVSSYFQSKKKKYTICERISNVETLIQVYAVHFKCNKKLIREYPNLFNYTKDIFQIPGMSSTVNMNHIKQHYYGSHPSINPFGIIPHGPNIDYTSPHDRHRFSK

>AtGSTM1 NP_565446.1

MEGDQETNVYTLVARKPSFDLPTACPNCLPAYIYLKLAQLPFELAFNSTFPDSDELPYFESDTYVAYNNEDGGVIEKLKKDGIVNLDSQLQSLSDYLSLKALIVSWLEEALTYEIWVGTEGISTSKIYYSDLPWVISKVLFYKQTYLAKNRLGITKENAEQREKQIYKRASEAYEALSTRLGEQKFLFEDRPSSLDAFLLSHILFIIQALPVTSVLRCKLLEHSNLVRYAEKLKSEFLEASSSSPSPPLHSFPSSFPRKSSKPKSKPKVEKTEEEKKFKKRARFFLAAQFLAVVIYVSVMGGGSSDELEYEDEDD

>DcGSTF1 KU565000

MAIKVYGVAMSSNVVRVIAALNEKGLDYELVPVNLAAGEHKKPEFLALNPFGQIPAFEDGDVKIFESRAISRYVATAYKSTGDDLLPAKTPAEVAALECWLEVEPQHISEPISKVLFELQIKPLLSMTTDPATVEAESEKLGKVLDVYEARLSESKYLAGDAFTLADLNLMPYVYNLSLSSKAELMTSRPHFKKWWDEVSARPAWQKTAASIKL

>DcGSTF2 KU565001

MGSLKVFGLPASTDVSRVLTCLFEKEVEFQLIRIDTYKKDHKVPEFLRLQDPSGQVTLKDGIFTFVDSREICRYVCEKYANQGNKTLYGTGGLERASIEQWLQSEARNFDPPSSALVFHLAFAVPMGLEPDEAVVNQNVRKLARVLDVYDQRLEDSKYLAGDEFTLADLSHLPNSHYLVDRSERGRELFYSRKNVARWWKEISSRPSWQQVVHMQSEHPGPLEKFKTID

>DcGSTF3 KU565002

MSPGVKVFGSPTSAEVARVLACLFEKNVEFQLIRVDVYRGRKRMPDYLKLQPSGQALTFEDGYLTMVDSREICRHIAEKNAEKGNKELLGTGTLERASMEQWLLTEAQSFDPPSSALAFHLAFAPMAGIETNGTVVEKSEAKLKNVLDVYEQRLEDNRYLAGDKFTLADLSHLPNAHNLMKIPRCRSLFQSRKRVMEWWGEISNRSSWRKVAEMQKAPPRRIYILSAPSS

>DcGSTF4 KU565003

MAIKVYGVAMSSNVVRVIAALNEKGLDYELVPVNLAAGEHKKPEFLALNPFGQIPAFEDGDVKIFESRAISRYVATAYKSTGDDLLPAKTPAEVAALECWLEVEPQHISEPISKVLFELQIKPLLSMTTDPATVEAESEKLGKVLDVYEARLSESKYLAGDAFTLADLNLMPYVYNLSLSSKAELMTSRPHFKKWWDEVSARPAWQKTAASIKL

>DcGSTF5 KU565004

MASVTVYGPPLSTAVSRVLACLLEKDIQFQLVPVDMAKGQHKSPDFLKIQPFGQVPAFRDESTTLFESRAICRYICDQYADRGNQMLMGRKEGGLVERYWVEQWLEAESQSFNTPSSTLLLQLALAPRMGLKQDPALIEQSEGKLAKVLDVYEQRLGESRFLAGDEFTLADLSHLPNSHYLVNATDRGGLITSRENVGRWWEEISLRESWKKVVEMQQAKAPPS

>DcGSTU1 KU565010

MAEEGLKLYGLKLSPFVLRVEWALKLKGIEYEYVVEDLKNKSPQLLEYNPVYKKVPVLVHHGKPICESTVILEYIEDAWKDKHPSLLPTDPHQRAVARFWAKFSEEKCLRGCSDVFSTTGEEQIKAVEELKERLKTLERYLQGKKFFGGESIGMVDIVTGWITIWYGIIEEIVGVQIIDEKELPLISEWARNFLELEPVKESMPPLDEVKAHLRELRELVLAGQFSA

>DcGSTU2 KU565011

MAEQVKLLGSWLSPFSRRVELALKLKGVAYDYIDEDLANKSTLLLESNPVYKKIPVLIHKGNPIAESLIILEYIEDTWNGHPLLPMDLVERAQARFWAKFIDDKCLMAVWMSCWSEGEAQEKFMKEANENLTILEGALEGKKLFGGDAIGLVDIAANFLSSWAGVLQEIAGISLINEDKHPALWKWSQEFVSSDVVKECLPGREKLRARMLSRKEAILATKAPAY

>DcGSTU3 KU565012

MEEQPSSCLKLLGSWASSYTHRVQLALKLKGIEFEYIEEDLANKSPALLLNNPIYKKVPVLLHRDRPVPESLVILHYIDEAWPSTLPIMPSDPYERALARFWSHFADDKLGPAVGAVFASSGEAQKAAVEQVHDNFRLIESELRDGAFKGRRFFGGDRIGLLDIVLGCGSYWLAVFEEVAEVTLLDPEPFPLFHSWLKDFEGQEEVKEIIPAFDRLLEYAKGVRHMMLSTKANSSTAAGSPVGNCASESSSNTSVTVTAPTDQLGEN

>DcGSTU4 KU565013

MEDEEVVLLSWWASVFSLRVKIALAEKGIDYNKKEEDLFNKSPILVKYNPVHKKVPVLIHNGKPICESLAIVEYIDEVWTSSPSLLPQDPYQRANARFWAGLTDKIYPCARRVRSGEVGEELEAAKVELVGLLKLLEGELGEKPFFGGESFGYIDVALVPFSCWFHTYETRGGFVLDEECPRLMDWVRRCMERDSVSKALPDPCKVFELNRELIKKLAVQQ

>DcGSTU5 KU565014

MATENGVVLLDRWVSPFGQRCRIALAEKGVEYEYREENLVDKKSPLLLKSNPVHKKIPVLLHDGKPVCESLIIVQYIDEVWADKAPILPKDPYARAQARFWADFIDKKIYECGTRLWKLKGEAHEEAKKEFIEILKLLEGELGDKKFFGGDEFGFVDITLVPFTAWFYTYETCANFSVEKEAPKLVAWGKRCMERESVARSLHDPAKVYEFVCMLKKKFGIE

>DcGSTU6 KU565015

MAVSEAREDVKLLGHWVSPFVTKVAIALNLKGVRYQLLEEEIRSKGGLVKSELLLKSNPVYKKIPVLIHNGKPICESMIIVQYIDEVWASRGTSIVPSDPHERAVAQFWSVYADEWVIKLFVMLRATTEEAKAKAAEEAIAGLQVLEEAFMECSKGKSFFGVDSIGYVDIALGCSLGWIGAAEELCGVRVLDEAKTPQLVGWAERFLSDDAVMRVIPEVGKFVEFGKMMRAGRNVEPAAN

>DcGSTU7 KU565016

MATGSTEEAKLLGAWPSPFVMRPRIALNLKGVGYEFLEEAFGIKSDLLLKSNPVYKKIPVLIHNGKPVCESMIIVEYVDEVWADDGKPRILPSDPYDRAIARFWAYYIDDKFFPSVRGLSTAQTEEAKAEVVSQVFAGLQLLEGAFMNCSKGKGFFGGDTIGYLDIALGCYLGWIRVIEKSEGIKFLDEDKTPLLAGWAERFCADAAVKEVMPEIEKLMEFAQALRAKMQAAAAAAAAVQYYYSDAPCRRAQKTGPGTSGWCRAPSLV

>DcGSTU8 KU565017

MSEVRVLGVAGSPFSRRVELALRLKGIPYVYKEEDLLRKSKLLLECNPVYKKVPVLVHGGNAVAESLVILEYIEDTWTAGYHLLPEDPLERAKARFLAKFIDEKCLPAVWMSCWSEGETQKNFIEESKEHLCILEGELKGKKFFGGESIGIADIAANFLTIWVDVLQEIAGLCIISEDTHPVVWRWAREFLKSDVVKECTPKREKLLSMFQAQKDSKMAK

>DcGSTU9 KU565018

DGKPVCESLIIVEYIDEVWAYKAPILPKSPYARAQARFWADFVDKKIYECGTRLWKLKAGEAQEAAKKEFIEHVKLLEGELGDKKYFGGDEFGFVDIALVPFTAWFYSYGTFANFSVEEEVPKLAAWGKRCMERESVAKSLHDPKKVYEFVCMLKKRFGVE

>DcDHAR1 KU565019

MSMAPAIVTRSSSFLLSSRLKTLSSTLSPSLFCRPTRRRTLLTTTIRATSSSPQPLELCVKESPTVPGKLGDCPFSQRVLLTLEEKHLPYDMKLVDLSNKPEWFLKITPEGKVPIAKLDDKWVSDSDVITQSVEEKFPDPPLATPPEKATIGSKIFSTFIAFLISKDSSDGTELALLNELKSFDGYIKENGPFINGERVSAADLSLGPKLYHLGIALGHYKNWSVPESLPYVKRYMKSVFSMDSFVKTKALQEDVIAGWRPKVFG

>DcDHAR2 KU565020

MAPVEVCVKAATGNPDVPGDCPFCQRVLLTLEEKKVPYEMKLVNLTDKPDWFLKISPEGKVPLYKGEDGKWVADSDVITGIIEEKYPDPSLVTPTEYSSVGSKIFPSFVKFLKSKDPNDGTEQALLEELEALDNHLKARGPYVSGEKLTAVDLNLAPKLFHLEIVLDHFKGWKVPENLTNVHSYMQLLFNRESFVKTKAAREQVIAGWAPKVNA

>DcTCHQD1 KU565021

MQLYHHPYSVDSQKVRLALEENGIDYTSFHVNPLTGKNMDSSFFRMNPSAKLPVFQNGSQIIYQAMDIILYIDRLIVSLDGKASSITTQVMDWMLKIEEWNPKIFTLSHIPDKYRLFVSKFVRRVVIARMSEAPDLASVYHVKLRDAYETEDKLKNPDIVKQSEEKLLRLLDDAEMQLQETRYLAGEEFTLADAMFIPVLSRITLLNLEEEYVSCRPKIAGYYELVKRRPSYKKVIGKHFSGWRWYRTLVKTVFFLCIRSMFRRY

>DcGSTL1 KU565022

MATTAGYRNEVLPPILDATADPPSLFDGTTRLYISYICPFAQRTWIARNYKGLQEKIKLVPIDLQNRPAWYKEKVYSGNKVPSLEHNNAVKGESLDLIRYIDENFEGPKLFPDDPAKQEFAEELLSYSDAFNQVNFSAFTVKGDVPDEVGAAYDKLEEALAKFDDGPFFLGQFSLVDIAYAPFVERVQYFFSEVKNYDIASGRPRLTKWIEELNKVDAYTATKRDPQELVGAYKKKFGIE

>DcGHR1 KU565023

MLTSLSLQLPFRPFPPRQPFSTLKNTIQMARSALDEMTETGAFDRTPSTFRNFISKDKASQFPAESGRYHLYISYACPWASRCLSFLKLKGLENAITYTSVKPKWERTKETDDHFGWVFPQSSEEEAGADPDPFYGAKSVRELYDLASSNYSGKYTVPVLWDKKLKTIVNNESAEITRMLNTEFNEIAENADLDLYPSRLQETIDGVNAWVYDAINNGVYKCGLAKKQGPYDEAVAKLYEALDKCEAILANQRYICGNVLTEADIHLFVTLIRFDEVYAVHFKCNKKLLREYPNLFNYTKDIYQIPGMSSTVCMDHIKKHYYGSHPTINPYGIIPVGSNTDFSSPHDRERFGR

>DcGSTT1 KU565024

MALKLYVDRMSQPSRAILIFCKMNKIDFEEVRIDLAKGQHRSPEFKEINPMGQVPAIVDGRFRLSESHAILIYLACVFPGVSDHWYPADLFSRAKINSVLDWHHSNLRRGAAAYVLNSTLAPVLGLPLNPQAANEAGKLLCESLSKIESIWLKGNAKFLLGNTQPSIADLSLVCEIMQLEVVNEEDRQRILDPHLKILQWIENVKNYTSPHFEEVHEVLYKVKERLQRRLSSGAKLNSKL

>ZmGSTU1 NP_001104989.1

MADAGNEAEGLTLLGLHVSPFALRVRMALSLKGLSYEYIEQDLFHKGELLLSSNPVHKKVPVLIHHGKPICESLAVVEYVDEVWPGAAATILPADPHGRATARFWAAYIDGKLFPAWTGIMKAATEEARADKLRETHAAVLNLEKAFAEISSSSSNDGAAFFGGDSVGYLDLALGCSLPWFGALRAMLGVEIIDAAQAPLLVAWAERFGETPVAKEVLPQPDEAVAYAKKIQAYWASAKN

>ZmGSTU2 AQK85998.1

MEKTSENAIPPAASPLMLFGSWASSYTHRVQLALRLKGLEYDYVEEDLGNKSDELLRHNPVHKKVPVLVHGGRALPESVIILQYLDDAWPETRPLLPADAFDRALARFWCHFADDKVHMYIYTPPSSCRASAERGGDACLSLPDTYVRARLVRFCPRACLVAARAGGGRGVRVDGGGPGGGGAAGAREPGADRVRAPRRRVPGPPLLRRRRGGPAGRRPGLRLLLARRVRGGDRGAPRGRRRLPALPRLAARLRGPGRGPGDHPRGGPPARVRARPPPHAARPRGGRRRRRRGRALHRRPRRTAGLG

>ZmGSTU3 AQK85978.1

MEKTSENAIPPAASPLMLFGSWASSYTHRVQLALRLKGLEYDYVEEDLGNKSDELLRHNPVHKKVPVLVHGGRALPESVIILQYLDDAWPETRPLLPADAFDRALARFWCHFADDKVHMYIYTPPSSCRASAERGGDACLSLPDTYVRARLVRFCPRACLVAARAGGGRGVRVDGGGPGGGGAAGAREPGADRVRAPRRRVPGPPLLRRRRGGPAGRRPGLRLLLARRVRGGDRGAPRGRRRLPALPRLAARLRGPGRGPGDHPRGGPPARVRARPPPHAARPRGGRRRRRRGRALHRRPRRTAGLG

>ZmGSTU4 NP_001105554.2

MAEAEATVGRLMLYSYWRSSCSHRARIALNLKGVDYEYKAVNLLKGEQSDPEFVKLNPMKFVPALVDGSSVIGDSYAITLYLEDKYPEPPLLPQDLQKKALNHQVTWLLKLAGGNSGQNIVTVRIK

>ZmGSTU5 NP_001104985.2

MQVAMAGETKKGLVLLDFWVSPFGQRCRIALAEKGIAYEYSEQELLGGAKSDILLRSNPVHKKIPVLLHDGRPVCESLVILEYLEEAFPEASPRLLPDAAYARAQARFWAAYSDKVYEAGTRLWKLRGDARAQARAEIVQVVRNLDGELGDKAFFGGEAFGFVDVALVPFVPWLPSYERYGDFSVAEIAPRLAAWARRCAQRESVARTLHPPEKVDEFINLLKKTYGIE

>ZmGSTU6 NP_001152168.1

MAAAGGGVGDSELRLLGKSSSPWVFRVRVALGLRGLSYEYIEEDLGNKSELLLRSNPVHKKVPVLIHGGRPVCESLVILQYVDEIWRGTGPPLLPSDPYDRATARFWAAYVDDKVRYSGGDESVLELSDSAVLPFPFPVSSSSRRSARCSGRGRTSRERRRSRTPSSWRRRWSGRSRSAPGGRRSSAATPSGSWTSRSGATRFGSERWTRRRAPTFWTGPGSLTWRRGRSGSWPSAPSTRWCRTPGSFWSSTGRLGPNGLPLLILAELMNTLDACCLHWLLVLDTFSDTVSNRNKYIKASLETLVLLRFLKIE

>ZmGSTU7 NP_001153168.1

MAAAGGGVGDSELRLLGKSSSPWVFRVRVALGLRGLSYEYIEEDLGNKSELLLRSNPVHKKVPVLIHGGRPVCESLVILQYVDEIWRGTGPPLLPSDPYDRATARFWAAYVDDKVRYSGGDESVLELSDSAVLPFPFPVSSSSRRSARCSGRGRTSRERRRSRTPSSWRRRWSGRSRSAPGGRRSSAATPSGSWTSRSGATRFGSERWTRRRAPTFWTGPGSLTWRRGRSGSWPSAPSTRWCRTPGSWSSTGRLGPNGLPLLILAELMNTLDACCLHWLLVLDTFSDTVSNRNKYIKASLETLVLLRFLKIE

>ZmGSTU8 NP_001152486.1

MAAAGGGVGDSELRLLGKSSSPWVFRVRVALGLRGLSYEYIEEDLGNKSELLLRSNPVHKKVPVLIHGGRPVCESLVILQYVDEIWRGTGPPLLPSDPYDRATARFWAAYVDDKVRYSGGDESVLELSDSAVLPFPFPVSSSSRRSARCSGRGRTSRERRRSRTPSSWRRRWSGRSRSAPGGRRSSAATPSGSWTSRSGATRFGSERWTRRRAPTFWTGPGSLTWRRGRSGSWPSAPSTRWCRTPGSFWSSTGRLGPNGLPLLILAELMNTLDACCLHWLLVLDTFSDTVSNRNKYIKASLETLVLLRFLKIE

>ZmGSTU9 NP_001306693.1

MAGGDDLKLLGLWASPYVLRVKLALSLKGLSYENVEEDLRDKSELLLKSNPVHQKVPVLIHGGKPICESQVILQYIDEAFAGTGPSLLPADPYERAVARFWAAYIDDKMLPAWNQSTMGKTEEERAEGKKQSVVTVETLEGALRDCGGQGKPFFGGDSVGYVDVVLGGLLGWVRASEELHGVRPFDPERTPLLAAWSERFGALDAVQTVMPDVGRLLEFGKALMARLAAAAAAGASNN

>ZmGSTU10 ONM39050.1

MAAGGELQLLSSWYSPYVIRAKVALGLKGLIYEFIEEDLFSKSDLLLKLNPVHKKVPVLVHGGRPVCESLVIVQYVDETWAAGTGTPLLPADAHDRATARFWAAYVDDKFCKEWIKLYRSTTAEKAAEALGGVVPVVETLEQAFRECSRGKPFFGGDAVGLVDIALGSFVVWIRVVDEAAGVKLLDEAKFPALTAWAERFLAVDAVREVMPDAGRLLEHYKGFLAKRAPPSPAGY

>ZmGSTU11 AAG34828.1

MADGGELQLLGSWYSPYVIRAKVALGLKGLSYEFVEEDLSRKSDLLLKLNPVHRKVPVLVHGGRPVCESLVILQYVDETWAGTGTPLLPADAYDRAMARFWAAYVDDKVITVTAEAGRPA

>ZmGSTU12 NP_001104997.2

MAGEEGLKVLGLQVSPFVLRVCLALNMKGVSYEYVEEDISNKSELLLKSNPVHKKVPVLIHNGKPICESLVIMQYVDELFAGRPILPTDPYERATARFWAAYADDKLFPAWYGMVKAQAEEERAEKAKETLAAIEHMEVTFAKCSGGNAFFGGDSIGYVDIVLTCSAPSCSGSRRCAGFSTWRSLTLARLRCWLRGRSGL

>ZmGSTU13 NP_001104997.2

MAGEEGLKVLGLQVSPFVLRVCLALNMKGVSYEYVEEDISNKSELLLKSNPVHKKVPVLIHNGKPICESLVIMQYVDELFAGRPILPTDPYERATARFWAAYADDKLFPAWYGMVKAQAEEERAEKAKETLAAIEHMEVTFAKCSGGNAFFGGDSIGYVDIVLTCSAPSCSGSRRCAGFSTWRSLTLARLRCWLRGRSGL

>ZmGSTU14 XP_008674526.1

MSPPVKIIGHFTSPFSHRVEAALRLKGVPYELVQEDLSNKSELLLAKNPVHKKVPVLLHGDRAICESLAIVQYVDEAFDGPPLLPADPHDRAVARFWADFMDKLVVPFWMAHWAEGEAQKALVEEAKQKLALLEVQLQGKRFFGGDTLGYVDIAAGVLGPWLSMVEEVTGVAVLDEDEYPALRRWSEEYNSYEALRQCVPDRDQLVAFYTENKDKYKMFAKAWLKQ

>ZmGSTU15 NP_001131533.2

MALVLHSGAGNKNAFKALIAAEYSGIKVELTKDFEMGVSNKTPEFLKMNPLGKVPVLETPDGPVFESNAIARYVARLKDDNPLFGSSRIEQAHVEQWMDFAATEVDPGVAWYLYPRLGYLPYVSTTEETAISSLKRSLGALNTHLASNTYLVGHAVTLADIVMTCNLYHGIARILTKTFTSDFPHVERYFWTMVNQPNFKKVIGEVKQAESAPPIPKKAAPSKEPKAKDVKKEAPKEAPKPKVVEAPAEEEAPKPKPKNPLDLLPPSKMVLDDWKRLYSNTKTNFREVAIKGFWDMYDPEGYSLWFCDYKYNDENTVSFVTLNKVGGFLQRMDLCRKYAFGKMLVIGSEPPFKLKGLWLFRGQDVPKFVMDEVYDMELYEWTKVDISDEAQKERVNAMIEDQEPFEGEALLDAKCFK

>ZmGSTU24 ONM39050.1

IRAKVALGLKGLIYEFIEEDLFSKSDLLLKLNPVHKKVPVLVHGGRPVCESLVIVQYVDETWAAGTGTPLLPADAHDRATARFWAAYVDDKFCKEWIKLYRSTTAEKAAEALGGVVPVVETLEQAFRECSKGKPFFGGDAVGLVDIALGSFVVWIRVVDEAAGVKLLDEAKFPALTAWAERFLAVDAVKEVMPDAGRLLEHYKGFLAKRSPPAGY

>ZmGSTU25 ONM39033.1

MIEQSKHLQTVMAAPVGANAIGAGDELLLLGTWSSPWVIRARVAMGLKGLSYEYLEEDLGNKSGLLLRSNPVHKKVPVLIHGGRPLCESLVILQYLDEAWPTAGPPLLPADPYDRATARFWAAYINDTFFPAFRALFRSLTDEQRAEALRNALPAVETLERTLAECSAGKAFFGGDAVGIVDVALGSHLVWIRVVDEVGGTSLLDAAKVPGLAAWAERFLAVDAVKKVMPDATKVLEQYKGFRAKWIAGAGCT

>ZmGSTU26 NP_001266752.2

MTHATGPSLWGCQGCRRTGPPKIEGPVATDMSRLTTTETVVLPVELSELGKKMSEAAVRVIGLWPSPFVIRVLIALKLKGVEFEFVEEVVGRKSELLLRSNPVHKKIPVLLHHGKPISESLIIVQYIDEVWSSGAPAFLPVDAHARAVQRFWAQYVDDKLPWAIRILKGTDDGGMEQAAGQLSAALQLLEEAFAQLSQGKRYFGGDSVGYLDIALVSHVGWVKAVEKIAGVTLLDKAKVPNLVAWADRLCAHPAVVDAIPDADKFVEFSVTYGSFSKPINAPAK

>ZmGSTU27 ACF84945.1

MTHATGPSLWGCQGCRRTGPPKIEGPVATDMSRLTTTETVVLPVELSELGKKMSEAAVRVIGLWPSPFVIRVLIALKLKGVEFEFVEEVVGRKSELLLRSNPVHKKIPVLLHHGKPISESLIIVQYIDEVWSSGAPAFLPVDAHARAVQRFWAQYVDDKLPWAIRILKGTDDGGMEQAAGQLSAALQLLEEAFAQLSQGKRYFGGDSVGYLDIALVSHVGWVKAVEKIAGVTLLDKAKVPNLVAWADRLCAHPAVVDAIPDADKFVEFSVTYGSFSKPINAPAK

>ZmGSTF1 NP_001105626.1

MATPAAVMKLYGWAISPFVSRALLALEEAGVDYELVPMSPQAGDHRRPEHLARNPFAMVPVLEDGDLTLFESRAIARHVLRKHRPELLGAGAGGSLERAAMVDVWLEVEAHQLSPPAVAIVVECFAAPLLGRERDQTVVDENVEKLRKVLEVYEARLGECRYLAGDFLSLADLSPFTIMHCIMATEYAAALVEALPRVSAWWEGLAARPAAKKVAEFIPVGAAGLLEHPPKQQD

>ZmGSTF2 NP_001105366.1

MATPAVKVYGWAISPFVSRALLALEEAGVDYELVPMSRQDGDHRRPEHLARNPFGKVPVLEDGDLTLFESRAIARHVLRKHKPELLGGGRLEQTAMVDVWLEVEAHQLSPPAIAIVVECVFAPFLGRERNQAVVDENVEKLKKVLEVYEARLATCTYLAGDFLSLADLSPFTIMHCLMATEYAALVHALPHVSAWWQGLAARPAANKVAQFMPVGAGAPKEQE

>ZmGSTF3 NP_001105720.2

MAPPMKVYGWAVSPWMARALVCLEEAGADYEIVPMSRCGGDHRRPEHLAKNPFGEIPVLEDGDLTLYQSRAIARYVLRKLKPELLREGDLEGSAMVDAWMEVEAHHMEPALWPIIRHSIIGQYVGRDRDHQAVIDENLDRLRKVLPAYEARLSVCKYLAGDVISAADLCHFGFMRYFMATEYAGLVHAYPHVKAWWDALLARPSVQKVMAGMPPDFGYASGNIP

>ZmGSTF4 ACG25283.1

MAPMKLYGAVMSWNVTRCATALEEAGSDYEIVPINFATAEHKSPEHLVRNPFGQVPALQDGDLYLFESRAICKYAARKNKPELLREGNLEEAAMVDVWIEVEANQYTAALNPILFQVLISPMLGGTTDQKVVDENLEKLKKVLEVYEARLTKCKYLAGDFLSLADLNHVSVTLCLFATPYASVLDAYPHVKAWWSGLMERPSVQKVAALMKPSA

>ZmGSTF5 NP_001105111.2

MAPLKLYGMPLSPNVVRVATVLNEKGLDFEIVPVDLTTGAHKQPDFLALNPFGQIPALVDGDEVLFESRAINRYIASKYASEGTDLLPATASAAKLEVWLEVESHHFYPNASPLVFQLLVRPLLGGAPDAAVVDKHAEQLAKVLDVYEAHLARNKYLAGDEFTLADANHASYLLYLSKTPKAGLVAARPHVKAWWEAIVARPAFQKTVAAIPLPPPPSSSA

>ZmGSTF6 PWZ58038.1

MQPVKVYADRRSQPSRAVIIFCRVNQIDFEEVTVDLFKSQHLTPEFKKINPMGQVPAIVDGRFKLFESHAILRYLASVFPGVADHWYPSDLFTRAKIESILDWHHSNLRRGAATLVMHTALLPSLVLRQS

>ZmGSTF7 ACF80172.1

MQISLFFSLLSFFRQQRCNKNTDLQLRVQAEMHTFAKGQIAEALGYILGVTSAYACLAGKRKPKRSYYLRRPISLLCVWCSSLTESRAICRYICDQYADSGNQALFGKKEDGAVGRAAIEQWIESEGQSFNPPSLAIIFQLAFAPMMGRTTDLAVVEQNEAKLAKVLDVYDQRLGESQYFAGDDFSLADLVHLPNADFLVNRTSKAGLITERKNLARWWDDVSSRPAWKKVTEMQSTPRPS

>ZmGSTZ1 XP_008661994.1

MAASKPILYSAWISSCSFRVRIALNLKGVDYEYRAVTRNDLDYEKINPIKYVPALVDGDFVVSDSLAIILYLEDKYPQHPLLPQDLKKKAINLQIANIVCSSIQPLQCYAVIGLLDGTLGSNQSLQIVRNYTDKGFKAIEKLLEGCDSKYATGDEVQLADVFLAPQMYAGVTRFGIDMSKYPLLERFYKAYMEIPAFQVAVPEKQPDAPASPY

>ZmGSTZ2 XP_008661994.1

MAASKPILYSAWISSCSFRVRIALNLKGVDYEYRAVTRNDLDYEKINPIKYVPALVDGDFVVSDSLAIILYLEDKYPQHPLLPQDLKKKAINLQIANIVCSSIQPLQCYAVIGLLDGTLGSNQSLQIVRNYTDKGFKAIEKLLEGCDSKYATGDEVQLADVFLAPQMYAGVTRFGIDMSKYPLLERFYKAYMEIPAFQVAVPEKQPDAPASPY

>ZmGSTZ3 XP_008661994.1

MAASKPILYSAWISSCSFRVRIALNLKGVDYEYRAVTRNDLDYEKINPIKYVPALVDGDFVVSDSLAIILYLEDKYPQHPLLPQDLKKKAINLQIANIVCSSIQPLQCYAVIGLLDGTLGSNQSLQIVRNYTDKGFKAIEKLLEGCDSKYATGDEVQLADVFLAPQMYAGVTRFGIDMSKYPLLERFYKAYMEIPAFQVAVPEKQPDAPASPY

>MdGSTF6

MVVKVYGPVMAACPQRVMVCLLEKGVNFEIVDVDLEAGEHKRPEFLTRQPFGQVPVVEDGDFRLFESRAIIRYYAAKYAG

RGPNLLGTTLEEKAVVDQWLEVEGHNFNDLVYTLVLQLLVLPRMGQRGDAALINACEEKLEKVFDVYEERLSKSKYLAGE

TFTLADLSHLPGIRYLIDEAKLGHLVTGRKKVNAWWEDISNRPSWKKLMQLASDY

>AtTT19

MVVKLYGQVTAACPQRVLLCFLEKGIEFEIIHIDLDTFEQKKPEHLLRQPFGQVPAIEDGDFKLFESRAIARYYATKFADQGTNLLGKSLEHRAIVDQWADVETYYFNVLAQPLVINLIIKPRLGEKCDVVLVEDLKVKLGVVLDIYNNRLSSNRFLAGEEFTMADLTHMPAMGYLMSITDINQMVKARGSFNRWWEEISDRPSWKKLMVLAGH

>VvGSTF12 NP_001267869.1

mvmkvygpvraacpqrvlaclvekgvefevvhvdldsgeqkrpdfllrqpfgqvpvvedgdfrlfesraivryiaakyaeqgpdllgksleekavvdqwleveahnfnelvytlvmqlvilprmgergdlalahtceqklekvfdvyeqrlsksrylagdsftladlshlpairylvkeagmahlvterksvsawwedisnraawkkvmelaa

>ZmBZ2 NP_001183661.1

mptevtstpasrpdcsssssmtagtmrvlggevspftararlaldlrgvayelldeplgpkksdrllaanpvygkipvlllpdgraicesaviiqciedvargsggaeasslllpddpyeramhrfwtafiddkfwpaldavslaptpgaraqavedtraalsllevafkdrsngraffsggdaapglldlalgcflpalracerlhglslidasatplldgwsqrfaahpaakrvlpdtekvvqftrflqgqfrvhvs

>PhAN9 CAA68993.1

mvvkvhgsamaacpqrvmvclielgvdfelihvdldsleqkkpeflvlqpfgqvpviedgdfrlfesraiiryyaakyevkgskltgttleekalvdqwlevesnnyndlvynmvlqllvfpkmgqtsdltlvtkcanklenvfdiyeqrlskskylageffsladlshlpslrflmneggfshlvtkrkclhewyldissrdswkkvldlmmkkiseieavsipakeeakv

>FvRAP

MQFSSHEDEDEDEGNRLLNDFLCSDTTYYRPVFLYSSQMVLKVYGPVRAACPQRVMVCLLELGVEFEIVPVDLQAGEQKQPHILARQPFGQVPAIEDGDFKLFESRAIVRYYAAKYAERGPNLLGTTLEEKALVDQWLEVESHNFNDLVFTVVLQLVILPSMGQPGDLALVRSCEEKLKKVFDVYEERLSKSTYLAGNYFSLADLSHLPAIRFLVDEFKMGHLITERKNVNAWWKDISNRPAWKKLMKLAQY

>PpRiant ALE31200.1

mvvkvygpvkaacpqrvmvllekgvnfeivdvnlevgeqkqpqflsrqpfgqvpavedgdfrlfesraiiryyaakyadrgpnllgttleekalvdqwleveahnfndlvytlvlqllvlpdrmgergdvalvhaceeklekvfdvyeerlskssylageaftladlshlpgisylideaklghlvserknvnawwkdisnrpawkklmslasdy

Table S3. Promoter analysis of *MdGSTF6* using PLACE online software.

| Motif Name | Location in promoter | Sequence | Function |
| --- | --- | --- | --- |
| ABRE | -186 | CACGTG | Abscisic acid responsiveness |
| CGTCA-motif | -316,-1062,-1469 | CGTCA | MeJA-responsiveness |
| HSE | -343,-1004 | AAAAAATTTC | Heat stress responsiveness |
| LTR | -80 | CCGAAA | Low-temperature responsiveness |
| MBS | -1151 | CAACTG | MYB binding site |
| TATC-box | -1272 | TATCCCA | Gibberellin-responsiveness |
| TGA-element | -376,-1489 | AACGAC | Auxin-responsive element |
